# A standardized clinical database for research in Chagas disease: The NHEPACHA network

**DOI:** 10.1371/journal.pntd.0012364

**Published:** 2024-08-15

**Authors:** Adriana González Martínez, Irene Losada-Galván, Juan Carlos Gabaldón-Figueira, Nieves Martínez-Peinado, Roberto Magalhães Saraiva, Marisa Liliana Fernández, Janine M. Ramsey, Oscar Noya-González, Belkisyole Alarcón de Noya, Alejandro Gabriel Schijman, Soledad Berón, Marcelo Abril, Joaquim Gascón, Sergio Sosa-Estani, María Jesús Pinazo, Julio Alonso-Padilla, Alejandro Marcel Hasslocher-Moreno, On behalf of the NHEPACHA network (Nuevas Herramientas para el diagnóstico y la evaluación del paciente con enfermedad de Chagas)

**Affiliations:** 1 Departamento de Investigación, Salvando Latidos A.C., Guadalajara, Mexico; 2 Departamento de Investigación, Instituto Cardiovascular de Mínima Invasión (ICMI), Guadalajara, Mexico; 3 Barcelona Institute for Global Health (ISGlobal), Hospital Clínic—Universitat de Barcelona, Barcelona, Spain; 4 Hospital Universitario 12 de Octubre, Madrid, Spain; 5 Secció de Parasitologia, Departament de Biologia, Sanitat i Medi Ambient, Facultat de Farmàcia i Ciències de l’Alimentació, Universitat de Barcelona, Barcelona, Spain; 6 Evandro Chagas National Institute of Infectious Diseases, Fundação Oswaldo Cruz, Rio de Janeiro, Brasil; 7 Instituto Nacional de Parasitología Dr M. Fatala Chabén, Administración Nacional de Laboratorios e Institutos de Salud Dr C. Malbrán, Ministerio de Salud, Buenos Aires, Argentina; 8 Centro Regional de Investigación en Salud Pública (CRISP), Instituto Nacional de Salud Pública (INSP), Tapachula, Chiapas, México; 9 Cátedra de Parasitología, Escuela “Luís Razetti” Facultad de Medicina, Universidad Central de Venezuela, Caracas, Venezuela; 10 Instituto de Medicina Tropical, Facultad de Medicina, Universidad Central de Venezuela, Caracas, Venezuela; 11 Centro para Estudios Sobre Malaria, Instituto de Altos Estudios “Dr. Arnoldo Gabaldón”, Ministerio del Poder Popular para la Salud (MPPS), Caracas, Venezuela; 12 Laboratorio de Biología Molecular de la Enfermedad de Chagas, Instituto de Investigaciones en Ingeniería Genética y Biología Molecular “Dr. Héctor N. Torres”—INGEBI-CONICET, Buenos Aires, Argentina; 13 Fundación Mundo Sano, Buenos Aires, Argentina; 14 CIBER de Enfermedades Infecciosas, Instituto de Salud Carlos III (CIBERINFEC, ISCIII), Madrid, Spain; 15 Drugs for Neglected Diseases Initiative (DNDi), Geneva, Switzerland; 16 Centro de Investigaciones Epidemiológicas y Salud Pública, Consejo Nacional de Investigaciones Científicas y Técnicas, Buenos Aires, Argentina; Universidade Federal de Minas Gerais, BRAZIL

## Abstract

The NHEPACHA Iberoamerican Network, founded on the initiative of a group of researchers from Latin American countries and Spain, aims to establish a research framework for Chagas disease that encompasses diagnosis and treatment. For this purpose, the network has created a questionnaire to gather relevant data on epidemiological, clinical, diagnostic, and therapeutic aspects of the disease. This questionnaire was developed based on a consensus of expert members of the network, with the intention of collecting high-quality standardized data, which can be used interchangeably by the different research centers that make up the NHEPACHA network. Furthermore, the network intends to offer a clinical protocol that can be embraced by other researchers, facilitating comparability among published studies, as well as the development of therapeutic response and progression markers.

## Author summary

In this work, we present a new clinical questionnaire for the standardized evaluation and collection of clinical data from patients with acute or chronic *Trypanosoma cruzi* infection, as well as a uniformed database to collect it. The questionnaire has been produced after consensus from clinical experts belonging to the NHEPACHA network, a coalition of clinical research institutions in Latin America and Spain. This tool will facilitate the collection and sharing of high-quality clinical and paraclinical data useful to annotate sample collections, and allow clinically meaningful comparisons between different cohorts.

## Introduction

The World Health Organization includes Chagas disease (CD), caused by the protozoan *Trypanosoma cruzi* (*T*. *cruzi*), as one of the 20 neglected tropical diseases [1]. Originally emerging from endemic regions in the Americas, primarily in Latin America, CD impact expanded to the northern hemisphere, and currently represents a public health issue in the United States of America, Europe, and Japan [2]. The presence of the disease in non-endemic developed countries has prompted the involvement of diverse government health institutions and research centers into control, diagnosis, and treatment efforts [3,4].

CD has 2 well-defined clinical stages: the acute stage, which occurs shortly after infection, and the chronic stage, which extends throughout the lives of people with the infection. In the chronic stage, approximately two thirds of the individuals do not develop any apparent clinical disease, or detectable signs of organ damage. The remaining individuals may exhibit cardiac and/or digestive tissue damage leading to their dysfunction. Thus, the chronic stage is categorized into clinical forms referred to as indeterminate, cardiac, digestive, and mixed [5]. The chronic stage is not static, which means that patients can progress from the indeterminate to symptomatic forms associated with organ damage, at an estimated average annual rate of 1.9% [6]. Similarly, individuals can also progress between different clinical stages of cardiac and digestive disease [7,8]. One of the main limitations to manage people at indeterminate or at symptomatic stage is that there are no progression markers identified to assess clinical progression [9].

Regarding the etiological treatment of CD, guidelines unequivocally recommend mandatory treatment during the acute stage, including reactivation of chronic infections, mother to child transmission cases, and cases in children and adolescents. Similarly, they strongly recommend the treatment of women at childbearing age and for *T*. *cruzi*-infected adults up to 50 years of age with normal myocardial function [10]. Nowadays, several studies have shown that the use of trypanocidal drugs can alter the natural history of CD, reducing the risk of disease progression [11–13], reducing the mean parasite load, and thereby reducing transmission risk [14].

Medical evaluation is essential to clinically characterize *T*. *cruzi* infection/CD stage, determining the degree of cardiac and/or digestive involvement, stratifying risks, and guiding patient treatment. Clinical evidence is crucial for guiding physicians in this process, assisting them in choosing the most effective diagnostic and therapeutic approaches. It is important to note that medical evidence is dynamic and continually evolving as new research is conducted [15].

Clinical research in CD began in the 1920s with the pioneering work of Carlos Chagas and Eurico Vilela. In the 1930s, Evandro Chagas systematized clinical and diagnostic aspects of chagasic cardiopathy which was eventually presented as a defining characteristic of the disease to the international scientific community by Laranja in the 1950s [16–18]. Since then, numerous studies addressing the clinical aspects, diagnosis, and treatment of CD have been published [19]. It would take over 50 years for most government-sponsored guidelines and manuals to be produced, already in the 21st century, and support public policies for tackling and controlling the disease [20]. Concurrently, during this period, the first randomized prospective clinical trials aiming to answer questions related to the prognosis and treatment of CD emerged [21]. These trials highlighted the pressing need for the identification of biomarkers associated with therapeutic efficacy, based on parasitological, serological, or clinical criteria [22]. Advances in this field have just recently been achieved [23].

The standardization of epidemiological, diagnostic, clinical, and therapeutic data for CD is essential for reference institutions that treat patients with *T*. *cruzi* infection and for research groups that follow cohorts in cross-sectional and longitudinal studies. Large multicenter databases are fundamental for medical research as they enable scientists to analyze significant volumes of specific and uniform clinical information to test hypotheses, assess treatment efficacy, potentially predict progression, and identify epidemiological trends. A clinical database for research is an organized and structured collection of information and data related to patients and their medical conditions, collected with the purpose of conducting clinical research or epidemiological studies. There are different types of clinical databases, including clinical research databases that collect data specifically for research purposes, patient registry databases that record patient information in a routine clinical setting, and epidemiological surveillance databases that monitor the spread of diseases [24].

The Ibero-American Network “Nuevas Herramientas para el Diagnóstico y la Evaluación del Paciente con Enfermedad de Chagas” (NHEPACHA) was created by the initiative of a group of CD researchers from Latin American countries and Spain in March 2012. At present, it comprises 12 research groups from 9 countries. Its aims are: (i) to establish a research, development, and innovation framework for identifying potential new drugs and biomarkers to assist in the diagnosis, prognosis, and management of CD, as well as in the evaluation of new drugs in future clinical trials; (ii) promote the exchange of knowledge; and (iii) planning operational studies and multicenter clinical trials to test new tools and improve existing ones [25].

Aligned with this goal, NHEPACHA now provides a clinical questionnaire (S1 File) and a questionnaire completion manual (S2 File) to the CD clinical research community to facilitate the collection of standardized and comparable data, with the objective of addressing specific research questions, related to the evaluation of treatment efficacy, the identification of risk factors, and the assessment of clinical outcomes. Moreover, this work has been translated to Spanish and Portuguese in order to make it universally accessible and available across endemic areas (S3–S8 Files).

## Materials and methods

### Ethics

The production of this questionnaire did not require the obtention of ethical approval, as patient data was not used in any step of the process. The use of this questionnaire in clinical research must be conducted in accordance with the principles of the Declaration of Helsinki, in agreement with local regulation, and following approval by an independent research ethics committee.

### Methodology

The content of the questionnaire is the result of consensus among experts who are part of the NHEPACHA group. Multiple meetings were conducted with the aim of creating a clinical-epidemiological data collection form that would address important research questions in CD. The information included in the form was organized into the following categories: visit information; institutional information; anonymized patient information; epidemiological information; etiologic diagnosis; clinical presentation; results of diagnostic tests; treatment; and biological samples. Once the minimum and essential items for the questionnaire had been defined, efforts were made to adhere to: (i) current international and national guidelines, including a minimal set of key information; and (ii) established standards that encompassed important aspects such as the privacy and security of patients under ethical evaluation by Institutional Review Boards (IRBs) or research ethics committees, consistent data collection, and data standardization to ensure quality.

The clinical questionnaire finally agreed upon has also been produced in electronic format, using the REDCap platform (Research Electronic Data Capture), electronic data capture software [26,27]. REDCap is a secure, web-based software platform designed to support data capture for research studies, providing: (i) an intuitive interface for validated data capture; (ii) audit trails for tracking data manipulation and export procedures; (iii) automated export procedures for seamless data downloads to common statistical packages; and (iv) procedures for data integration and interoperability with external sources. REDCap is free to nonprofit organizations who join the REDCap Consortium, and it is widely used in the academic research community. Using a centralized REDCap database hosted at ISGlobal will permit the NHEPACHA research centers to use it both independently and collaboratively.

## Results

*I*. *Visit information*: The patient is identified with a code, along with the date of the visit.

*II*. *Institutional information*: Includes the name of the interviewing medical doctor or health professional, the name of the participating institution, its location, and the study’s date and number of ethical/internal review board approval, as well as information participant’s informed consent/assent.

*III*. *Patient information*: Anonymized patient identification, including date of birth and gender.

*IV*. *Epidemiological information*: The patient’s country of origin and that of his/her mother, whether the patient resides in a rural or urban area, how long they resided away from the original endemic area, and whether they have lived in other countries. In the family history section, collected information includes the presence of other relatives having CD and their relationship with the patient; and for female patients, the history of pregnancy, the number of children, and information on whether the children were screened for CD during their first year of life. The most likely transmission mechanism is also registered, as well as comorbidities, co-infections, or any intracardiac device (such as pacemakers), if present.

*V*. *Diagnosis*: Results from serological (commercial name, type, and titers) and parasitological tests are recorded. Similarly, if a molecular test was performed, the specific technique and qualitative or quantitative results are recorded.

*VI*. *Clinical presentation*: The patient’s signs and symptoms are recorded according to the disease stage. For patients in the chronic stage, specific information on their clinical form is recorded: Signs of heart failure and digestive involvement are assessed, and the NYHA classification is used to evaluate the patient’s functional status. Signs associated with congenital infection are also evaluated independently. All data from the physical examination is recorded.

*VII*. *Diagnostic test results*: A checklist to evaluate the presence of remarkable findings from the electrocardiogram, transthoracic echocardiogram, chest X-ray, 24-h Holter monitoring, cardiac magnetic resonance imaging, and BNP or pro-BNP is presented for an objective assessment of cardiac involvement.

*VIII*. *Clinical scales*: Cardiopathy is stratified using preestablished clinical classifications of the disease (“Modified Los Andes,” “Kuschnir,” “Brazilian consensus,” “I Latin American guidelines,” and “American Heart Association Statement”) [5,28–31]. If digestive involvement has been documented, the Rezende classification is used to assess the degree of esophageal involvement [32]. Patients in the acute stage are identified as primary infections or reactivations. The chronic form is characterized and classified as indeterminate, cardiac, digestive, or mixed.

*IX*. *Treatment*: Data on trypanocidal treatment received by the patient is recorded, including the drug used, dosage, and duration of treatment, as well as whether the patient experienced any adverse events and if the treatment was interrupted. Data on the use of cardiovascular medication was included as they may modify prognosis of patients with chronic cardiac form.

*X*. *Biological samples*: In all cases, biological samples were collected from the patient, and information on their type, volume and number of aliquots obtained is collected. Ad hoc guidelines and standard operative procedures (SOPs) for the management of samples in the context of the NHEPACHA network have also been prepared and are presented as a different publication.

## Discussion

A standardized data collection process is essential for obtaining high-quality and comparable data in clinical CD studies, involving participants from different epidemiological settings. A uniform clinical research database is critically important to accelerate breakthroughs in the diagnosis, clinical evolution, and treatment evaluation of this neglected disease, as it can facilitate collaboration between different research teams, streamline data access, accelerate the general pace of research, and inform on regional differences that may result from human and parasite genetic, immunologic, and proteomic characteristics.

The tool presented in this work (in 3 languages: English [S1 and S2 Files], Spanish [S3–S5 Files], and Portuguese [S6–S8 Files]) has the potential to yield a standardized approach for the collection of patient data. This comparable data from centers around the world would enable researchers to evaluate clinical outcomes in a diverse and comprehensive manner, accelerating efforts to close the CD knowledge gap, update existing evidence-based guidelines, and develop new ones.

Importantly, data derived from the NHEPACHA clinical protocol will also allow interested researchers access to annotated patient data, ideal to conduct retrospective studies, generate hypotheses, or guide the development of innovative diagnostic, prognosis, and/or therapeutic tools, while also facilitating tracking a patient’s clinical progression over time. This longitudinal, multicenter, and international perspective is essential, given the chronic nature of CD, and the significant gaps in knowledge regarding the best way to assess its long-term progression and treatment response.

The centralized nature of this database also removes barriers to data acquisition and processing, with researchers from a wide range of institutions being able to quickly access curated clinical data without going through the time-consuming process of collecting it and organizing it. This collaborative approach encourages multidisciplinary research, pooling of expertise, and the ability to tackle complex research questions that require large and diverse datasets.

Researchers can avoid duplicating data collection efforts, as data already stored in the database could be re-used for multiple research projects. This efficiency can contribute to save time and resources. Given its standardized and consensual nature, other researchers also will be able to validate their findings by comparing their results with those of their peers within the NHEPACHA network.

In summary, the homogenization of clinical databases is an essential aspect of modern medical research and will play a pivotal role in advancing the development of new diagnostic and therapeutic tools for Chagas disease. The NHEPACHA Case Report Form (CRF) and its digitalized version housed at REDCap (eCRF) represent a remarkable resource to collect, manage, and share high-quality standardized clinical data. We expect that its use and adoption will enable researchers to address a wide range of issues such as validating diagnostic methods and assessing drug efficacy, helping improve collective understanding of the disease’s complex dynamics.

## Supporting information

S1 File Chagas patient’s questionnaire. (DOCX)

S2 File Questionnaire completion manual. (DOCX)

S3 File A standardized clinical database for research in Chagas disease: NHEPACHA Network (Spanish). (DOCX)

S4 File Chagas patient’s questionnaire (Spanish). (DOCX)

S5 File Questionnaire completion manual (Spanish). (DOCX)

S6 File A standardized clinical database for research in Chagas disease: NHEPACHA Network (Portuguese). (DOCX)

S7 File Chagas patient’s questionnaire (Portuguese). (DOCX)

S8 File Questionnaire completion manual (Portuguese). (DOCX)

S1 Acknowledgments NHEPACHA Network Study Group. (DOCX)
