# Supplementary material for: A standardized clinical database for research in Chagas disease: The NHEPACHA network
Source: PLoS Negl Trop Dis. 2024 Aug 15;18(8):e0012364. doi: 10.1371/journal.pntd.0012364 (PMC11326575; doi:10.1371/journal.pntd.0012364)
Supplement: S1 File — (DOCX) [file pntd.0012364.s001.docx]

| 1. **Information for the current visit** | | | | | | | | | | | | | | | | | | | | | | | | | | | | | | | | | | | | | | | | | | | | | | | | | | | | | | | | | | | | | | | | | | | | | | | | | | | | | | | | | | | | | | |
| --- | --- | --- | --- | --- | --- | --- | --- | --- | --- | --- | --- | --- | --- | --- | --- | --- | --- | --- | --- | --- | --- | --- | --- | --- | --- | --- | --- | --- | --- | --- | --- | --- | --- | --- | --- | --- | --- | --- | --- | --- | --- | --- | --- | --- | --- | --- | --- | --- | --- | --- | --- | --- | --- | --- | --- | --- | --- | --- | --- | --- | --- | --- | --- | --- | --- | --- | --- | --- | --- | --- | --- | --- | --- | --- | --- | --- | --- | --- | --- | --- | --- | --- | --- | --- | --- | --- |
| 1. Patient ID number | | | | | | | | | | | | | | | \|__\|__\|__\| -  Prefix of the NHEPACHA center | | | | | | | | | | | | | | | | | | | | | | | | | | | | | | | | | | | | | | | | | | | ___________________________  Patient’s ID number at the treating institution | | | | | | | | | | | | | | | | | | | | | | | | | | | | |
| 2. Date of visit | | | | | | | | | | | | | | | \|__\|__\| - \|__\|__\|__\| - \|__\|__\|__\|__\| | | | | | | | | | | | | | | | | | | | | | | | | | | | | | | | | | | | | | | | | | | | | | | | | | | | | | | | | | | | | | | | | | | | | | | | |
| 1. **Institutional information** | | | | | | | | | | | | | | | | | | | | | | | | | | | | | | | | | | | | | | | | | | | | | | | | | | | | | | | | | | | | | | | | | | | | | | | | | | | | | | | | | | | | | | |
| 3. Interviewing physician: | | | | | | | | | | | | __________________________________________________________________________ | | | | | | | | | | | | | | | | | | | | | | | | | | | | | | | | | | | | | | | | | | | | | | | | | | | | | | | | | | | | | | | | | | | | | | | | | | |
| 4. Name of institution: | | | | | | | | | | | | | | _________________________________________________________________________ | | | | | | | | | | | | | | | | | | | | | | | | | | | | | | | | | | | | | | | | | | | | | | | | | | | | | | | | | | | | | | | | | | | | | | | | |
| 5. City/location: | | | | | | | | | | | | _____________________ _____________________________________________________ | | | | | | | | | | | | | | | | | | | | | | | | | | | | | | | | | | | | | | | | | | | | | | | | | | | | | | | | | | | | | | | | | | | | | | | | | | |
| 6. Has informed consent been obtained? | | | | | | | | | | | | | | | | | | | | | | | | | | | | | | | | | | | | | | | | | □ Yes | | | | | | | | | | | | | | | | | | | | | | □ No | | | | | | | | | | | | | | | | | | | | | | | |
| 7. Date of ethical approval for the study | | | | | | | | | | | | | | | | | | | | | | | | | | | | | | | | | | | | | | | | | \|__\|__\| - \|__\|__\|__\| - \|__\|__\|__\|__\| | | | | | | | | | | | | | | | | | | | | | | | | | | | | | | | | | | | | | | | | | | | | | |
| 8. Ethical approval’s committee registration number: | | | | | | | | | | | | | | | | | | | | | | | | | | | | | | | | | | | | | | | | | __________________________________________ | | | | | | | | | | | | | | | | | | | | | | | | | | | | | | | | | | | | | | | | | | | | | |
| 1. **Patient information** | | | | | | | | | | | | | | | | | | | | | | | | | | | | | | | | | | | | | | | | | | | | | | | | | | | | | | | | | | | | | | | | | | | | | | | | | | | | | | | | | | | | | | |
| 9. Date of birth | | | | | | | | | | | | | | | | | | | | | | | | | | | | | | | | | | | | | | | | | \|__\|__\| - \|__\|__\|__\| - \|__\|__\|__\|__\| | | | | | | | | | | | | | | | | | | | | | | | | | | | | | | | | | | | | | | | | | | | | | |
| 10. Biological sex | | | | | | | | | | | | | | | | | | | | | | | | | | | | | | | | | | | | | | | | | □ Male | | | | | | | | | | | | | | | | | | | | | | □ Female | | | | | | | | | | | | | | | | | | | | | | | |
| 1. **Epidemiological information** | | | | | | | | | | | | | | | | | | | | | | | | | | | | | | | | | | | | | | | | | | | | | | | | | | | | | | | | | | | | | | | | | | | | | | | | | | | | | | | | | | | | | | |
| 11. Country of origin | | | | | | | | | □ Argentina  □ Belize  □ Bolivia  □ Brazil | | | | | | | | | | | | | | | □ Chile  □ Colombia  □ Costa Rica  □ Ecuador | | | | | | | | | | | | | | | | | | | | | | | □ El Salvador  □ Guatemala  □ Guyana  □ Honduras | | | | | | | | | | | | | | | | | | | | □ Mexico  □ Nicaragua  □ Panamá  □ Paraguay | | | | | | | | | | | | | | □ Peru  □ Suriname  □ Uruguay  □ Venezuela | | | | | |
|  |  |  |  |  |  |  |  |  | □ Other, Specify | | | | | | | | | | | | | | | ___________________________________________________________ | | | | | | | | | | | | | | | | | | | | | | | | | | | | | | | | | | | | | | | | | | | | | | | | | | | | | | | | | | | | | | |
| 12. If different from above, indicate the mother’s county of origin: | | | | | | | | | | | | | | | | | | | | | | | | | | | | | | | | | | | | | | | | | | | | | | | ___________________________________________ | | | | | | | | | | | | | | | | | | | | | | | | | | | | | | | | | | | | | | | |
| 13. Possible transmission mechanism | | | | | | | | | | | | | | | | | □ Unknown  □ Post-transfusion | | | | | | | | | | | | | | | | | | | | | | | | | | | | | | □ Oral  □ Transplant | | | | | | | | | | | | | | | | | | | | | | | | | | | | □ Vector-borne  □ Vertical (Congenital) | | | | | | | | | | | |
|  |  |  |  |  |  |  |  |  |  |  |  |  |  |  |  |  | □ Laboratory accident | | | | | | | | | | | | | | | | | | | | | | | | | | | | | | | | | | | | | | | | | | | | | | | | | | | | | | | | | | | | | | | | | | | | | |
| 14. In case of transfusion, transplant or accident, indicate: | | | | | Location:  _____________________ | | | | | | | | | | | | | | | | | | | | | | | Reason:  _____________________ | | | | | | | | | | | | | | | | | | | | | | | | | | | | | | | | | | | | | Date:  \|__\|__\| - \|__\|__\|__\| - \|__\|__\|__\|__\| | | | | | | | | | | | | | | | | | | | | | |
| 15. The patient lives in: | | | | | | | | | | | | | | | | | | | | | | | | | | | | | | | | | | | | | | | | | □ Rural area | | | | | | | | | | | | | | | | | | | | | | | | □ Urban area | | | | | | | | | | | | | | | | | | | | | |
| 16. Time living outside of an endemic area: | | | | | | | | | | | | | | | | | | | | | | | □ Still lives in endemic area | | | | | | | | | | | | | | | | | | | | | | | | | | | | | | | □ < 10 years | | | | | | | | | | | | | | | | | | | | | | | | | | □ > 10 years | | | | | | |
| 17. Other places where the patient has lived: | | | | | | | | | | ____________________________________________________________________________ | | | | | | | | | | | | | | | | | | | | | | | | | | | | | | | | | | | | | | | | | | | | | | | | | | | | | | | | | | | | | | | | | | | | | | | | | | | | |
| 18. Family history of Chagas disease? | | | | | | | | | | | | | | | □ Mother  □ Father  □ Other, specify: ____________________________ | | | | | | | | | | | | | | | | | | | | | | | | | | | | | | | | | | | | | | | | | | | □ Siblings  □ Children | | | | | | | | | | | | | | | | | | | | | | | | | | | | |
| If the patient is female, please answer questions 19-21, otherwise, jump to 22. | | | | | | | | | | | | | | | | | | | | | | | | | | | | | | | | | | | | | | | | | | | | | | | | | | | | | | | | | | | | | | | | | | | | | | | | | | | | | | | | | | | | | | |
| 19. Is the patient pregnant?  20. Does the patient have children?  21. Were the children tested for Chagas disease at 8, 10 or 12 months after birth? | | | | | | | | | | | | | | | □ Yes  □ Yes    □ Yes | | | | | | | | | | | | | | | | | | | | | | | | | | | | | | | □ No  □ No    □ No | | | | | | | | | | | | | □ Unknown  □ Unknown | | | | | | | | | | | | | | | | | □ Ignored  □ Ignored | | | | | | | | | | |
| 22. Does the patient present any of the following comorbidities? | | | | | | | | | | | | | | | □ No information  □ None  □ Diabetes Mellitus  □ Hypothyroidism  □ Systemic hypertension  □ Other, specify | | | | | | | | | | | | | | | | | | | | | | | | | | | | | | | □ Non-Chagasic cardiomyopathy  □ Liver disease  □ Dyslipidaemia  □ Neoplasia  □ Neuropsychiatric disorders  _______________________ | | | | | | | | | | | | | | | | | | | | | | | | | | | | | | □ Auto-immune disease  □ COPD  □ Asthma  □ History of thrombo-embolic disease | | | | | | | | | | |
| 23. Does the patient present any co-infection? | | | | | | | | | | | | | | | □ No information  □ Other, specify | | | | | | | | | | | | | | | | | | | | | | | | | | | | | | | □ HIV  _______________________ | | | | | | | | | | | | | | | | | | | | | | | | | | | | | | □ Covid19 | | | | | | | | | | |
| 24. Does the patient have an intracardiac device? | | | | | | | | | | | | | | | □ No | | | | | | | | | | | | | | | | | | | | | | | | | | | | | | | □ Single-chamber pacemaker | | | | | | | | | | | | | | | | | | | | | | | | | | | | | | □ Dual-chamber pacemaker | | | | | | | | | | |
|  | | | | | | | | | | | | | | | □ ICD | | | | | | | | | | | | | | | | | | | | | | | | | | | | | | | □ CRT-P | | | | | | | | | | | | | | | | | | | | | | | | | | | | | | □ CRT-D | | | | | | | | | | |
| 1. **Etiological diagnosis** | | | | | | | | | | | | | | | | | | | | | | | | | | | | | | | | | | | | | | | | | | | | | | | | | | | | | | | | | | | | | | | | | | | | | | | | | | | | | | | | | | | | | | |
| 25. Has a parasitological test been performed? | | | | | | | | | | | | | | | □ Yes  □ No | | | | | | | | | | | | | | | | | | 25.1 If yes, which one?   \| □ Strout  □ Micromethod \| \| --- \| \| □ Xenodiagnosis  □ Other, which one? _____________________ \| | | | | | | | | | | | | | | | | | | | | | | | | | | | | | | | | | | | | | | | | | | | | | | | | | | | | | |
| 25.2 Date of parasitological test: | | | | | | | | | | | | | | | | | | | | | | | | | | | | | | | | | \|__\|__\| - \|__\|__\|__\| - \|__\|__\|__\|__\| | | | | | | | | | | | | | | | | | | | | | | | | | | | | | | | | | | | | | | | | | | | | | | | | | | | | | |
| 25.3 Result | | | | | | | | | | | | | | | □ Not detectable | | | | | | | | | | | | | | | | | | | | | | | | | | | | | | | | | | | | | | | | | | |  | | | | | | | | | | | | | | | | | | | | | | | | | | | | |
|  | | | | | | | | | | | | | | | □ Detectable | | | | | | | | | | | | | | | | | | | Quantitative value, if available_____________________ | | | | | | | | | | | | | | | | | | | | | | | | | | | | | | | | | | | | | | | | | | | | | | | | | | | | |
| 26. Was serologic test number 1 performed? | | | | | | | | | | | | | | | □ Yes  □ No | | | | | | | | | | | | | | | | | | 26.1 If yes, which assay?  □ ELISA Ig M  □ ELISA Ig G  □ RDT  □ IHA  □ IIF  □ CMIA  □ Other, specify_______________________ | | | | | | | | | | | | | | | | | | | | | | | | | | | | | | | | | | | | | | | | | | | | | | | | | | | | | |
| 26.2 Date of serologic test number 1: | | | | | | | | | | | | | | | | | | | | | | | | | | | | | | | | | | | | | | \|__\|__\| - \|__\|__\|__\| - \|__\|__\|__\|__\| | | | | | | | | | | | | | | | | | | | | | | | | | | | | | | | | | | | | | | | | | | | | | | | | |
| 26.3 Result | | | | | | | | | | □ Not detectable | | | | | | | | | | | | | | | | □ Detectable | | | | | | | | | | | | | | | | | | | | | | | | | | | | | | | | Commercial name______________________  Quantitative result, if available_____________________  Cut-off value_______________________ | | | | | | | | | | | | | | | | | | | | | | | | | | | | |
|  | | | | | | | | | | | | | | |  | | | | | | | | | | | | | | | | | | | | | | | | | | | | | | | | | | | | | | | | | | |  | | | | | | | | | | | | | | | | | | | | | | | | | | | | |
|  | | | | | | | | | | | | | | |  | | | | | | | | | | | | | | | | | | | | | | | | | | | | | | | | | | | | | | | | | | |  | | | | | | | | | | | | | | | | | | | | | | | | | | | | |
| 27. Was serologic test number 2 performed? | | | | | | | | | | | | | | | □ Yes  □ No | | | | | | | | | | | | | | | | | | | | | | | | | | | | | | | | | | | | | | | | | | | 27.1 If yes, which assay?  □ ELISA Ig M  □ ELISA Ig G  □ RDT  □ IHA  □ IIF  □ CMIA  □ Other, specify? _______________________ | | | | | | | | | | | | | | | | | | | | | | | | | | | | |
| 27.2 Date of serologic test number 2? | | | | | | | | | | | | | | | \|__\|__\| - \|__\|__\|__\| - \|__\|__\|__\|__\| | | | | | | | | | | | | | | | | | | | | | | | | | | | | | | | | | | | | | | | | | | |  | | | | | | | | | | | | | | | | | | | | | | | | | | | | |
| 27.3 Result | | | | | | | | □ Not detectable | | | | | | | | | | | | | | | | | | | | | □ Detectable | | | | | | | | | | | | | | | | | | | | | | | | | | | | | Commercial name______________________  Quantitative result, if available_____________________  Cut-off value_______________________ | | | | | | | | | | | | | | | | | | | | | | | | | | | | |
| 28. Was serologic test number 3 performed? | | | | | | | | | | | | | | | □ Yes  □ No | | | | | | | | | | | | | | | | | | | | | | | | | | | | | | | | | | | | | | | | | | | 28.1 If yes, which assay?  □ ELISA Ig M  □ ELISA Ig G  □ RDT  □ IHA  □ IIF  □ CMIA  □ Other, specify? _______________________ | | | | | | | | | | | | | | | | | | | | | | | | | | | | |
| 28.2 Date of serologic test number 3? | | | | | | | | | | | | | | | \|__\|__\| - \|__\|__\|__\| - \|__\|__\|__\|__\| | | | | | | | | | | | | | | | | | | | | | | | | | | | | | | | | | | | | | | | | | | |  | | | | | | | | | | | | | | | | | | | | | | | | | | | | |
| 28.3 Result | | | | | | | | □ Not detectable | | | | | | | | | | | | | | | | | | □ Detectable | | | | | | | | | | | | | | | | | | | | | | | | | | | | | | | | Commercial name______________________  Quantitative result, if available_____________________  Cut-off value_______________________ | | | | | | | | | | | | | | | | | | | | | | | | | | | | |
| 29. Was molecular test 1 performed? | | | | | | | | | | | | | | | □ Yes  □ No | | | | | | | | | | | | | | | | | | | | | | | | | | | 29.1 If yes, which assay?  □ Conventional PCR  □ qPCR  □ LAMP  □ Other, specify? _____________________________________ | | | | | | | | | | | | | | | | | | | | | | | | | | | | | | | | | | | | | | | | | | | | |
| 29.2 Date of molecular test 1 | | | | | | | | | | | | | | | | | | | | | | | | | | | | | | | | | | | | | | \|__\|__\| - \|__\|__\|__\| - \|__\|__\|__\|__\| | | | | | | | | | | | | | | | | | | | | | | | | | | | | | | | | | | | | | | | | | | | | | | | | |
| 29.3 Result | | | | | | | | | | | | | | | □ Not detectable | | | | | | | | | | | | | | | | | | | | | | | | | | | | | | | | | | | | | | | | | | | □ Detectable  Quantitative value, if available_____________________ | | | | | | | | | | | | | | | | | | | | | | | | | | | | |
| 30. Was molecular test 2 performed? | | | | | | | | | | | | | | | □ Yes  □ No | | | | | | | | | | | | | | | | | | | | | | | | | | | | | | | | | | | | | | | | | | | 30.1 If yes, which assay?  □ Conventional PCR  □ qPCR  □ LAMP  □ Other, specify? _____________________________________ | | | | | | | | | | | | | | | | | | | | | | | | | | | | |
| 30.2 Date of molecular test 2: | | | | | | | | | | | | | | | | | | | | | \|__\|__\| - \|__\|__\|__\| - \|__\|__\|__\|__\| | | | | | | | | | | | | | | | | | | | | | | | | | | | | | | | | | | | | | | | | | | | | | | | | | |  | | | | | | | | | | | | | | | |
| 30.3 Result | | | | | | | | | | | | | | | □ Not detectable | | | | | | | | | | | | | | | | | | | | | | | | | | | | | | | | | | | | | | | | | | | □ Detectable  Quantitative value, if available____________________ | | | | | | | | | | | | | | | | | | | | | | | | | | | | |
| 1. **Clinical presentation symptoms** | | | | | | | | | | | | | | | | | | | | | | | | | | | | | | | | | | | | | | | | | | | | | | | | | | | | | | | | | | | | | | | | | | | | | | | | | | | | | | | | | | | | | | |
| 31. Acute | | □ Ignored  □ None   \| □ Chagoma \| \| --- \| \| □ Myalgia \| \| □ Arthralgia \| \| □ Splenomegaly \| \| □ Asthenia \| \| □ Dyspnoea  □ Headache  □ Other, specify \| | | | | | | | | | | | | | | | | | | | | | | | | | | | | | \| □ Romaña’s sign \| \| --- \| \| □ Abdominal pain \| \| □ Shivers \| \| □ Hepatomegaly \| \| □ Adynamia \| \| \| □ Thoracic pain  □ Lymphadenopathy  □ Tachycardia \| \| | | | | | | | | | | | | | | | | | | | | | | | | | | | | | | | | | | | | | | \| □ Fever \| \| \| --- \| --- \| \| □ Facial oedema \| \| \| □ Lower limb oedema \| \| \| \| □ Jaundice \| \| \| □ Prostration \| \| □ Skin nodules in lower limbs \| \| \| | | | | | | | | | | | | | | | | | |
| 32. Chronic cardiovascular | | □ Ignored  □ None   \| □ Dizziness  □ Syncope \| \| --- \|   □ Palpitations  □ Peripheral oedema  □ Wheezing  □ Other, specify | | | | | | | | | | | | | | | | | | | | | | | | | | | | | NYHA Classification:   \| □ Class I \| \| --- \| \| □ Class II  □ Class III  □ Class IV \| \| □ Fatigue \| | | | | | | | | | | | | | | | | | | | | | | | | | | | | | | | | | | | | | | □ Abdominal swelling  □ Fast weight gain  □ Shortness of breath  □ Chest pain  Thrombo-embolic event   \| □ Yes \| \| --- \| \| □ No  □ Unknown \| | | | | | | | | | | | | | | | | | |
| 33. Chronic digestive | | □ Ignored  □ None  □ Dysphagia  □ Diarrhoea  □ Other, specify | | | | | | | | | | | | | | | | | | | | | | | | | | | | | □ Odynophagia  □ Regurgitation | | | | | | | | | | | | | | | | | | | | | | | | | | | | | | | | | | | | | | Constipation   \| □ Yes  □ <7days □>7days \| \| --- \| \| □ No \| | | | | | | | | | | | | | | | | | |
| 34. Vertical transmission (congenital) | | □ Ignored  □ None   \| □ Splenomegaly  □ Lymphadenopathy  □ Tachycardia  □ Cyanosis  □ Other, specify? \| \| --- \| | | | | | | | | | | | | | | | | | | | | | | | | | | | | | \| □ Low weight at birth \| \| --- \| \| □ Prematurity \| \| □ Polypnea \| \| □ Irritability/Apathy  □ Jaundice  □ Seizures \| | | | | | | | | | | | | | | | | | | | | | | | | | | | | | | | | | | | | | | □ Hepatomegaly   \| □ Fever \| \| --- \| \| □ Generalized oedema  □ Bradycardia  □ Microcephaly \| \|  \| | | | | | | | | | | | | | | | | | |
|  | | | | | | | | | | | | | | | | | | | | | | | | | | | | | | | | | | | | | | | | | | | | | | | | | | | | | | | | | | | | | | | | | | | | | | | | | | | | | | | | | | | | | | |
| **Signs/Physical exam** | | | | | | | | | | | | | | | | | | | | | | | | | | | | | | | | | | | | | | | | | | | | | | | | | | | | | | | | | | | | | | | | | | | | | | | | | | | | | | | | | | | | | | |
| 35. Weight (kilograms) | | | \|__\|__\|__\|.\|__\|__\| (kg) | | | | | | | | | | | | | | | | | | | | | | | | | | | | | | | | | | | 36. Height (meters) | | | | | | | | | | | | | | | | | | | | | | | | | | | | | | | | | \|__\|.\|__\|__\| (m) | | | | | | | | | | | | | | | |
| 37. Vital signs | | | □ Ignored  37.1 Blood pressure | | | | | | | | | | | | | | | | | | | | | | □ Measured  \|__\|__\|__/__\|__\|__\| | | | | | | | | | | | | | | | | | | | | | | | | | 37.2 Temperature (ºC) | | | | | | | | | | | | | | | | | | | | | | | | | | | | \|__\|__\|__\| | | | | | | | | |
|  |  |  | 37.3 Breathing rate | | | | | | | | | | | | | | | | | | | | | | | | | | | | | | \|__\|__\|__\| | | | | | | | | | | | | | | | | | 37.4 Heart rate | | | | | | | | | | | | | | | | | | | | | | | | | | | | | | | \|__\|__\|__\| | | | | | |
|  |  |  | 37.5 Oxygen saturation | | | | | | | | | | | | | | | | | | | | | | | | | | | | | | | | | | | \|__\|__\|_%_\| | | | | | | | | | | | | | | | | | | | | | | | | | | | | | | | | |  | | | | | | | | | | | | | | | |
| 38. Signs of heart failure | | | □ Ignored  □ None   \| □ Lower limb oedema \| \| --- \| \| □ Tachycardia  □ Rales  □ Other, which one? \| | | | | | | | | | | | | | | | | | | | | | | | | | | | | | | | | | | | □ Jugular vein distention  □ Hepatomegaly  □ S3-gallop | | | | | | | | | | | | | | | | | | | | | | | | | | | | | | | | | □ Displaced apex beat  □ Irregular pulse | | | | | | | | | | | | | | | |
| 1. **Diagnostic test results** | | | | | | | | | | | | | | | | | | | | | | | | | | | | | | | | | | | | | | | | | | | | | | | | | | | | | | | | | | | | | | | | | | | | | | | | | | | | | | | | | | | | | | |
| 39. Electrocardiogram | | | | | | | | | | | | | | | □ Not performed  □ Changes suggestive of Chagasic cardiomyopathy | | | | | | | | | | | | | | | | | | | | | | | | | | | | | | | | | | | | | | | | | | | □ No alterations  □ Unspecific alterations | | | | | | | | | | | | | | | | | | | | | | | | | | | | |
| 39.1 Electrocardiogram date | | | | | | | | | | | | | | | | | | | | | | | | \|__\|__\| - \|__\|__\|__\| - \|__\|__\|__\|__\| | | | | | | | | | | | | | | | | | | | | | | | | | | | | | | | | | | | | | | | | | | | | | | | | | | | | | | | | | | | | | | |
| 39.2 The ECG recorded is in pacemaker rhythm | | | | | | | | | | | | | | | | | | | | | | | | | | | | | | | | | | | | □ Yes | | | | | | | | | | | | | | | | | | | | | | | | | | | | □ No | | | | | | | | | | | | | | | | | | | | | | |
| 39.2.1 If yes, please specify: | | | | | | | | | | | | | | | | | | | | | | | | | | | | | | | | | | | | □ Single-chamber pacemaker | | | | | | | | | | | | | | | | | | | | | | | | | | | | □ Dual-chamber pacemaker | | | | | | | | | | | | | | | | | | | | | | |
|  | | | | | | | | | | | | | □ ICD | | | | | | | | | | | | | | | | | | | | | | | | | | | | | | | | | | | □ CRT-P | | | | | | | | | | | | | | | | | | | | | | | | | | □ CRT-D | | | | | | | | | | | | |
| 40. If changes suggestive of chagasic cardiomyopathy: | | | 40.1 Right branch bundle block | | | | | | | | | | | | | | | | | | | | | | | | | | | | | | | | | | | | | | | | | | | | | | | | | | | | | | | | | | | | | | | | | | | | | | | | | | | | | | | □ Yes □ No | | | | |
|  |  |  | 40.2 Left branch bundle block | | | | | | | | | | | | | | | | | | | | | | | | | | | | | | | | | | | | | | | | | | | | | | | | | | | | | | | | | | | | | | | | | | | | | | | | | | | | | | | □ Yes □ No | | | | |
|  |  |  | 40.3 Left anterior fascicular block | | | | | | | | | | | | | | | | | | | | | | | | | | | | | | | | | | | | | | | | | | | | | | | | | | | | | | | | | | | | | | | | | | | | | | | | | | | | | | | □ Yes □ No | | | | |
|  |  |  | 40.4 Frequent ventricular extrasystoles (more than 1) | | | | | | | | | | | | | | | | | | | | | | | | | | | | | | | | | | | | | | | | | | | | | | | | | | | | | | | | | | | | | | | | | | | | | | | | | | | | | | | □ Yes □ No | | | | |
|  |  |  | 40.5 Electrically inactive area (Q wave ≥ 40 ms and/or Q wave depth ≥ 25% of the R-wave in two contiguous leads) | | | | | | | | | | | | | | | | | | | | | | | | | | | | | | | | | | | | | | | | | | | | | | | | | | | | | | | | | | | | | | | | | | | | | | | | | | | | | | | □ Yes □ No | | | | |
|  |  |  | 40.6 Second-degree AV block | | | | | | | | | | | | | | | | | | | | | | | | | | | | | | | | | | | | | | | | | | | | | | | | | | | | | | | | | | | | | | | | | | | | | | | | | | | | | | | □ Yes □ No | | | | |
|  |  |  | 40.7 Third-degree AV block | | | | | | | | | | | | | | | | | | | | | | | | | | | | | | | | | | | | | | | | | | | | | | | | | | | | | | | | | | | | | | | | | | | | | | | | | | | | | | | □ Yes □ No | | | | |
|  |  |  | 40.8 Ventricular tachycardia (sustained or not) | | | | | | | | | | | | | | | | | | | | | | | | | | | | | | | | | | | | | | | | | | | | | | | | | | | | | | | | | | | | | | | | | | | | | | | | | | | | | | | □ Yes □ No | | | | |
|  |  |  | 40.9 Atrial tachycardia and/or atrial fibrillation or flutter | | | | | | | | | | | | | | | | | | | | | | | | | | | | | | | | | | | | | | | | | | | | | | | | | | | | | | | | | | | | | | | | | | | | | | | | | | | | | | | □ Yes □ No | | | | |
|  |  |  | 40.10 Sinus bradycardia (<50 bpm) | | | | | | | | | | | | | | | | | | | | | | | | | | | | | | | | | | | | | | | | | | | | | | | | | | | | | | | | | | | | | | | | | | | | | | | | | | | | | | | □ Yes □ No | | | | |
|  |  |  | 40.11 Primary T wave changes | | | | | | | | | | | | | | | | | | | | | | | | | | | | | | | | | | | | | | | | | | | | | | | | | | | | | | | | | | | | | | | | | | | | | | | | | | | | | | | □ Yes □ No | | | | |
|  |  |  | 40.12 Pacemaker rhythm | | | | | | | | | | | | | | | | | | | | | | | | | | | | | | | | | | | | | | | | | | | | | | | | | | | | | | | | | | | | | | | | | | | | | | | | | | | | | | |  | | | | |
|  |  |  | 40.13 Other, describe________________________________________________ | | | | | | | | | | | | | | | | | | | | | | | | | | | | | | | | | | | | | | | | | | | | | | | | | | | | | | | | | | | | | | | | | | | | | | | | | | | | | | | □ Yes □ No | | | | |
| 41. Echocardiogram | | | □ Not performed | | | | | | | | | | | | | | | | | | | | | | | | | | | □ No alterations | | | | | | | | | | | | | | | | | | | | | | | | | □ Abnormal | | | | | | | | | | | | | | | | | | | | | | □ No Chagas alterations | | | | | | | | | |
| 41.1 Echocardiogram date | | | | | | | | | | | | | | | | | | | | | | | | \|__\|__\| - \|__\|__\|__\| - \|__\|__\|__\|__\| | | | | | | | | | | | | | | | | | | | | | | | | | | | | | | | | | | | | | | | | | | | | | | | | | | | | | | | | | | | | | | |
| 42. Echocardiogram data | | | | | | | | | | | | | | | 42.1 LVEF: \|__\|__\| % | | | | | | | | | | | | | | | | | | | | | | | | | | | | | | | | | | Method: | | | | | | | | | | | | | | | | | | □ Simpson | | | | | | | | | | | | | | □ Teicholtz | | | | | |
|  | | | | | | | | | | | | | | | 42.2 Wall motion changes | | | | | | | | | | | | | | | | | | | | | | | | | | | | | | | | | | □ Yes | | | | | | | | | | | | | | | | | | □ No | | | | | | | | | | | | | | □ Ignored | | | | | |
|  |  |  |  |  |  |  |  |  |  |  |  |  |  |  | 42.3 LV dilation | | | | | | | | | | | | | | | | | | | | | | | | | | | | | | | | | | □ Yes | | | | | | | | | | | | | | | | | | □ No | | | | | | | | | | | | | | □ Ignored | | | | | |
|  |  |  |  |  |  |  |  |  |  |  |  |  |  |  | 42.4 LV systolic function | | | | | | | | | | | | | | | | | | | | | | | | | | | | | | | | | | □ Normal | | | | | | | | | | | | | | | | | | □ Abnormal | | | | | | | | | | | | | | □ Ignored | | | | | |
|  |  |  |  |  |  |  |  |  |  |  |  |  |  |  | 42.4.1 If abnormal: | | | | | | | | | | | | | | | | | | | | | | | | | | | | | | | | | | □ Mild | | | | | | | | | | | | | | | | | | □ Moderate | | | | | | | | | | | | | | □ Severe | | | | | |
|  |  |  |  |  |  |  |  |  |  |  |  |  |  |  | 42.5 RV systolic function | | | | | | | | | | | | | | | | | | | | | | | | | | | | | | | | | | □ Normal | | | | | | | | | | | | | | | | | | □ Abnormal | | | | | | | | | | | | | | □ Ignored | | | | | |
|  |  |  |  |  |  |  |  |  |  |  |  |  |  |  | 42.6 Apical aneurysm? | | | | | | | | | | | | | | | | | | | | | | | | | | | | | | | | | | □ Yes | | | | | | | | | | | | | | | | | | □ No | | | | | | | | | | | | | | □ Ignored | | | | | |
|  |  |  |  |  |  |  |  |  |  |  |  |  |  |  | 42.7 Left ventricular thrombus | | | | | | | | | | | | | | | | | | | | | | | | | | | | | | | | | | □ Yes | | | | | | | | | | | | | | | | | | □ No | | | | | | | | | | | | | | □ Ignored | | | | | |
|  |  |  |  |  |  |  |  |  |  |  |  |  |  |  | 42.8 Valvular heart disease | | | | | | | | | | | | | | | | | | | | | | | | | | | | | | | | | | □ Yes | | | | | | | | | | | | | | | | | | □ No | | | | | | | | | | | | | | □ Ignored | | | | | |
|  |  |  |  |  |  |  |  |  |  |  |  |  |  |  | 42.8.1 If present, are they related to Chagas disease? | | | | | | | | | | | | | | | | | | | | | | | | | | | | | | | | | | | | | | | | | | | | | | | | | | | | □ Yes | | | | | | | | | | | | | | □ No | | | | | |
|  |  |  |  |  |  |  |  |  |  |  |  |  |  |  | 42.8.2 Please specify | | | | | | | | | | | | | | | | | | | | | | | | | | | | | | | | | | □ Mitral regurgitation □ Tricuspid regurgitation | | | | | | | | | | | | | | | | | | | | | | | | | | | | | | | | | | | | | |
|  |  |  |  |  |  |  |  |  |  |  |  |  |  |  | 42.8.3 Degree of valvular insufficiency | | | | | | | | | | | | | | | | | | | | | | | | | | | | | | | | | | □ Mild | | | | | | | | | | | | | | | | | | □ Moderate | | | | | | | | | | | | | | □ Severe | | | | | |
|  |  |  |  |  |  |  |  |  |  |  |  |  |  |  | 42.9 Diastolic dysfunction | | | | | | | | | | | | | | | | | | | | | | | | | | | | | | | | | | □ Yes | | | | | | | | | | | | | | | | | | □ No | | | | | | | | | | | | | | □ Ignored | | | | | |
|  |  |  |  |  |  |  |  |  |  |  |  |  |  |  | 42.9.1 If diastolic dysfunction is present | | | | | | | | | | | | | | | | | | | | | | | | | | | | | | | | | | □ Type I | | | | | | | | | | | | | | | | | | □ Type II | | | | | | | | | | | | | | □ Type III | | | | | |
|  |  |  |  |  |  |  |  |  |  |  |  |  |  |  | 42.10 E/E’ ratio \|__\|__\|.\|\|__\| | | | | | | | | | | | | | | | | | | | | | | | | | | | | | | | | | |  | | | | | | | | | | | | | | | | | |  | | | | | | | | | | | | | |  | | | | | |
|  |  |  |  |  |  |  |  |  |  |  |  |  |  |  | 42.11 Left atrial volume | | | | | | | | | | | | | | | | | | | | | | | | | | | | | | | | | | \|__\|__\|.\|\|__\|mL/m^2^ | | | | | | | | | | | | | | | | | |  | | | | | | | | | | | | | |  | | | | | |
|  |  |  |  |  |  |  |  |  |  |  |  |  |  |  | 42.12 Pulmonary hypertension | | | | | | | | | | | | | | | | | | | | | | | | | | | | | | | | | | □ Yes | | | | | | | | | | | | | | | | | | □ No | | | | | | | | | | | | | | \|__\|__\|__\|mmHg | | | | | |
|  |  |  |  |  |  |  |  |  |  |  |  |  |  |  | 42.13 Other findings | | | | | | | | | | | | | | | | | | | | | | | | | | | | | | | | | | □ Yes | | | | | | | | | | | | | | | | | | □ No | | | | | | | | | | | | | |  | | | | | |
|  |  |  |  |  |  |  |  |  |  |  |  |  |  |  | 42.13.1 If yes, specify: | | | | | | | | | | | | | | | | | | | | | | | | | | | | | | | | | |  | | | | | | | | | | | | | | | | | | | | | | | | | | | | | | | | | | | | | |
| 43. Chest X-ray | | | | | | | | | | | | | | | □ Not performed | | | | | | | | | | | | | | | | | | | | | | | | | | | | | | | | | | | | | | | | | | | □ Performed | | | | | | | | | | | | | | | | | | | | | | | | | | | | |
| 43.1Chest x-ray date | | | | | | | | | | | | | | | | | | | | | | | | \|__\|__\| - \|__\|__\|__\| - \|__\|__\|__\|__\| | | | | | | | | | | | | | | | | | | | | | | | | | | | | | | | | | | | | | | | | | | | | | | | | | | | | | | | | | | | | | | |
| 43.2. Cardiomegaly? | | | | | | | | | | | | | | | □ No  □ Yes  Cardiothoracic index_________________ | | | | | | | | | | | | | | | | | | | | | | | | | | | | | | | | | | | | | | | | | | | | | | | | | | | | | | | | | | | | | | | | | | | | | | | |
| 44. 24 hour Holter monitoring | | | | | | | | | | | | | | | | | | | | □ Not performed | | | | | | | | | | | | | | | | | | | | | | | | | | | | | | | | | | | | | | | | | | | | | | | | □ Performed | | | | | | | | | | | | | | | | | | |
| 44.1 Holter date | | | | | | | | | | | | | | | | | | | | \|__\|__\| - \|__\|__\|__\| - \|__\|__\|__\|__\| | | | | | | | | | | | | | | | | | | | | | | | | | | | | | | | | | | | | | | | | | | | | | | | | | | | | | | | | | | | | | | | | | | |
|  | | | | | | | | | | | | | 44.1.1 Sustained ventricular tachycardia | | | | | | | | | | | | | | | | | | | | | | | | | | | | | | | | | | | | | | | | | | | | | □ Yes | | | | | | | | | | | | | | | □ No | | | | | | | | | | | | | □ Ignored |
|  | | | | | | | | | | | | | 44.1.2 Non-sustained ventricular tachycardia | | | | | | | | | | | | | | | | | | | | | | | | | | | | | | | | | | | | | | | | | | | | | □ Yes | | | | | | | | | | | | | | | □ No | | | | | | | | | | | | | □ Ignored |
|  | | | | | | | | | | | | | 44.1.3 Atrial fibrillation | | | | | | | | | | | | | | | | | | | | | | | | | | | | | | | | | | | | | | | | | | | | | □ Yes | | | | | | | | | | | | | | | □ No | | | | | | | | | | | | | □ Ignored |
|  | | | | | | | | | | | | | 44.1.4 Bradycardia <40 bpm | | | | | | | | | | | | | | | | | | | | | | | | | | | | | | | | | | | | | | | | | | | | | □ Yes | | | | | | | | | | | | | | | □ No | | | | | | | | | | | | | □ Ignored |
|  | | | | | | | | | | | | | 44.1.5 Sinus pause > 3 seconds | | | | | | | | | | | | | | | | | | | | | | | | | | | | | | | | | | | | | | | | | | | | | □ Yes | | | | | | | | | | | | | | | □ No | | | | | | | | | | | | | □ Ignored |
| 45. Cardiac MRI | | | | | | | | | | | | | | | | | | | | □ Not performed | | | | | | | | | | | | | | | | | | | | | | | | | | | | | | | | | | | | | | | | | | | | | | | | □ Performed | | | | | | | | | | | | | | | | | | |
| 45.1 Cardiac MRI date | | | | | | | | | | | | | | | | | | | | \|__\|__\| - \|__\|__\|__\| - \|__\|__\|__\|__\| | | | | | | | | | | | | | | | | | | | | | | | | | | | | | | | | | | | | | | | | | | | | | | | | | | | | | | | | | | | | | | | | | | |
|  | | | | | | | 45.1.1 Cardiac fibrosis | | | | | | | | | | | | | | | | | | | | | | | | | | | | | | | | | □ Yes | | | | | | | | | | | | | | | | | | | | | □ No | | | | | | | | | | | | | | | | | | □ Ignored | | | | | | | |
|  | | | | | | | 45.1.2 Cardiac fibrosis mass | | | | | | | | | | | | | | | | | | | | | | | | | | | | | | | | | \|__\|__\|__\| (g) | | | | | | | | | | | | | | | | | | | | | | | | | | | | | | | | | | | | | | | | | | | | | | |
|  | | | | | | | 45.1.3 LV aneurysm | | | | | | | | | | | | | | | | | | | | | | | | | | | | | | | | | □ Yes | | | | | | | | | | | | | | | | | | | | | □ No | | | | | | | | | | | | | | | | | | □ Ignored | | | | | | | |
|  | | | | | | | 45.1.4 LV thombus | | | | | | | | | | | | | | | | | | | | | | | | | | | | | | | | | □ Yes | | | | | | | | | | | | | | | | | | | | | □ No | | | | | | | | | | | | | | | | | | □ Ignored | | | | | | | |
| 46. BNP and/or NT-proBNP | | | | | | | | | | | | | | | | | | | | □ Not performed | | | | | | | | | | | | | | | | | | | | | | | | | | | | | | | | | | | | | | | | | | | | | | | | □ Performed | | | | | | | | | | | | | | | | | | |
| 46. 1 BNP and/or NT-proBNP date | | | | | | | | | | | | | | | | | | | | \|__\|__\| - \|__\|__\|__\| - \|__\|__\|__\|__\| | | | | | | | | | | | | | | | | | | | | | | | | | | | | | | | | | | | | | | | | | | | | | | | | | | | | | | | | | | | | | | | | | | |
|  | | | | | | | 46.1.1 BNP | | | | | | | | | | | | | | | | | | | | | | | | | □ Normal | | | | | | | | | | | | | | | | | | | □ Abnormal | | | | | | | | | | | | | | | \|__\|__\|__\|__\| (pg/mL) | | | | | | | | | | | | | | | | | | □ Ignored | | |
|  | | | | | | | 46.1.2 NT-proBNP | | | | | | | | | | | | | | | | | | | | | | | | | □ Normal | | | | | | | | | | | | | | | | | | | □ Abnormal | | | | | | | | | | | | | | | \|__\|__\|__\|__\| (pg/mL) | | | | | | | | | | | | | | | | | | □ Ignored | | |
| 1. **Classifications** | | | | | | | | | | | | | | | | | | | | | | | | | | | | | | | | | | | | | | | | | | | | | | | | | | | | | | | | | | | | | | | | | | | | | | | | | | | | | | | | | | | | | | |
| 47. Kuschnir classification | | | | | | | | | | | | | | | | | |  | | | | | | | | | □ 0 | | | | | | | | | | | | | | | | | | □ 1 | | | | | | | | | | | □ 2 | | | | | | | | | | | | | | | | □ 3 | | | | | | | | | | | | | □ Ignored | |
| 48. Brazilian consensus classification | | | | | | | | | | | | | | | | | | □ FI | | | | | | | | | □ A | | | | | | | | | | | | | | | | | | □ B1 | | | | | | | | | | | □ B2 | | | | | | | | | | | | | | | | □ C  □ D | | | | | | | | | | | | | □ Ignored | |
| 49. Latin American classification | | | | | | | | | | | | | | | | | | □ A | | | | | | | | | □ B1 | | | | | | | | | | | | | | | | | | □ B2 | | | | | | | | | | | □ C | | | | | | | | | | | | | | | | □ D | | | | | | | | | | | | | □ Ignored | |
| 50. AHA Classification | | | | | | | | | | | | | | | | | | □ A | | | | | | | | | □ B1 | | | | | | | | | | | | | | | | | | □ B2 | | | | | | | | | | | □ C | | | | | | | | | | | | | | | | □ D | | | | | | | | | | | | | □ Ignored | |
| 51. Los Andes Classification | | | | | | | | | | | | | | | | | |  | | | | | | | | | □ IA | | | | | | | | | | | | | | | | | | □ IB | | | | | | | | | | | □ II | | | | | | | | | | | | | | | | □ III | | | | | | | | | | | | | □ Ignored | |
| **Digestive findings** | | | | | | | | | | | | | | | | | | | | | | | | | | | | | | | | | | | | | | | | | | | | | | | | | | | | | | | | | | | | | | | | | | | | | | | | | | | | | | | | | | | | | | |
| 52. Digestive pathology detected? | | | | | | □ Yes | | | | | | | | | | | | | | | | | | | | | | | | | | | | | | | | | □ No | | | | | | | | | | | | | | | | | | | | | | | | | | | | | | | | | □ Not investigated | | | | | | | | | | | | | | |
| If detected, | | | | | |  | | | | | | | | | | | | | | | | | | | | | | | | | | | | | | | | |  | | | | | | | | | | | | | | | | | | | | | | | | | | | | | | | | |  | | | | | | | | | | | | | | |
| 52.1 Megacolon | | | | | | □ Yes | | | | | | | | | | | | | | | | | | | | | | | | | | | | | | | | | □ No | | | | | | | | | | | | | | | | | | | | | | | | | | | | | | | | | □ Not investigated | | | | | | | | | | | | | | |
| 52.2 Megaoesophagus | | | | | | □ Yes | | | | | | | | | | | | | | | | | | | | | | | | | | | | | | | | | □ No | | | | | | | | | | | | | | | | | | | | | | | | | | | | | | | | | □ Not investigated | | | | | | | | | | | | | | |
| 52.2.1 Rezende classification | | | | | | | | | | | | | | | | | | □ E0 | | | | | | | | | □ EI | | | | | | | | | | | | | | | | | | □ EII | | | | | | | | | | | □ EIII | | | | | | | | | | | | | | | | □ EIV | | | | | | | | | | | | | □ Not performed | |
| **Clinical status classification** | | | | | | | | | | | | | | | | | | | | | | | | | | | | | | | | | | | | | | | | | | | | | | | | | | | | | | | | | | | | | | | | | | | | | | | | | | | | | | | | | | | | | | |
| 53. Clinical form | | | | | | | | | | | | | | | | □ Control  □ Chronic  □ Acute | | | | | | | | | | | | | | | | | | | | | 53.1 If chronic, specify:  □ Chronic with non-demonstrable pathology (indeterminate form)  □ Chronic with cardiac pathology  □ Chronic with digestive pathology  □ Chronic with mixed pathology    53.2 If acute, specify:  □ First infection  □ Reactivation | | | | | | | | | | | | | | | | | | | | | | | | | | | | | | | | | | | | | | | | | | | | | | | | | |
| 1. **Treatment** | | | | | | | | | | | | | | | | | | | | | | | | | | | | | | | | | | | | | | | | | | | | | | | | | | | | | | | | | | | | | | | | | | | | | | | | | | | | | | | | | | | | | | |
| 54. Has the patient received etiologic treatment? | | | | | | | | | | | | | | | | □ Yes | | | | | | | | | | | | | | | | | | | | | | | | | | | | | | | | | | | | | | | | | | | | □ No | | | | | | | | | | | | | | | | | | | | | | | | | | |
| 54.1 If yes, what is the status of the treatment? | | | | | | | | | | | | | | | | | | | | | | | | | | | | | | | | | | | | | | | | | | | | | | | | | | | | □ Completed | | | | | | | | | | | | | | | | | | □ Ongoing | | | | | | | | | | | | | | | □ Interrupted | |
| Complete the following information just in case of completed or interrupted treatment: | | | | | | | | | | | | | | | | | | | | | | | | | | | | | | | | | | | | | | | | | | | | | | | | | | | | | | | | | | | | | | | | | | | | | | | | | | | | | | | | | | | | | | |
| 54.2 Drug | | | | | | | | | | | | | | | | | | | | | | □ BNZ | | | | | | | | | | | | | | | | | □ NFT | | | | | | | | | | | | | | □ Other, which one?__________________________ | | | | | | | | | | | | | | | | | | | | | | | | | | | | | | | | | |
| 54.3 Total administered dose | | | | | | | | | | | | | | | | | | | | | | | | | | | | | | | | | | | | | | | \|__\|__\|__\|__\|__\| (mg) | | | | | | | | | | | | | | | | | | | | | | | | | | | | | | | | | | | | | | | | | | | | | | | |
| 54.4 Total days of treatment | | | | | | | | | | | | | | | | | | | | | | | | | | | | | | | | | | | | | | | \|__\|__\|__\| | | | | | | | | | | | | | | | | | | | | | | | | | | | | | | | | | | | | | | | | | | | | | | | |
| 54.5 Start date | | | | | | | | | | | | | | | | | | | | | | | | | | | | | | | | | | | | | | | \|__\|__\| - \|__\|__\|__\| - \|__\|__\|__\|__\| | | | | | | | | | | | | | | | | | | | | | | | | | | | | | | | | | | | | | | | | | | | | | | | |
| 54.6 Completion date | | | | | | | | | | | | | | | | | | | | | | | | | | | | | | | | | | | | | | | \|__\|__\| - \|__\|__\|__\| - \|__\|__\|__\|__\| | | | | | | | | | | | | | | | | | | | | | | | | | | | | | | | | | | | | | | | | | | | | | | | |
| 54.7 If interrupted, specify the cause of interruption: _________________________________________  ___________________________________________________________________________________________________ | | | | | | | | | | | | | | | | | | | | | | | | | | | | | | | | | | | | | | | | | | | | | | | | | | | | | | | | | | | | | | | | | | | | | | | | | | | | | | | | | | | | | | |
| 54.8 Were adverse events associated with the treatment reported? | | | | | | | | | | | | | | | | | | | | | | | | | | | | | | | | | | | | | | | | | | | | □ Yes | | | | | | | | | | | | | | | | | | | | | | | | | | □ No | | | | | | | | | | | | | | | | |
| 54.8.1 If yes, specify _________________________________________________________________________________ | | | | | | | | | | | | | | | | | | | | | | | | | | | | | | | | | | | | | | | | | | | | | | | | | | | | | | | | | | | | | | | | | | | | | | | | | | | | | | | | | | | | | | |
| 55. Use of cardiovascular medication? | | | | | | | | | | | | | | | | | | | | | | | | | | | | | | | | | | | □ Yes | | | | | | | | | | | | | | | | | | | | | | | | | □ No | | | | | | | | | | | | | | | | | | | | | | | | | | |
| 55.1 If yes, please indicate which | | | | | | | | | | | | | | | | | | | | | | | | | | | | | | | | | | | | | | | | | | | | | | | | | | | | | | | | | | | | | | | | | | | | | | | | | | | | | | | | | | | | | | |
|  | □ Beta-blocker | | | | | | | | | | □ Angiotensin converting enzyme inhibitor | | | | | | | | | | | | | | | | | | | | | | | | | | | | | | | | | | | | | | | | | | | | | | □ Angiotensin receptor blocker | | | | | | | | | | | | | | | | | | | | | | | | | | □ Spironolactone | | | |
|  | □ Sacubitril/valsartan | | | | | | | | | | □ Sodium-glucose co-transporter 2 (SGLT2) inhibitors | | | | | | | | | | | | | | | | | | | | | | | | | | | | | | | | | | | | | | | | | | | | | | | | | | | □ Ivabradine | | | | | | | | | | | | | | | | | | | | | □ Furosemide | | | |
|  | □ Digoxin | | | | | | | | | | □ Oral anticoagulants | | | | | | | | | | | | | | | | | | | | | | | | | | | | | | | | | | | | | | | | | | | | | | □ Amiodarone | | | | | | | | | | | | | | | | | | | | | | | | | |  | | | |
| 1. **Biological samples** | | | | | | | | | | | | | | | | | | | | | | | | | | | | | | | | | | | | | | | | | | | | | | | | | | | | | | | | | | | | | | | | | | | | | | | | | | | | | | | | | | | | | | |
| 56. Have biological samples been collected? | | | | | | | | | | | | | | | | | | | | | | | | | | | | | | | | | | | □ Yes | | | | | | | | | | | | | | | | | | | | | | | | | □ No | | | | | | | | | | | | | | | | | | | | | | | | | | |
| **Type of sample**  Serum/plasma/whole blood/urine/saliva/tissue simples/PBMCs/ Other, specify | | | | **Number of aliquots** | | | | | | | | | | | | | | | **Volume of sample** | | | | | | | | | | | | | | | | | | | | | | | | **Sample ID** | | | | | | | | | | | | | | | | | **Date of collection** | | | | | | | | | | | | | | | | | | | | | | | | | | |
|  | | | |  | | | | | | | | | | | | | | |  | | | | | | | | | | | | | | | | | | | | | | | |  | | | | | | | | | | | | | | | | | \|__\|__\| - \|__\|__\|__\| - \|__\|__\|__\|__\| | | | | | | | | | | | | | | | | | | | | | | | | | | |
|  | | | |  | | | | | | | | | | | | | | |  | | | | | | | | | | | | | | | | | | | | | | | |  | | | | | | | | | | | | | | | | | \|__\|__\| - \|__\|__\|__\| - \|__\|__\|__\|__\| | | | | | | | | | | | | | | | | | | | | | | | | | | |
|  | | | |  | | | | | | | | | | | | | | |  | | | | | | | | | | | | | | | | | | | | | | | |  | | | | | | | | | | | | | | | | | \|__\|__\| - \|__\|__\|__\| - \|__\|__\|__\|__\| | | | | | | | | | | | | | | | | | | | | | | | | | | |
|  | | | |  | | | | | | | | | | | | | | |  | | | | | | | | | | | | | | | | | | | | | | | |  | | | | | | | | | | | | | | | | | \|__\|__\| - \|__\|__\|__\| - \|__\|__\|__\|__\| | | | | | | | | | | | | | | | | | | | | | | | | | | |
|  | | | |  | | | | | | | | | | | | | | |  | | | | | | | | | | | | | | | | | | | | | | | |  | | | | | | | | | | | | | | | | | \|__\|__\| - \|__\|__\|__\| - \|__\|__\|__\|__\| | | | | | | | | | | | | | | | | | | | | | | | | | | |

BNP, brain natriuretic peptide; BNZ, benznidazole; COPD, chronic obstructive pulmonary disease; CRT-D cardiac resynchronization therapy defibrillator ; CRT-P, cardiac resynchronization therapy pacemaker; E, peak early wave diastolic filling velocity; E’, peak early diastolic mitral annulus velocity; ICD, implantable cardioverter-defibrillator; LV, left ventricular; MRI, magnetic resonance imaging; NFT, nifurtimox; RV, right ventricular.
