# Supplementary material for: A standardized clinical database for research in Chagas disease: The NHEPACHA network
Source: PLoS Negl Trop Dis. 2024 Aug 15;18(8):e0012364. doi: 10.1371/journal.pntd.0012364 (PMC11326575; doi:10.1371/journal.pntd.0012364)
Supplement: S2 File — (DOCX) [file pntd.0012364.s002.docx]

**General Instructions**

In the clinical questionnaire for patients with Chagas disease, specific data are requested, summarizing detailed information from a complete medical history. This guide serves as a supplement to assist in capturing this data, both through the REDCap platform and in the physical questionnaire.

In REDCap, some questions allow for multiple selections, indicated by square checkboxes. Other questions only permit a single response, marked with a circular checkbox. If there is not enough information to answer a question (for instance, if a particular diagnostic test has not been conducted), the option 'Ignored' should be selected. In all cases, please refrain from using abbreviations when completing the questionnaire.

The questionnaire should be filled with the patient's current health status at the time of data and sample collection. Regarding laboratory information and imaging studies, ideally, results from the past 6 months should be reported.

Within the digital REDCap questionnaire, certain fields will need to be completed during each visit/review/follow-up scheduled for the patient, marked as 'Required Field'. Other fields will only be mandatory the first time the questionnaire is filled out, and indicated as '**Collects data only once**'. If any of these sections need to be edited, it can be done by selecting the 'Preview' option, followed by 'Current Case.' This allows viewing and editing of previously collected data.

Below are instructions for collecting specific questions of particular importance, organized by section number and question. If you encounter any issues or have doubts about any questions not listed here, you can ask by clicking on the symbol
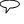
, located next to the question in the REDCap questionnaire. If using the physical questionnaire, please communicate directly with one of the authors.

**Section I: Visit Data**

The three pieces of information collected in this section must be gathered at each visit.

Question 1: The patient ID is generated by each institution and represents the number used to identify the patient, ensuring consistency across all their visits.

The NEPACHA prefix is the name identifying the institution within the network.

Question 2: The visit date refers to the appointment time for the patient's review. In the physical questionnaire, it should be noted as the day, the first three letters of the month, and the complete year. For example: 19-Sep-2022.

**Section III: Patient Information**

While not specified in the questionnaire or possible in the REDCap platform, it is recommended at this point for the interviewer to request the patient's phone number or that of a close contact, as well as an email address, to ensure communication, if necessary.

**Section IV: Epidemiological Information**

Question 11: When asking for the patient's country of origin, the aim is to determine if they come from an endemic area for Chagas disease.

Question 12: If the patient does not come from any endemic country, the country of origin of the mother is requested to assess the possibility of vertical transmission of the infection. In REDCap, this happens automatically. However, if using the physical format, the interviewer should note that Question 12 is only answered if the patient comes from a non-endemic area.

Question 13: Refers to the possible transmission mechanism. Establishing the transmission mechanism with certainty is challenging, but thorough inquiry is suggested. Below are a series of questions that may guide this process:

*To inquire about the vector-borne mechanism, it is suggested to ask the following questions:*

- Do you recognize the vector (bug/kissing bug/barber bug/cone-nose bug)? It is recommended to show an image with several insects, including species found in the residence area or country of origin.
- Do you recall the presence of the vector (using the participant's term for the vector) inside the house or dwelling?
- Do you recall seeing the vector at the workplace?
- Do you remember having a bump associated with the vector's bite?
- Have you seen the vector while camping or in rural areas?

*To inquire about the oral mechanism, the following questions are suggested:*

- Do you have a habit of consuming açaí pulp, sugarcane, or guava juice, or any other tropical fruit?

*To inquire about the vertical mechanism, the following questions are suggested:*

- Do you know if your maternal grandmother, mother, or siblings have been diagnosed with Chagas disease?

*To inquire about the occupational accident mechanism, the following question is suggested:*

- What is your occupation? Besides healthcare workers/researchers handling samples, consider at risk those who spend nights in the wilderness while hunting, as well as those handling meats and blood from animals for unregulated human consumption.

*For the transfusion and transplant mechanisms, the following questions are suggested:*

- Have you received a blood transfusion? For what reason? When was it administered?
- Have you received a transplant? For what reason? When was it performed?

Question 24: The cardiac devices acronyms are described at the end of the questionnaire. The person applying the questionnaire should check the participant’s cardiac device card and medical record for information regarding the specific cardiac device used by the participant.

**Section V: Etiological Diagnosis**

This section requires listing all tests conducted on the patient to establish the etiological diagnosis. This includes parasitological, serological, and molecular tests. For serological tests, at least two positive results are necessary to confirm the diagnosis. The questionnaire allows input for up to three serological tests and two molecular tests. In serological tests, the cutoff value refers to the threshold above which the test result is considered positive.

**Section VI: Clinical Picture**

Question 32: This question involves assessing the presence of clinical signs of heart failure according to the New York Heart Association (NYHA) scale, outlined below.

| Functional classification of the severity of dyspnea according to the New York Heart Association (NYHA) | |
| --- | --- |
| Grade I | The patient experiences dyspnea when making significant efforts such as running, climbing several flights of stairs, or engaging in intense sports, activities they could previously do without discomfort. |
| Grade II | The patient experiences dyspnea during moderate efforts such as walking, running a short distance, or climbing one flight of stairs. |
| Grade III | The patient experiences dyspnea during mild efforts such as combing hair, getting dressed, talking, or eating. |
| Grade IV | The patient experiences dyspnea at rest. |

Question 37: The REDCap platform questionnaire will only break down the capture of vital signs if the option 'Taken' has been marked for this question.

**Section VII: Diagnostic test results**

Questions 39 and 40: These questions refer to the presence of specific electrocardiographic signs of Chagasic cardiomyopathy. If the patient has any electrocardiographic alteration not listed in question 40, select the option 'Nonspecific alterations' in question 39. If the patient has any electrocardiographic alteration listed in question 40, please mark it in the corresponding checkbox.

Questions 41 to 46: These questions refer to echocardiogram, chest x-ray, Holter, cardiac MRI, and BNP/NT-proBNP serum levels. All data from all participants should be collected, whenever available.

Questions 41 and 42: If the patient presents any alteration not listed in question 42, indicate the option "presence of non-Chagas alterations" in question 41. If the patient has any echocardiographic alteration listed in question 42, please tick the corresponding box.

For question 46, below are the values of BNP and NT-proBNP:

| **Clinical scenario** | **BNP** | **NT-proBNP** |
| --- | --- | --- |
| Nonacute patients | < 35 pg/mL | < 125 pg/mL |
| Acute patients | < 100 pg/mL | < 300 pg/mL |
| Half life | 20 minutes | 120 minutes |

**Section VIII: Classifications**

Below is a table to aid in clinically classifying a patient according to different classifications included in Section VIII.

Questions 47-51:

| Classifications of Chagas disease based on abnormalities in imaging studies and symptoms. | | | | | |
| --- | --- | --- | --- | --- | --- |
| Abnormalities in imaging studies and symptoms. | Classifications | | | | |
|  | Kuschnir | Brazilian consensus | Los Andes | Latin-American | AHA |
| ECG normal and apparently without significant conduction abnormalities | 0 | NA | IA | A | A^a^ |
| ECG normal with contractile anomalies | NA | NA | IB | NA | B1 |
| Abnormal ECG, apparently without significant conduction abnormalities | I | A | NA | B1 | B1 |
| Abnormal ECG, contractile anomalies with normal left ventricular ejection fraction (LVEF) | NA | B1 | II | B1 | B1 |
| Abnormal ECG, likely conduction abnormalities or abnormal left ventricular ejection fraction (LVEF) | II | B1 (LVEF ≥ 45%), B2 (LVEF ≤ 45%) | II | B2 | B2 |
| Decompensated heart failure | III | C (compensated) | III | C (compensated) | C (compensated) |
| Refractory heart failure | NA | D (refractory) | NA | D (refractory) | D (refactory) |
| ECG: electrocardiogram; LVEF: Left ventricular ejection fraction; NA: Non-applicable; AHA: American Heart Association; a: No digestive alterations | | | | | |

Question 52.2.1:

Rezende Classification for Esophageal Achalasia:

| Rezende Classification | |
| --- | --- |
| Grade 1 | Initial form, oesophageal body with a diameter < 4 cm. |
| Grade 2 | Dilated oesophagus with a diameter > 4 cm but < 7 cm. |
| Grade 3 | Oesophageal diameter between 7 and 10 cm. |
| Grade 4 | Diameter > 10 cm, with a sinuous axis typical of a dolicho-aesophagus. |

Question 53: Patients diagnosed with *Trypanosoma cruzi* infection should be classified as acute or chronic based on their symptoms and time since the probable infection. If the patient is classified as chronic, the type of detected organic damage should be specified. In the REDCap platform, these options will only appear if the 'Chronic' checkbox is selected.

In case the participant is part of the control group in a clinical research study, the corresponding checkbox should be marked.

**Section IX: Treatment.**

Question 54: If the patient has received etiological treatment with benznidazole or nifurtimox, or any anti-parasitic medication in an experimental phase within the context of a clinical trial, the 'Yes' checkbox should be marked. Questions 54.1-54.8 should only be answered in this case.

Question 54.1 refers to the current status of treatment, whether the patient completed the full treatment regimen, interrupted it before completion, or is still receiving it (Ongoing). In the REDCap platform, questions 54.1-54.8 will only appear if 'Yes' is marked in question 54.

Question 55: Check all cardiovascular drugs the participant is using on the visit date.

**Section X: Biological Samples**

Question 56: This question requires indicating if biological samples have been obtained from the patient. If yes, the corresponding table must be completed. In the REDCap platform, this table will appear only if 'Yes' is marked in question 55. At this point, information about the type of sample, number of aliquots, volume, identifier, and sample collection date will be requested. For filling out this section, it's recommended to seek support from the individual responsible for sample collection.

**Glosary**

BNP, brain natriuretic peptide.

BNZ, benznidazole.

COPD, chronic obstructive pulmonary disease.

CRT-D cardiac resynchronization therapy defibrillator.

CRT-P, cardiac resynchronization therapy pacemaker.

E, peak early wave diastolic filling velocity.

E’, peak early diastolic mitral annulus velocity.

ICD, implantable cardioverter-defibrillator.

LV, left ventricular.

MRI, magnetic resonance imaging.

NFT, nifurtimox.

RV, right ventricular.
