# Supplementary material for: A standardized clinical database for research in Chagas disease: The NHEPACHA network
Source: PLoS Negl Trop Dis. 2024 Aug 15;18(8):e0012364. doi: 10.1371/journal.pntd.0012364 (PMC11326575; doi:10.1371/journal.pntd.0012364)
Supplement: S3 File — (DOCX) [file pntd.0012364.s003.docx]

**Título: Una base de datos clínica estandarizada para la investigación en la enfermedad de Chagas: Red NHEPACHA**

**Autores**: Adriana González Martínez^1,2^, Irene Losada Galván^3,4^, Juan Carlos Gabaldón-Figueira^3^, Nieves Martínez-Peinado^3,5^, Roberto Magalhães Saraiva^6^, Marisa Liliana Fernández^7^, Janine M Ramsey^8^, Oscar Noya-González^9,10, 11^, Belkisyole Alarcón de Noya^10^, Alejandro Gabriel Schijman^12^, Soledad Berón^13^, Marcelo Abril^13^, Joaquim Gascón^3,14^, Sergio Sosa-Estani^15,16^, María Jesús Pinazo^15^, Julio Alonso-Padilla^3,14^*, Alejandro Marcel Hasslocher-Moreno^6^* en representación de la red NHEPACHA**

Autor de correspondencia*:

Alejandro Marcel Hasslocher-Moreno

Email: [alejandro.hasslocher@gmail.com](mailto:alejandro.hasslocher@gmail.com)

Julio Alonso-Padilla

Email: [julio.a.padilla@isglobal.org](mailto:julio.a.padilla@isglobal.org)

**La lista de miembros completa de la red NHEPACHA se presenta en la sección de agradecimientos

**Afiliaciones:**

^1^Departamento de Investigación, Salvando Latidos A.C., Guadalajara, Mexico.

^2^Departamento de Investigación, Instituto Cardiovascular de Mínima Invasión (ICMI), Guadalajara, Mexico.

^3^Barcelona Institute for Global Health (ISGlobal), Hospital Clínic - Universitat de Barcelona, Barcelona, Spain.

^4^Hospital Universitario 12 de Octubre, Madrid, Spain.

^5^Secció de Parasitologia, Departament de Biologia, Sanitat i Medi Ambient, Facultat de Farmàcia
i Ciències de l'Alimentació, Universitat de Barcelona, 08007 Barcelona, Spain.

^6^Evandro Chagas National Institute of Infectious Diseases, Fundação Oswaldo Cruz, Rio de Janeiro, Brasil.

^7^Instituto Nacional de Parasitología Dr M. Fatala Chabén, Administración Nacional de Laboratorios e Institutos de Salud Dr C. Malbrán, Ministerio de Salud, Buenos Aires, Argentina.

^8^Centro Regional de Investigación en Salud Pública (CRISP), Instituto Nacional de Salud Pública (INSP), Tapachula, Chiapas, México.

^9^Cátedra de Parasitología, Escuela ¨Luís Razetti” Facultad de Medicina, Universidad Central de Venezuela, Caracas, Venezuela.

^10^Instituto de Medicina Tropical, Facultad de Medicina, Universidad Central de Venezuela, Caracas, Venezuela.

^11^Centro para Estudios Sobre Malaria, Instituto de Altos Estudios “Dr. Arnoldo Gabaldón”, Ministerio del Poder Popular para la Salud (MPPS), Caracas, Venezuela

^12^Laboratorio de Biología Molecular de la Enfermedad de Chagas, Instituto de Investigaciones en Ingeniería Genética y Biología Molecular “Dr. Héctor N. Torres” - INGEBI-CONICET, Buenos Aires, Argentina.

^13^Fundación Mundo Sano, Buenos Aires, Argentina.

^14^CIBER de Enfermedades Infecciosas, Instituto de Salud Carlos III (CIBERINFEC, ISCIII), Madrid, Spain.

^15^Drugs for Neglected Diseases Initiative (DNDi), Geneva, Switzerland.

^16^Centro de Investigaciones Epidemiológicas y Salud Pública, Consejo Nacional de Investigaciones Científicas y Técnicas, Buenos Aires, Argentina.

**Resumen**

La Red Iberoamericana NHEPACHA, creada a iniciativa de un grupo de investigadores de países latinoamericanos y de España, tiene como objetivo establecer un marco de investigación para la enfermedad de Chagas que abarque su diagnóstico y tratamiento. Con este propósito, la red ha elaborado un cuestionario para recopilar datos relevantes sobre aspectos epidemiológicos, clínicos, diagnósticos y terapéuticos de la enfermedad. Este cuestionario fue desarrollado a partir de un consenso entre los miembros expertos de la red, con la intención de recopilar datos estandarizados de alta calidad que puedan ser utilizados de manera intercambiable por los distintos centros de investigación que conforman la red NHEPACHA. Además, la red tiene la intención de ofrecer un protocolo clínico que pueda ser empleado por otros investigadores, facilitando la comparabilidad entre los estudios publicados.

**Palabras clave**: Enfermedad de Chagas; base de datos clínica; red

**Introducción**

La Organización Mundial de la Salud incluye la enfermedad de Chagas entre una de las veinte enfermedades tropicales desatendidas [1]. Surgida originalmente en regiones endémicas de América, principalmente América Latina, la incidencia de la enfermedad de Chagas se ha expandido al hemisferio norte y actualmente representa un problema de salud pública en los Estados Unidos de América, Europa y Japón [2]. La presencia de la enfermedad en países desarrollados no endémicos ha motivado la participación de diversas instituciones de salud gubernamentales y centros de investigación, en esfuerzos de control, diagnóstico y tratamiento [3,4].

La enfermedad de Chagas tiene dos fases clínicas bien definidas: la fase aguda, que ocurre poco después de la infección, y la fase crónica, que se extiende a lo largo de la vida de las personas infectadas. En la fase crónica, aproximadamente dos tercios de los individuos no desarrollan ninguna enfermedad clínica aparente, ni signos detectables de daño orgánico. El tercio restante puede presentar daño en tejidos cardiacos y/o digestivos que provocan su disfunción. Así, la fase crónica se clasifica en formas clínicas conocidas como indeterminada, cardíaca, digestiva y mixta [5]. La fase crónica no es estática, lo que significa que los pacientes pueden progresar de formas indeterminadas a formas sintomáticas, a una tasa anual promedio estimada en 1.9% [6]. [Del mismo modo, las personas también pueden transitar entre diferentes etapas clínicas de enfermedad cardíaca y digestiva [7,8]. Una de las mayores limitaciones en el manejo de pacientes en etapa indeterminada es la ausencia de biomarcadores para evaluar la progresión de la enfermedad. [9]

Respecto al tratamiento etiológico de la enfermedad de Chagas, las directrices recomiendan de manera inequívoca el tratamiento obligatorio durante la fase aguda, incluyendo la reactivación de infecciones crónicas, casos congénitos y casos en niños y adolescentes. De igual modo, recomiendan firmemente el tratamiento para mujeres en edad fértil y para adultos infectados con *T. cruzi* hasta los 50 años de edad con función miocárdica normal [10]. En la actualidad, varios estudios han demostrado que el uso de fármacos tripanocidas puede alterar la historia natural de la enfermedad de Chagas, reduciendo el riesgo de progresión de la enfermedad [11-13], y la carga parasitaria media, reduciendo también el riesgo de transmisión [14].

La evaluación médica es esencial para caracterizar el diagnóstico de la enfermedad de Chagas, determinar el grado de afectación cardíaca y/o digestiva, estratificar riesgos y guiar el tratamiento del paciente. La evidencia clínica es fundamental para orientar a los médicos en este proceso, ayudándoles a seleccionar los enfoques diagnósticos y terapéuticos más efectivos. Es importante destacar que la evidencia médica es dinámica y evoluciona continuamente a medida que se realizan nuevas investigaciones [15].

La investigación clínica en la enfermedad de Chagas comenzó en la década de 1920 con el trabajo pionero de Carlos Chagas y Eurico Vilela. En la década de 1930, Evandro Chagas sistematizó los aspectos clínicos y diagnósticos de la cardiopatía chagásica, que finalmente fue presentada como una característica definitoria de la enfermedad a la comunidad científica internacional por Laranja en la década de 1950 [16-18]. Desde entonces, se han publicado numerosos estudios sobre los aspectos clínicos, diagnóstico y tratamiento de la enfermedad de Chagas [19]. Tomaría más de 50 años que la mayoría de las directrices y manuales patrocinados por los gobiernos fueran producidos, ya en el siglo XXI, y respaldaran las políticas públicas para abordar y controlar la enfermedad [20]. Simultáneamente, durante este período, surgieron los primeros ensayos clínicos prospectivos aleatorizados con el objetivo de responder preguntas relacionadas con el pronóstico y tratamiento de la enfermedad de Chagas [21]. Estos ensayos resaltaron la urgente necesidad de identificar biomarcadores asociados con la eficacia terapéutica, basados en criterios parasitológicos, serológicos o clínicos [22]. Avances en este campo se han logrado recientemente [23].

La estandarización de datos epidemiológicos, diagnósticos, clínicos y terapéuticos para la enfermedad de Chagas es esencial para instituciones de referencia que tratan a pacientes con esta enfermedad y para grupos de investigación que siguen cohortes en estudios transversales y longitudinales. Las bases de datos multicéntricas son fundamentales para la investigación médica, ya que permiten a los investigadores analizar grandes volúmenes de información clínica para responder preguntas de investigación, evaluar la eficacia del tratamiento e identificar tendencias epidemiológicas. Una base de datos clínica para la investigación es una colección organizada y estructurada de información y datos relacionados con los pacientes y sus condiciones médicas, recopilados con el propósito de llevar a cabo investigaciones clínicas o estudios epidemiológicos. Existen diferentes tipos de bases de datos clínicas, incluyendo bases de datos de investigación clínica que recopilan datos específicamente con fines de investigación, bases de datos de registro de pacientes que registran información de pacientes en entornos clínicos de rutina, y bases de datos de vigilancia epidemiológica que monitorean la propagación de enfermedades [24].

La Red Iberoamericana NHEPACHA fue creada a iniciativa de un grupo de investigadores de países latinoamericanos y España en marzo de 2012. En la actualidad, comprende 12 grupos de investigación de 9 países. Sus objetivos son: (i) establecer un marco de investigación, desarrollo e innovación para identificar posibles nuevos fármacos y biomarcadores que ayuden en el diagnóstico y tratamiento de la enfermedad de Chagas, así como en la evaluación de nuevos fármacos en futuros ensayos clínicos; (ii) fomentar el intercambio de conocimientos; y (iii) planificar estudios operativos y ensayos clínicos multicéntricos para probar nuevas herramientas y mejorar las existentes [25].

En línea con este objetivo, NHEPACHA ahora ofrece un cuestionario clínico (Archivo suplementario 4) y un manual de cumplimentación del cuestionario (Archivo suplementario 5) a la comunidad de investigación clínica de la enfermedad de Chagas para facilitar la recopilación de datos estandarizados y comparables, con el objetivo de abordar preguntas específicas de investigación relacionadas con la evaluación de la eficacia del tratamiento, la identificación de factores de riesgo o la evaluación de resultados clínicos.

**Materiales y métodos**

**Aspectos éticos**

La elaboración de este cuestionario no requirió la obtención de aprobación ética, ya que no se utilizaron datos de pacientes en ningún paso del proceso. El uso de este cuestionario en contextos de investigación clínica debe llevarse a cabo de acuerdo con los principios de la Declaración de Helsinki, en conformidad con la normativa local y tras la aprobación por parte de un comité independiente de ética en la investigación.

**Metodología**

El contenido del cuestionario es resultado de un consenso de expertos que forman parte del grupo NHEPACHA. Se llevaron a cabo varias reuniones con el objetivo de crear un formulario de recopilación de datos clínico-epidemiológicos que abordara preguntas importantes de investigación en la enfermedad de Chagas. La información incluida en el formulario se organizó en las siguientes categorías: información de la visita; información institucional; información del paciente; información epidemiológica; diagnóstico etiológico; presentación clínica; resultado de pruebas diagnósticas; tratamiento; y muestras biológicas. Una vez definidos los elementos mínimos y esenciales del cuestionario, se hicieron esfuerzos para adherirse a a) lineamientos nacionales e internacionales que incluyan la información mínima clave a recolectar, y b) estándares establecidos que incluyan aspectos importantes como la privacidad y seguridad de los pacientes, bajo la evaluación ética de un comité institucional, así como la recopilación consistente de datos y la estandarización de datos para garantizar su calidad.

El cuestionario clínico acordado finalmente ha sido trasladado a formato electrónico, utilizando el software de captura de datos electrónicos REDCap [26,27]. REDCap (Research Electronic Data Capture) es una plataforma segura, basada en la web, y diseñada para apoyar la captura de datos en estudios de investigación, proporcionando: 1) una interfaz intuitiva para la captura validada de datos; 2) seguimiento de auditoría para rastrear la manipulación y exportación de datos; 3) procedimientos de exportación automatizados para descargas de datos sin interrupciones a paquetes estadísticos comunes; y 4) procedimientos para la integración de datos y la interoperabilidad con fuentes externas. REDCap es gratuito para organizaciones sin fines de lucro que se unen al Consorcio REDCap, y se utiliza ampliamente en la comunidad de investigación académica. Utilizar una base de datos centralizada basada en REDCap y alojada en ISGlobal permitirá a los centros de investigación de NHEPACHA utilizarla de forma independiente y colaborativa.

**Resultados**

*I. Información de la visita*: el paciente se identifica con un código, junto con la fecha de la visita.

*II. Información institucional:* incluye el nombre del médico entrevistador, nombre de la institución participante, su localización y la fecha y número del documento de aprobación del comité de ética del proyecto en cuestión, así como información sobre el consentimiento informado del participante.

*III. Información del paciente:* fecha de nacimiento y género del paciente.

*IV. Información epidemiológica:* se identifica el país de origen del paciente y el de su madre, si residen en un área rural o urbana, cuánto tiempo han estado alejados del área endémica original y si han vivido en otros países. En la sección de antecedentes familiares, se indica si hay familiares con enfermedad de Chagas y el grado de parentesco con el paciente. Para pacientes mujeres, se proporciona el historial de embarazos, el número de hijos e información sobre si los niños fueron evaluados para la enfermedad de Chagas en su primer año de vida. Se indica el mecanismo de transmisión más probable. Además, se señala cualquier comorbilidad, coinfección o si el paciente utiliza algún dispositivo intracardíaco (como marcapasos).

*V. Diagnóstico:* se incluyen los resultados de las pruebas serológicas realizadas (incluyendo nombre comercial, tipo y títulos de anticuerpos), así como de cualquier prueba parasitológica realizada. De igual manera, si se realizó una prueba molecular, se proporciona la técnica específica utilizada y los resultados cualitativos o cuantitativos obtenidos.

*VI. Cuadro clínico*: se presentan los signos y síntomas del paciente según la fase de la enfermedad. Para pacientes en la forma crónica, se obtiene información específica sobre la forma clínica observada. Se evalúan los signos de insuficiencia cardíaca y afectación digestiva, y se utiliza la clasificación de la New York Heart Association (NYHA) para evaluar el estado funcional del paciente. También se evalúan de forma independiente los signos asociados con la infección congénita. Se registran los datos del examen físico.

*VII. Resultados de pruebas diagnósticas:* se proporciona una lista de verificación para evaluar la presencia de hallazgos destacables en el electrocardiograma, ecocardiograma transtorácico, radiografía de tórax, Holter de 24 horas, imágenes de resonancia magnética nuclear (RMN) cardíaca y valores de BNP y pro-BNP; para una evaluación objetiva de la afectación cardíaca del paciente.

*VIII. Escalas clínicas:* la cardiopatía se estratifica utilizando clasificaciones clínicas preestablecidas de la enfermedad (Los Andes modificada, Kuschnir, Consenso Brasileño, Lineamientos Latinoamericanos y American Heart Association) [5,28-31]. Se caracteriza la forma digestiva, si está presente, y se utiliza la clasificación de Rezende para evaluar el grado de afectación esofágica [32]. Se identifica si la fase aguda es una infección primaria o la reactivación de una enfermedad crónica. Se caracteriza la forma crónica y se divide en indeterminada, cardíaca, digestiva o mixta.

*IX. Tratamiento:* se proporcionan datos sobre el tratamiento tripanocida recibido por el paciente. Se registran el fármaco, la fecha, la dosis y la duración del tratamiento, así como si el paciente experimentó algún efecto adverso y si se suspendió el tratamiento. Se incluye además información sobre el uso de medicamentos cardiovasculares, ya que su uso podría modificar el pronóstico de los pacientes con enfermedad cardíaca.

*X. Muestras biológicas*: en caso de que se hayan recogido muestras biológicas del paciente, se proporcionan datos sobre su tipo, número de alícuotas y volumen. Lineamientos a*d hoc* y protocolos operativos estándar (POE) para el manejo de estas muestras en el contexto de la red NHEPACHA también han sido preparados y se presentan en una publicación independiente.

**Discusión**

Un proceso estandarizado de recopilación de datos es fundamental para obtener datos de alta calidad y comparables en estudios clínicos de la enfermedad de Chagas. Una base de datos clínica uniforme es de vital importancia para acelerar avances en el diagnóstico y la evaluación del tratamiento de la enfermedad, ya que puede facilitar la colaboración entre diferentes equipos de investigación, agilizar el acceso a los datos y acelerar el ritmo general de la investigación, y proporcionar información sobre diferencias regionales derivadas de las características inmunológicas, genéticas, o proteómicas del parásito o su hospedero.

La herramienta presentada en este trabajo (en tres idiomas: inglés [archivos suplementarios 1 y 2], español [archivos suplementarios 3-5] y portugués [archivos suplementarios 6-8]) tiene el potencial de proporcionar un enfoque estandarizado para la recopilación de datos de pacientes, permitiendo a los investigadores evaluar diferentes resultados clínicos de manera diversa y completa, y acelerar la actualización de las pautas basadas en evidencia existentes, así como el desarrollo de nuevas.

Es importante destacar que los datos derivados del protocolo clínico de NHEPACHA también permitirán a los investigadores interesados, acceder a datos de pacientes anotados, ideales para llevar a cabo estudios retrospectivos, generar hipótesis o guiar el desarrollo de herramientas diagnósticas o terapéuticas innovadoras, al mismo tiempo que facilitan el seguimiento de la progresión clínica de un paciente a lo largo del tiempo. Esta perspectiva longitudinal es esencial, dada la naturaleza crónica de la enfermedad de Chagas y las brechas significativas en el conocimiento sobre la mejor manera de evaluar su progresión a largo plazo y la respuesta al tratamiento.

La naturaleza centralizada de esta base de datos elimina también las barreras para la adquisición y procesamiento de datos, permitiendo a investigadores de diversas instituciones acceder rápidamente a datos clínicos curados sin pasar por el proceso prolongado de recopilación y organización. Este enfoque colaborativo fomenta la investigación multidisciplinaria, el aprovechamiento de la experiencia y la capacidad para abordar preguntas complejas de investigación que requieren conjuntos de datos grandes y diversos.

Los investigadores pueden evitar duplicar esfuerzos en la recopilación de datos, ya que la información almacenada en la base de datos podría reutilizarse para múltiples proyectos de investigación. Esta eficiencia puede contribuir a ahorrar tiempo y recursos. Dada su naturaleza estandarizada y el hecho de que es fruto de discusiones de consenso dentro de la red de expertos de NHEPACHA, otros investigadores que también la utilicen podrán validar sus hallazgos al comparar sus resultados con los de sus pares dentro de la red.

En resumen, la homogeneización de bases de datos clínicas para la investigación es una herramienta esencial para la investigación médica moderna y desempeña un papel fundamental en el avance del desarrollo de nuevas herramientas diagnósticas y terapéuticas. En este contexto, el Formulario de Informe de Caso (CRF) de NHEPACHA, y su reflejo digitalizado alojado en REDCap (eCRF), representan un recurso notable para la recopilación y compartición de datos clínicos estandarizados de alta calidad. Esperamos que su implementación permita a los investigadores abordar una amplia gama de problemas, desde la validación de métodos diagnósticos y la evaluación de la eficacia de medicamentos, hasta comprender la dinámica compleja de la enfermedad a lo largo del tiempo.

**Agradecimientos**

Nos gustaría agradecer a DNDi y a Mundo Sano por su continuo apoyo a la red NHEPACHA.

**Grupo de estudio de la Red NHEPACHA:** Janine Ramsey W, Angelica Pech May, Alba Valdez Tah, Gilberto Sanchez Gonzalez, Adriana Gonzalez Martinez, Eduardo Ortiz Panozo, Mario J. Grijalva, Jaime A. Costales, Cesar A. Yumiseva, Carolina Herrera, Eileen Velez, Maria de Lourdes Torres, Maria Jesus Pinazo, Sergio Sosa Estani, Colin Forsyth, Eric Chatelain, Ivan Scandale, Fabiana Barreira, Tayná Marques, Marina Certo, Alejandro Hasslocher, Roberto Saraiva, Mauro Mediano, Andrea Silvestre, Sergio Xavier, Luiz Sangenis, Fernanda Mendes, Gilberto Sperandio da Silva, Andrea Costa, Henrique Veloso, Marcelo Holanda, Flavia Mazzoli, Paula Simplício da Silva, Tania Araujo, Mariana Wagabi, Luciana Garzoni, Constança Brito, Roberto Ferreira, Rita Machado, Raquel Aguiar, Marcelo Abril, Soledad Beron, Alejandro Schijman, Silvia Longhi, Arturo Muñoz-Calderón, Belkisyole Alarcon de Noya, Oscar Noya Gonzalez, Arturo Muñoz, Cecilia Colmenares, Ivan Mendoza, Zoraida Diaz, Raiza Ruiz, Ana Andreina Alviares, María Carmen Thomas, Manuel Carlos Lopez, Adriana Egui, Celia Benitez, Inmaculada Gómez, Francisco Macias Huete, Andres Mariano Ruiz, Rocio Rivero, Mónica Esteva, Margarita Bisio, Marisa Fernandez, Yolanda Hernandez, Julio Alonso Padilla, Joaquim Gascon, Irene Losada Galván, Nieves Martinez-Peinado, Juan Carlos Gabaldon-Figueira, María Gabriela Alvarez, Lococo Bruno, Laucella Susana, Flavio Andrés Tóman Conte, Dr. Enrique Morral, Maria Cecilia Albareda, Fernán Agüero, Emir Salas Sarduy, Alejandro Ricci, Leonel Bracco, Mercedes Didier Garnham, Alejandro Luquetti, Igor Almeida, Ester Sabino, Felipe Guhl, Faustino Torrico

**Referencias**

1. WHO. Chagas disease (American trypanosomiasis). 2020 [cited 12 Jul 2020]. Available: https://www.who.int/chagas/disease/en/

- 2. Gascon J, Bern C, Pinazo M-J. Chagas disease in Spain, the United States and other non-endemic countries. Acta Trop. 2010. Jul; 115 (1-2): 22–27. PMID: 19646412
- 3. Requena-Méndez A, Albajar-Viñas P, Angheben A, Chiodini P, Gascón J, Muñoz J, et al. Health Policies to Control Chagas Disease Transmission in European Countries.. PLoS Negl Trop Dis. 2014. Oct; 8 (10):e3245. PMID: 25357193
- 4. Gonzalez-Sanz M, Crespillo-Andújar C, Chamorro-Tojeiro S, Monge-Maillo B, Perez-Molina JA, Norman FF. Chagas Disease in Europe. Tropical Med. 2023. Dec; 8 (12): 513. PMID: 38133445

5. Dias JCP, Ramos Jr. AN, Gontijo ED, Luquetti A, Shikanai-Yasuda MA, Coura JR, et al. 2^nd^ Brazilian Consensus on Chagas Disease, 2015. Rev Soc Bras Med Trop. 2016. Dec; 49 (Suppl 1): 3–60.

- 6. Chadalawada S, Sillau S, Archuleta S, Mundo W, Bandali M, Parra-Henao G, et al. Risk of Chronic Cardiomyopathy Among Patients With the Acute Phase or Indeterminate Form of Chagas Disease: A Systematic Review and Meta-analysis. JAMA Network Open. 2020. Aug; 3 (8): e2015072. PMID: **32865573**
- 7. Saraiva RM, Mediano MFF, Quintana MSB, Sperandio da Silva GM, Costa AR, Sousa AS, et al. Two-dimensional strain derived parameters provide independent predictors of progression to Chagas cardiomyopathy and mortality in patients with Chagas disease. IJC Heart & Vasculature. 2022. Jan;38:100955. PMID: **35169612**

8. Castro C, Prata A, Macedo V. A folow-up period of 13 years prospective study in 190 chagasic patients of Mambaí, Goiás, State, Brazil. Rev Soc Bras Med Trop. 2001. Jul; 34 (4): 309–318.

- 9. Cortes-Serra N, Losada-Galvan I, Pinazo M-J, Fernandez-Becerra C, Gascon J, Alonso-Padilla J. State-of-the-art in host-derived biomarkers of Chagas disease prognosis and early evaluation of anti-*Trypanosoma cruzi* treatment response. Biochimica et Biophysica Acta (BBA) - Molecular Basis of Disease. 2020. Jul; 1866 (7): 165758. PMID: **32169507**
- 10. Organización Panamericana de la Salud. Síntesis de evidencia: Guía para el diagnóstico y el tratamiento de la enfermedad de Chagas. Revista Panamericana de Salud Publica. 2020. Jun;44: e28. PMID: 32523605
- 11. Viotti R, Vigliano C, Lococo B, Bertocchi G, Petti M, Alvarez MG, et al. Long-term cardiac outcomes of treating chronic Chagas disease with benznidazole versus no treatment: a nonrandomized trial. Ann Intern Med. 2006. May;144(10): 724–734. PMID: 16702588

12. Fabbro DL, Streiger ML, Arias ED, Bizai ML, del Barco ML, Amicone NA. Trypanocide treatment among adults with chronic Chagas disease living in Santa Fe City (Argentina), over a mean follow-up of 21 years: parasitological, serological and clinical evolution. Rev Soc Bras Med Trop. 2007. Feb; 40: 1–10.

- 13. Hasslocher-Moreno AM, Saraiva RM, Sangenis LHC, Xavier SS, de Sousa AS, Costa AR, et al. Benznidazole decreases the risk of chronic Chagas disease progression and cardiovascular events: A long-term follow up study. EClinicalMedicine. 2021. Dec;31: 100694. PMID: 33554085
- 14. Torrico F, Gascón J, Barreira F, Blum B, Almeida IC, Alonso-Vega C, et al. New regimens of benznidazole monotherapy and in combination with fosravuconazole for treatment of Chagas disease (BENDITA): a phase 2, double-blind, randomised trial. The Lancet Infectious Diseases. 2021. Aug;21(8): 1129–1140. PMID: 33836161

15. Alves S, Silva B, Barbosa E, Medeiros C, Barros M, Cavalcanti M, et al. Advances in Clinical Practice, Diagnosis and Treatment of Chronic Chagas’ Heart Disease. ABC Heart Fail Cardiomyop. 2023. Apr; 3(1): e20230029.

16. Chagas C, Villela E. Forma cardíaca da trypanosomiase americana. Mem Inst Oswaldo Cruz. 1922;14: 5–61.

17. Chagas E. Novos estudos sobre a forma cardiaca da Trypanosomiase americana. Mem Inst Oswaldo Cruz. 1932;26(3): 329–338.

- 18. Laranja FS, Dias E, Nobrega G, Miranda A. Chagas’ disease A clinical, epidemiologic, and pathologic study. Circulation. 1956. Dec;14(6): 1035–1060. PMID: 13383798
- 19. Ramos JM, González-Alcaide G, Gascón J, Gutierrez F. Mapping of Chagas disease research: analysis of publications in the period between 1940 and 2009. Rev Soc Bras Med Trop. 2011. Dec;44(6): 708–716. PMID: 22094704
- 20. Levin LG, Kreimer PR, Jensen P. Chagas Disease across Contexts: Scientific Knowledge in a Globalized World. Medical Anthropology. 2021. Sep;40(6): 572–589. PMID: 34237229
- 21. Tarleton RL. Avoiding Clinical Trial Failures in Neglected Tropical Diseases: The Example of Chagas Disease. Clinical Infectious Diseases. 2023. Apr; 17(76): 1516–1520. PMID: 36373213

22. Hasslocher-Moreno AM, Sperandio-da-Silva GM, Saraiva RM. Trypanocidal Treatment in Chronic Chagas Disease: Critical Evaluation of Cure Criteria. Exploratory Research and Hypothesis in Medicine. [2024. Jun;9(2):175-177](https://www.xiahepublishing.com/m/journal/erhm/current).

- 23. Morales‐Velásquez M, Barón‐Vera JP, Osorio‐Pulgarín MI, Sánchez‐Jiménez MM, Ospina‐Villa JD. Biomarkers for the diagnosis, treatment follow‐up, and prediction of cardiac complications in Chagas disease in chronic phase: Recent advances. Parasite Immunology. 2023. Dec;45(12): e13013. PMID: 37795913
- 24. Lee JY. Uses of Clinical Databases. The American Journal of the Medical Sciences. 1994. Jul;308(1): 58–62. PMID: 8010340

25. NHEPACHA: New Tools for the Diagnosis and Evaluation of Chagas Disease. 2023 [cited 7 Oct 2023]. Available: https://www.isglobal.org/en/-/nuevas-herramientas-para-el-diagnostico-y-la-evaluacion-del-paciente-con-enfermedad-de-chagas-nhepacha

- 26. Harris PA, Taylor R, Thielke R, Payne J, Gonzalez N, Conde JG. Research electronic data capture (REDCap)—A metadata-driven methodology and workflow process for providing translational research informatics support. Journal of Biomedical Informatics. 2009. Apr;42(2): 377–381. PMID: 18929686
- 27. Harris PA, Taylor R, Minor BL, Elliott V, Fernandez M, O’Neal L, et al. The REDCap consortium: Building an international community of software platform partners. Journal of Biomedical Informatics. 2019. Jul;95: 103208. PMID: **31078660**
- 28. Carrasco HAG, Barboza JS, Inglessis G, Fuenmayor A, Molina C. Left ventricular cineangiography in Chagas’ disease: Detection of early myocardial damage. Am Heart J. 1982. Sep;104(3): 595–602. PMID: 7113900
- 29. Kuschnir E, Sgammini H, Castro R, Evequoz C, Ledesma R, Brunetto J. Evaluation of cardiac function by radioisotopic angiography, in patients with chronic Chagas cardiopathy. Arq Bras Cardiol. 1985. Oct;45(4): 249–256. PMID: 3835868
- 30. Andrade JP de, Marin Neto JA, Paola AAV de, Vilas-Boas F, Oliveira GMM, Bacal F, et al. I Latin American Guidelines for the Diagnosis and Treatment of Chagas’ Heart Disease. Executive Summary. Arq Bras Cardiol. 2011. Jun;96(6): 434–442. PMID: 21789345
- 31. Nunes MCP, Beaton A, Acquatella H, Bern C, Bolger AF, Echeverría LE, et al. Chagas Cardiomyopathy: An Update of Current Clinical Knowledge and Management: A Scientific Statement From the American Heart Association. Circulation. 2018. Sep;138(12).169-209. PMID: 30354432

32. Rezende JM. Classificação Radiológica do Megaesôfago. Rev Goiana Med. 1982. Jul;28(3-4): 187–191.

**Información suplementaria**

**Archivo suplementario 1**: Cuestionario clínico de pacientes con Chagas (inglés).

**Archivo suplementario 2**: Manual de cumplimentación del cuestionario (inglés).

**Archivo suplementario 3**: A standardized clinical database for research in Chagas disease: NHEPACHA Network (español).

**Archivo suplementario 4**: Cuestionario clínico de pacientes con Chagas (español).

**Archivo suplementario 5**: Manual de cumplimentación del cuestionario (español).

**Archivo suplementario 6**: A standardized clinical database for research in Chagas disease: NHEPACHA Network (portugués).

**Archivo suplementario 7**: Cuestionario clínico de paciente con Chagas (portugués).

**Archivo suplementario 8**: Manual de cumplimentación del cuestionario (portugués).
