# Supplementary material for: A standardized clinical database for research in Chagas disease: The NHEPACHA network
Source: PLoS Negl Trop Dis. 2024 Aug 15;18(8):e0012364. doi: 10.1371/journal.pntd.0012364 (PMC11326575; doi:10.1371/journal.pntd.0012364)
Supplement: S4 File — (DOCX) [file pntd.0012364.s004.docx]

| 1. **Datos de la visita** | | | | | | | | | | | | | | | | | | | | | | | | | | | | | | | | | | | | | | | | | | | | | | | | | | | | |
| --- | --- | --- | --- | --- | --- | --- | --- | --- | --- | --- | --- | --- | --- | --- | --- | --- | --- | --- | --- | --- | --- | --- | --- | --- | --- | --- | --- | --- | --- | --- | --- | --- | --- | --- | --- | --- | --- | --- | --- | --- | --- | --- | --- | --- | --- | --- | --- | --- | --- | --- | --- | --- |
| 1. Identificacion del paciente | | | | | | | | | | \|__\|__\|__\| -  Prefijo centro NHEPACHA - | | | | | | | | | | | | | | | | | | | | | | | | | | | | ___________________________  Identificador del paciente en el centro | | | | | | | | | | | | | | |
| 2. Fecha de la visita | | | | | | | | | | \|__\|__\| - \|__\|__\|__\| - \|__\|__\|__\|__\| | | | | | | | | | | | | | | | | | | | | | | | | | | | | | | | | | | | | | | | | | | |
| 1. **Datos generales de la institución** | | | | | | | | | | | | | | | | | | | | | | | | | | | | | | | | | | | | | | | | | | | | | | | | | | | | |
| 3. Médico entrevistador: | | | | | | | | __________________________________________________________________________ | | | | | | | | | | | | | | | | | | | | | | | | | | | | | | | | | | | | | | | | | | | | |
| 4. Nombre de la Institución: | | | | | | | | | _________________________________________________________________________ | | | | | | | | | | | | | | | | | | | | | | | | | | | | | | | | | | | | | | | | | | | |
| 5. Localidad/Ciudad: | | | | | | | | _____________________ _____________________________________________________ | | | | | | | | | | | | | | | | | | | | | | | | | | | | | | | | | | | | | | | | | | | | |
| 6. Existe consentimiento informado: | | | | | | | | | | | | | | | | | | | | | | | | | | | □ Sí | | | | | | | | | | | | | □ No | | | | | | | | | | | | |
| 7. Fecha de aprobación del comité de ética | | | | | | | | | | | | | | | | | | | | | | | | | | | \|__\|__\| - \|__\|__\|__\| - \|__\|__\|__\|__\| | | | | | | | | | | | | | | | | | | | | | | | | | |
| 8. Número de folio de aprobación del comité de ética: | | | | | | | | | | | | | | | | | | | | | | | | | | | __________________________________________ | | | | | | | | | | | | | | | | | | | | | | | | | |
| 1. **Datos del paciente** | | | | | | | | | | | | | | | | | | | | | | | | | | | | | | | | | | | | | | | | | | | | | | | | | | | | |
| 9. Fecha de nacimiento | | | | | | | | | | | | | | | | | | | | | | | | | | | \|__\|__\| - \|__\|__\|__\| - \|__\|__\|__\|__\| | | | | | | | | | | | | | | | | | | | | | | | | | |
| 10. Sexo biológico | | | | | | | | | | | | | | | | | | | | | | | | | | | □ Masculino | | | | | | | | | | | | | □ Femenino | | | | | | | | | | | | |
| 1. **Información epidemiológica** | | | | | | | | | | | | | | | | | | | | | | | | | | | | | | | | | | | | | | | | | | | | | | | | | | | | |
| 11. País de origen | | | | | | □ Argentina  □ Belice  □ Bolivia  □ Brasil | | | | | | | | | | □ Chile  □ Colombia  □ Costa Rica  □ Ecuador | | | | | | | | | | | | | | | | □ El Salvador  □ Guatemala  □ Guyana  □ Honduras | | | | | | | | | | □ México  □ Nicaragua  □ Panamá  □ Paraguay | | | | | | | | □ Perú  □ Surinam  □ Uruguay  □ Venezuela | | |
|  |  |  |  |  |  | □ Otro, ¿Cuál? | | | | | | | | | | ___________________________________________________________ | | | | | | | | | | | | | | | | | | | | | | | | | | | | | | | | | | | | |
| 12. Si otro país de origen, país de origen de la madre: | | | | | | | | | | | | | | | | | | | | | | | | | | | | | | | | ___________________________________________ | | | | | | | | | | | | | | | | | | | | |
| 13. Posible mecanismo de transmisión | | | | | | | | | | | □ Desconocido  □ Transfusional | | | | | | | | | | | | | | | | | | | | | □ Oral  □ Trasplante | | | | | | | | | | | | | | □ Vectorial  □ Vertical (Congénito) | | | | | | |
|  |  |  |  |  |  |  |  |  |  |  | □ Accidente laboratorial | | | | | | | | | | | | | | | | | | | | | | | | | | | | | | | | | | | | | | | | | |
| 14. En caso de transfusión/trasplante/accidente laboratorial: | | | | Dónde:  _____________________ | | | | | | | | | | | | | | | | Motivo:  _____________________ | | | | | | | | | | | | | | | | | | | | | Fecha:  \|__\|__\| - \|__\|__\|__\| - \|__\|__\|__\|__\| | | | | | | | | | | | |
| 15. Reside en zona: | | | | | | | | | | | | | | | | | | | | | | | | | | | □ Rural | | | | | | | | | | | | | | □ Urbana | | | | | | | | | | | |
| 16. ¿Hace cuánto tiempo salió de área endémica? | | | | | | | | | | | | | | | □ No ha salido | | | | | | | | | | | | | | | | | | | | | | □ < 10 años | | | | | | | | | | | | □ > 10 años | | | |
| 17. Otros lugares donde ha vivido: | | | | | | | _____________________________________________________________________________ | | | | | | | | | | | | | | | | | | | | | | | | | | | | | | | | | | | | | | | | | | | | | |
| 18. ¿Tiene familiar con EChagas? | | | | | | | | | | □ Madre  □ Padre  □ Otros: ____________________________ | | | | | | | | | | | | | | | | | | | | | | | | | | | | □ Hermanos  □ Hijos | | | | | | | | | | | | | | |
| Si es mujer contestar de 19 a 21, si es hombre saltar a la 22: | | | | | | | | | | | | | | | | | | | | | | | | | | | | | | | | | | | | | | | | | | | | | | | | | | | | |
| 19. Está embarazada  20. Tiene hijos  21. ¿Sus hijos fueron estudiados para Chagas hasta los 8, 10 o 12 meses de vida? | | | | | | | | | | □ Sí  □ Sí    □ Sí | | | | | | | | | | | | | | | | | | | | | □ No  □ No    □ No | | | | | | | | □ No sabe  □ No sabe | | | | | | | | □ Ignorado  □ Ignorado | | | | | |
| 22. ¿Tiene alguna de estas comorbilidades? | | | | | | | | | | □ Sin dato  □ No tiene  □ Diabetes Mellitus  □ Hipotiroidismo  □ Hipertensión Arterial Sistémica  □ Otros, ¿Cuál? | | | | | | | | | | | | | | | | | | | | | □ Cardiopatía no-chagásica  □ Enfermedad hepática  □ Dislipidemia  □ Neoplasia  □ Alteración NeuroPsiquiatrica  _______________________ | | | | | | | | | | | | | | | | □ Enfermedad autoinmune  □ EPOC  □ Asma Bronquial  □ Evento tromboembólico | | | | | |
| 23. ¿Tiene alguna coinfección? | | | | | | | | | | □ Sin dato  □ Otros, ¿Cuál? | | | | | | | | | | | | | | | | | | | | | □ VIH  _______________________ | | | | | | | | | | | | | | | | □ Covid19 | | | | | |
| 24. Tiene dispositivo: | | | | | | | | | | □ No  □ Desfibrilador cardioversor implantable (ICD) | | | | | | | | | | | | | | | | | | | | | □ Marcapasos unicameral  □ Terapia de resincronización cardíaca con marcapasos (CRT-P) | | | | | | | | | | | | | | | | □ Marcapasos bicameral  □ Terapia de resincronización cardíaca con desfibrilador (CRT-D) | | | | | |
| 1. **Diagnóstico etiológico** | | | | | | | | | | | | | | | | | | | | | | | | | | | | | | | | | | | | | | | | | | | | | | | | | | | | |
| 25. ¿Prueba parasitológica realizada? | | | | | | | | | | □ Sí  □ No | | | | | | | | | | | | | 25.1 En caso afirmativo, ¿cuál?   \| □ Strout  □ Micrométodo \| \| --- \| \| □ Xenodiagnóstico  □ Otro, ¿cuál? _____________________ \| | | | | | | | | | | | | | | | | | | | | | | | | | | | | | |
| 25.2 Fecha de realización de la prueba parasitológica | | | | | | | | | | | | | | | | | | | | | | | \|__\|__\| - \|__\|__\|__\| - \|__\|__\|__\|__\| | | | | | | | | | | | | | | | | | | | | | | | | | | | | | |
| 25.3 Resultado | | | | | | | | | | □ No detectable | | | | | | | | | | | | | | | | | | | | | | | | | | | |  | | | | | | | | | | | | | | |
|  | | | | | | | | | | □ Detectable | | | | | | | | | | | | | | Valor cuantitativo_____________________ | | | | | | | | | | | | | | | | | | | | | | | | | | | | |
| 26. ¿Serología 1 realizada? | | | | | | | | | | □ Sí  □ No | | | | | | | | | | | | | 26.1En caso afirmativo, ¿cuál?  □ ELISA Ig M  □ ELISA Ig G  □ Test rápido  □ HAI  □ IFI  □ Quimioluminescencia (CMIA)  □ Otro, ¿cuál?_______________________ | | | | | | | | | | | | | | | | | | | | | | | | | | | | | |
| 26.2 Fecha de realización de serología 1 | | | | | | | | | | | | | | | | | | | | | | | | | | \|__\|__\| - \|__\|__\|__\| - \|__\|__\|__\|__\| | | | | | | | | | | | | | | | | | | | | | | | | | | |
| 26.3 Resultado | | | | | | | □ No detectable | | | | | | | | | | | □ Detectable | | | | | | | | | | | | | | | | | | | | Marca comercial______________________  Valor cuantitativo_____________________  Valor de cut off_______________________ | | | | | | | | | | | | | | |
|  | | | | | | | | | |  | | | | | | | | | | | | | | | | | | | | | | | | | | | |  | | | | | | | | | | | | | | |
|  | | | | | | | | | |  | | | | | | | | | | | | | | | | | | | | | | | | | | | |  | | | | | | | | | | | | | | |
| 27. ¿Serología 2 realizada? | | | | | | | | | | □ Sí  □ No | | | | | | | | | | | | | | | | | | | | | | | | | | | | 27.1En caso afirmativo, ¿cuál?  □ ELISA Ig M  □ ELISA Ig G  □ Test rápido  □ HAI  □ IFI  □ Quimioluminescencia (CMIA)  □ Otro, ¿cuál?_______________________ | | | | | | | | | | | | | | |
| 27.2 Fecha de realización de serología 2 | | | | | | | | | | \|__\|__\| - \|__\|__\|__\| - \|__\|__\|__\|__\| | | | | | | | | | | | | | | | | | | | | | | | | | | | |  | | | | | | | | | | | | | | |
| 27.3 Resultado | | | | | □ No detectable | | | | | | | | | | | | | | | | □ Detectable | | | | | | | | | | | | | | | | | Marca comercial______________________  Valor cuantitativo_____________________  Valor de cut off_______________________ | | | | | | | | | | | | | | |
| 28. ¿Serología 3 realizada? | | | | | | | | | | □ Sí  □ No | | | | | | | | | | | | | | | | | | | | | | | | | | | | 28.1En caso afirmativo, ¿cuál?  □ ELISA Ig M  □ ELISA Ig G  □ Test rápido  □ HAI  □ IFI  □ Quimioluminescencia (CMIA)  □ Otro, ¿cuál?_______________________ | | | | | | | | | | | | | | |
| 28.2 Fecha de realización de serología 3 | | | | | | | | | | \|__\|__\| - \|__\|__\|__\| - \|__\|__\|__\|__\| | | | | | | | | | | | | | | | | | | | | | | | | | | | |  | | | | | | | | | | | | | | |
| 28.3 Resultado | | | | | □ No detectable | | | | | | | | | | | | | □ Detectable | | | | | | | | | | | | | | | | | | | | Marca comercial______________________  Valor cuantitativo_____________________  Valor de cut off_______________________ | | | | | | | | | | | | | | |
| 29. ¿Prueba molecular 1 realizada? | | | | | | | | | | □ Sí  □ No | | | | | | | | | | | | | | | | | | 29.1 En caso afirmativo, prueba molecular 1:  □ PCR convencional  □ qPCR  □ LAMP  □ Otro, ¿cuál? _____________________________________ | | | | | | | | | | | | | | | | | | | | | | | | |
| 29.2 Fecha de realización de la prueba molecular 1 | | | | | | | | | | | | | | | | | | | | | | | | | | \|__\|__\| - \|__\|__\|__\| - \|__\|__\|__\|__\| | | | | | | | | | | | | | | | | | | | | | | | | | | |
| 29.3 Resultado | | | | | | | | | | □ No detectable | | | | | | | | | | | | | | | | | | | | | | | | | | | | □ Detectable  Valor cuantitativo_____________________ | | | | | | | | | | | | | | |
| 30. ¿Prueba molecular 2 realizada? | | | | | | | | | | □ Sí  □ No | | | | | | | | | | | | | | | | | | | | | | | | | | | | 30.1 En caso afirmativo, prueba molecular 1:  □ PCR convencional  □ qPCR  □ LAMP  □ Otro, ¿cuál? _____________________________________ | | | | | | | | | | | | | | |
| 30.2 Fecha de realización de la prueba molecular 2 | | | | | | | | | | | | | | \|__\|__\| - \|__\|__\|__\| - \|__\|__\|__\|__\| | | | | | | | | | | | | | | | | | | | | | | | | | | | | | | |  | | | | | | | |
| 30.3 Resultado | | | | | | | | | | □ No detectable | | | | | | | | | | | | | | | | | | | | | | | | | | | | □ Detectable  Valor cuantitativo____________________ | | | | | | | | | | | | | | |
| 1. **Cuadro clínico**   **Síntomas** | | | | | | | | | | | | | | | | | | | | | | | | | | | | | | | | | | | | | | | | | | | | | | | | | | | | |
| 31. Agudo | □ Ignorado  □ Ninguno   \| □ Chagoma \| \| --- \| \| □ Mialgia \| \| □ Artralgías \| \| □ Esplenomegalia \| \| □ Astenia \| \| □ Disnea  □ Cefalea  □ Otro: \| | | | | | | | | | | | | | | | | | | | | | \| □ Signo de Romaña \| \| --- \| \| □ Dolor abdominal \| \| □ Escalofrío \| \| □ Hepatomegalia \| \| □ Adinamia \| \| \| □ Dolor torácico  □ Linfadenopatía  □ Taquicardia \| \| | | | | | | | | | | | | | | | | | | | | | \| □ Fiebre \| \| \| --- \| --- \| \| □ Edema facial \| \| \| □ Edema en miembros inferiores \| \| \| \| □ Ictericia \| \| \| □ Postración \| \| □ Nódulos en miembros inferiores \| \| \| | | | | | | | | | |
| 32. Crónico cardiovascular | □ Ignorado  □ Ninguno   \| □ Mareos  □ Síncope \| \| --- \|   □ Palpitaciones  □ Edema periférico  □ Sibilancias  □ Otro, ¿cuál? | | | | | | | | | | | | | | | | | | | | | Clasificación NYHA:   \| □ Clase I \| \| --- \| \| □ Clase II  □ Clase III  □ Clase IV  □ Fatiga  □ Distensión abdominal \| | | | | | | | | | | | | | | | | | | | | | □ Aumento súbito de peso  □ Falta de aliento  □ Dolor torácico  Evento tromboembólico:   \| □ SI \| \| --- \| \| □ No  □ No sabe \| | | | | | | | | | |
| 33. Crónico digestivo | □ Ignorado  □ Ninguno  □ Disfagia  □ Diarrea  □ Otro, ¿cuál? | | | | | | | | | | | | | | | | | | | | | □ Odinofagia  □ Regurgitación | | | | | | | | | | | | | | | | | | | | | Estreñimiento   \| □ SI  □ <7días □>7días \| \| --- \| \| □ No \| | | | | | | | | | |
| 34. Vertical | □ Ignorado  □ Ninguno   \| □ Esplenomegalia  □ Linfadenopatía  □ Taquicardia  □ Cianosis  □ Otro, ¿cuál? \| \| --- \| | | | | | | | | | | | | | | | | | | | | | \| □ Bajo peso al nacer \| \| --- \| \| □ Prematuro \| \| □ Polipnea \| \| □ Irritabilidad/Apatía  □ Ictericia  □ Convulsiones \| | | | | | | | | | | | | | | | | | | | | | □ Hepatomegalia   \| □ Fiebre \| \| --- \| \| □ Edema generalizado  □ Bradicardia  □ Microcefalia \| | | | | | | | | | |
|  | | | | | | | | | | | | | | | | | | | | | | | | | | | | | | | | | | | | | | | | | | | | | | | | | | | | |
| **Signos/Examen físico** | | | | | | | | | | | | | | | | | | | | | | | | | | | | | | | | | | | | | | | | | | | | | | | | | | | | |
| 35. Peso ( kilogramos) | | | \|__\|__\|__\|.\|__\|__\| (Kg) | | | | | | | | | | | | | | | | | | | | | | | 36. Altura (en metros) | | | | | | | | | | | | | | | | | | | \|__\|.\|__\|__\| (m) | | | | | | | |
| 37. Signos vitales | | | □ Ignorado  37.1 Presión arterial | | | | | | | | | | | | | | □ Tomados  \|__\|__\|__/__\|__\|__\| | | | | | | | | | | | | | | | | | 37.2 Temperatura (´C) | | | | | | | | | | | | | | \|__\|__\|__\| | | | | |
|  |  |  | 37.3 Frecuencia respiratoria | | | | | | | | | | | | | | | | | | | | \|__\|__\|__\| | | | | | | | | | | | 37.4 Frecuencia cardiaca | | | | | | | | | | | | | | | | \|__\|__\|__\| | | |
|  |  |  | 37.5 Saturación de oxígeno | | | | | | | | | | | | | | | | | | | | | | | \|__\|__\|_%_\| | | | | | | | | | | | | | | | | | | |  | | | | | | | |
| 38. Signos de insuficiencia cardiaca | | | □ Ignorado  □ Ninguno   \| □ Edema de miembros inferiores \| \| --- \| \| □ Taquicardia  □ Estertores crepitantes  □ Otro, ¿cuál? \| | | | | | | | | | | | | | | | | | | | | | | | □ Ingurgitación yugular  □ Hepatomegalia  □ Galope a la auscultación (tercer ruido cardíaco) | | | | | | | | | | | | | | | | | | | □ Choque de punta  □ Pulso irregular | | | | | | | |
| 1. **Resultados de pruebas diagnósticas** | | | | | | | | | | | | | | | | | | | | | | | | | | | | | | | | | | | | | | | | | | | | | | | | | | | | |
| 39. Electrocardiograma | | | | | | | | | | □ No realizado  □ Alteraciones sugestivas de cardiopatía chagásica | | | | | | | | | | | | | | | | | | | | | | | | | | | | □ Sin alteraciones  □ Alteraciones inespecíficas | | | | | | | | | | | | | | |
|  | | | | | | | | | |  | | | | | | | | | | | | | | | | | | | | | | | | | | | | | | | | | | | | | | | | | | |
| 39.1 Fecha de realización de electrocardiograma  39.2: ¿El electrocardiograma presenta ritmo de marcapasos?    39.2.1 En caso afirmativo, por favor especificar:  □ ICD | | | | | | | | | | | | | | | | \|__\|__\| - \|__\|__\|__\| - \|__\|__\|__\|__\|  □ Sí □ No  □ICD □CRT-P □CRT-D | | | | | | | | | | | | | | | | | | | | | | | | | | | | | | | | | | | | |
| 40. Si hay alteraciones sugestivas de cardiopatía chagásica: | | | 40.1 Bloqueo completo de rama derecha | | | | | | | | | | | | | | | | | | | | | | | | | | | | | | | | | | | | | | | | | | | | | | | | □ Sí □ No | |
|  |  |  | 40.2 Bloqueo completo de rama izquierda | | | | | | | | | | | | | | | | | | | | | | | | | | | | | | | | | | | | | | | | | | | | | | | | □ Sí □ No | |
|  |  |  | 40.3 Hemibloqueo anterior de rama izquierda | | | | | | | | | | | | | | | | | | | | | | | | | | | | | | | | | | | | | | | | | | | | | | | | □ Sí □ No | |
|  |  |  | 40.4 Extrasístoles ventriculares frecuentes o repetitivas (más de 1) | | | | | | | | | | | | | | | | | | | | | | | | | | | | | | | | | | | | | | | | | | | | | | | | □ Sí □ No | |
|  |  |  | 40.5 Zonas eléctricamente inactivas (ondas Q ≥4 ms y/o profundidad de la onda Q ≥ al 25% de la onda Ren al menos 2 derivaciones) | | | | | | | | | | | | | | | | | | | | | | | | | | | | | | | | | | | | | | | | | | | | | | | | □ Sí □ No | |
|  |  |  | 40.6 Bloqueo A-V de segundo grado. | | | | | | | | | | | | | | | | | | | | | | | | | | | | | | | | | | | | | | | | | | | | | | | | □ Sí □ No | |
|  |  |  | 40.7 Bloqueo A-V completo. | | | | | | | | | | | | | | | | | | | | | | | | | | | | | | | | | | | | | | | | | | | | | | | | □ Sí □ No | |
|  |  |  | 40.8 Taquicardia ventricular sostenida y/o no sostenida | | | | | | | | | | | | | | | | | | | | | | | | | | | | | | | | | | | | | | | | | | | | | | | | □ Sí □ No | |
|  |  |  | 40.9 Taquicardia auricular y/o fibrilación auricular y/o aleteo auricular (flutter) | | | | | | | | | | | | | | | | | | | | | | | | | | | | | | | | | | | | | | | | | | | | | | | | □ Sí □ No | |
|  |  |  | 40.10 Bradicardia sinusal (<50 lpm)  40.11 Cambios primarios en la onda T | | | | | | | | | | | | | | | | | | | | | | | | | | | | | | | | | | | | | | | | | | | | | | | | □ Sí □ No  □ Sí □ No | |
|  |  |  | 40.12 Ritmo de marcapaso (alteración primaria de repolarización ventricular) | | | | | | | | | | | | | | | | | | | | | | | | | | | | | | | | | | | | | | | | | | | | | | | | □ Sí □ No | |
|  |  |  | 40.12 Otro, describir________________________________________________ | | | | | | | | | | | | | | | | | | | | | | | | | | | | | | | | | | | | | | | | | | | | | | | | □ Sí □ No | |
| 41. Ecocardiograma | | | □ No realizado | | | | | | | | | | | | | | | | | | | | | | | □ Sin alteraciones | | | | | | | | | | | | | | | | | | | □ Con alteraciones | | | | | | | |
|  | | | □ Presencia de alteraciones no chagásicas | | | | | | | | | | | | | | | | | | | | | | | | | | | | | | | | | | | | | | | | | | | | | | | | | |
| 41.1 Fecha de realización del ecocardiograma | | | | | | | | | | | | | | | | \|__\|__\| - \|__\|__\|__\| - \|__\|__\|__\|__\| | | | | | | | | | | | | | | | | | | | | | | | | | | | | | | | | | | | | |
|  | | | | | | | | | | 42.1 FEVI: \|__\|__\|% | | | | | | | | | | | | | | | | | | | | | | | Método: | | | | | | | | | □ Simpson | | | | | | | | □ Teicholtz | | |
| 42. Si hay alteraciones en ecocardiograma | | | | | | | | | | 42.2 Alteraciones segmentarias de la motilidad | | | | | | | | | | | | | | | | | | | | | | | □ Sí | | | | | | | | | □ No | | | | | | | | □ Ignorado | | |
|  |  |  |  |  |  |  |  |  |  | 42.3 Dilatación del VI | | | | | | | | | | | | | | | | | | | | | | | □ Sí | | | | | | | | | □ No | | | | | | | | □ Ignorado | | |
|  |  |  |  |  |  |  |  |  |  | 42.4 Función sistólica del VI | | | | | | | | | | | | | | | | | | | | | | | □ Normal | | | | | | | | | □ Anormal | | | | | | | | □ Ignorado | | |
|  |  |  |  |  |  |  |  |  |  | 42.4.1 Si anormal, | | | | | | | | | | | | | | | | | | | | | | | □ Leve | | | | | | | | | □ Moderada | | | | | | | | □ Grave | | |
|  |  |  |  |  |  |  |  |  |  | 42.5 Función sistólica del VD | | | | | | | | | | | | | | | | | | | | | | | □ Normal | | | | | | | | | □ Anormal | | | | | | | | □ Ignorado | | |
|  |  |  |  |  |  |  |  |  |  | 42.6 Aneurisma apical | | | | | | | | | | | | | | | | | | | | | | | □ Sí | | | | | | | | | □ No | | | | | | | | □ Ignorado | | |
|  |  |  |  |  |  |  |  |  |  | 42.7 Trombo ventricular izquierdo | | | | | | | | | | | | | | | | | | | | | | | □ Sí | | | | | | | | | □ No | | | | | | | | □ Ignorado | | |
|  |  |  |  |  |  |  |  |  |  | 42.8 Presencia de alteración valvular | | | | | | | | | | | | | | | | | | | | | | | □ Sí | | | | | | | | | □ No | | | | | | | | □ Ignorado | | |
|  |  |  |  |  |  |  |  |  |  | 42.8.1 En caso confirmativo ¿es asociado a EChagas? | | | | | | | | | | | | | | | | | | | | | | | | | | | | | | | | □ Sí | | | | | | | | □ No | | |
|  |  |  |  |  |  |  |  |  |  | 42.8.2 ¿Cuál es la alteración? | | | | | | | | | | | | | | | | | | | | | | | □ Insuficiencia mitral_□ Insuficiencia tricuspídea | | | | | | | | | | | | | | | | | | | |
|  |  |  |  |  |  |  |  |  |  | 42.8.3 Grado de insuficiencia | | | | | | | | | | | | | | | | | | | | | | | □ Leve | | | | | | | | | □ Moderada | | | | | | | | □ Grave | | |
|  |  |  |  |  |  |  |  |  |  | 42.9 Disfunción diastólica | | | | | | | | | | | | | | | | | | | | | | | □ Sí | | | | | | | | | □ No | | | | | | | | □ Ignorado | | |
|  |  |  |  |  |  |  |  |  |  | 42.9.1 Si se observa disfunción diastólica, | | | | | | | | | | | | | | | | | | | | | | | □ Tipo I | | | | | | | | | □ Tipo II | | | | | | | | □ Tipo III | | |
|  |  |  |  |  |  |  |  |  |  | 42.10 Cociente E/e’ \|__\|__\|.\|\|__\| | | | | | | | | | | | | | | | | | | | | | | |  | | | | | | | | |  | | | | | | | |  | | |
|  |  |  |  |  |  |  |  |  |  | 42.11 Volumen auricular izquierdo | | | | | | | | | | | | | | | | | | | | | | | \|__\|__\|.\|\|__\|mL/m^2^ | | | | | | | | |  | | | | | | | |  | | |
|  |  |  |  |  |  |  |  |  |  | 42.12 Hipertensión pulmonar | | | | | | | | | | | | | | | | | | | | | | | □ Sí | | | | | | | | | □ No | | | | | | | | \|__\|__\|__\|mmHg | | |
|  |  |  |  |  |  |  |  |  |  | 42.13 Otros hallazgos | | | | | | | | | | | | | | | | | | | | | | | □ Sí | | | | | | | | | □ No | | | | | | | |  | | |
|  |  |  |  |  |  |  |  |  |  | 42.13.1 En caso afirmativo, especificar | | | | | | | | | | | | | | | | | | | | | | |  | | | | | | | | | | | | | | | | | | | |
| 43. Radiografía de tórax | | | | | | | | | | □ No realizado | | | | | | | | | | | | | | | | | | | | | | | | | | | | □ Realizado | | | | | | | | | | | | | | |
| 43.1 Fecha de realización de la radiografía de tórax | | | | | | | | | | | | | | | | \|__\|__\| - \|__\|__\|__\| - \|__\|__\|__\|__\| | | | | | | | | | | | | | | | | | | | | | | | | | | | | | | | | | | | | |
| 43.2. Cardiomegalia | | | | | | | | | | □ No  □ Sí  Índice cardiotorácico_________________ | | | | | | | | | | | | | | | | | | | | | | | | | | | | | | | | | | | | | | | | | | |
| 44. Monitorización de 24 horas con Holter | | | | | | | | | | □ No realizada □ Realizada | | | | | | | | | | | | | | | | | | | | | | | | | | | | | | | | | | | | | | | | | | |
| 44.1 Fecha del Holter | | | | | | | | | | \|__\|__\| - \|__\|__\|__\| - \|__\|__\|__\|__\|  44.1.1 Taquicardia ventricular sostenida □ Sí □ No  44.1.2 Taquicardia ventricular no sostenida □ Sí □ No  44.1.3 Fibrilación auricular □ Sí □ No  44.1.4 Bradicardia < 40 lpm □ Sí □ No  44.1.5 Pausa sinusal > 3 segundos □ Sí □ No | | | | | | | | | | | | | | | | | | | | | | | | | | | | | | | | | | | | | | | | | | |
| 45. RMN cardíaca □ Realizada □ No realizada  45.1 Fecha de la RMN cardíaca \|__\|__\| - \|__\|__\|__\| - \|__\|__\|__\|__\|  45.1.1 Presencia de fibrosis cardíaca □ Sí □ No □ Ignorado  45.1.2 Masa fibrótica cardíaca \|__\|__\|__\| (g)  45.1.3 Aneurisma de VI □ Sí □ No □ Ignorado  45.1.4 Trombo de VI □ Sí □ No □ Ignorado | | | | | | | | | | | | | | | | | | | | | | | | | | | | | | | | | | | | | | | | | | | | | | | | | | | | |
| 46. Medición de BNP y/o NT-proBNP □ No realizada □ Realizada  46.1 Fecha de BNP y/o proBNP \|__\|__\| - \|__\|__\|__\| - \|__\|__\|__\|__\|  46.1.1 BNP □ Normal □ Anormal \|__\|__\|__\|__\| (pg/mL) □ Ignorado  46.1.2NT-pro BNP □ Normal □ Anormal \|__\|__\|__\|__\| (pg/mL) □ Ignorado | | | | | | | | | | | | | | | | | | | | | | | | | | | | | | | | | | | | | | | | | | | | | | | | | | | | |
| 1. **Clasificaciones** | | | | | | | | | | | | | | | | | | | | | | | | | | | | | | | | | | | | | | | | | | | | | | | | | | | | |
| 47. Clasificación Kuschnir | | | | | | | | | | | |  | | | | | | | □ 0 | | | | | | | | | | | □ 1 | | | | | | | □ 2 | | | | | | | | □ 3 | | | | | | | □ Ignorado |
| 48. Clasificación Consenso Brasileño | | | | | | | | | | | | □ FI | | | | | | | □ A | | | | | | | | | | | □ B1 | | | | | | | □ B2 | | | | | | | | □ C  □ D | | | | | | | □ Ignorado |
| 49. Clasificación Latinoamericana | | | | | | | | | | | | □ A | | | | | | | □ B1 | | | | | | | | | | | □ B2 | | | | | | | □ C | | | | | | | | □ D | | | | | | | □ Ignorado |
| 50. Clasificación AHA | | | | | | | | | | | | □ A | | | | | | | □ B1 | | | | | | | | | | | □ B2 | | | | | | | □ C | | | | | | | | □ D | | | | | | | □ Ignorado |
| 51. Clasificación Los Andes | | | | | | | | | | | |  | | | | | | | □ IA | | | | | | | | | | | □ IB | | | | | | | □ II | | | | | | | | □ III | | | | | | | □ Ignorado |
| **Hallazgos digestivos** | | | | | | | | | | | | | | | | | | | | | | | | | | | | | | | | | | | | | | | | | | | | | | | | | | | | |
| 52. Patología digestiva detectada | | | □ Sí | | | | | | | | | | | | | | | | | | | | | | | □ No | | | | | | | | | | | | | | | | | | | □ No investigado | | | | | | | |
| En caso afirmativo, | | |  | | | | | | | | | | | | | | | | | | | | | | |  | | | | | | | | | | | | | | | | | | |  | | | | | | | |
| 52.1 Megacolon | | | □ Sí | | | | | | | | | | | | | | | | | | | | | | | □ No | | | | | | | | | | | | | | | | | | | □ No investigado | | | | | | | |
| 52.2 Megaesófago | | | □ Sí | | | | | | | | | | | | | | | | | | | | | | | □ No | | | | | | | | | | | | | | | | | | | □ No investigado | | | | | | | |
| 52.2.1 Clasificación Rezende | | | | | | | | | | | | □ E0 | | | | | | | □ EI | | | | | | | | | | | □ EII | | | | | | | □ EIII | | | | | | | | □ EIV | | | | | | | □ No realizado |
| **Clasificación de estado clínico** | | | | | | | | | | | | | | | | | | | | | | | | | | | | | | | | | | | | | | | | | | | | | | | | | | | | |
| 53. Forma clínica | | | | | | | | | | □ Control  □ Crónico  □ Agudo | | | | | | | | | | | | | | | 53.1 Si es crónico, especificar tipo:  □ Crónica sin patología demostrable (forma indeterminada)  □ Crónico con patología cardíaca  □ Crónico con patología digestiva  □ Crónico con patología mixta    53.2 Si es agudo, especificar tipo:  □ Primoinfección  □ Reactivación | | | | | | | | | | | | | | | | | | | | | | | | | | | |
| 1. **Tratamiento** | | | | | | | | | | | | | | | | | | | | | | | | | | | | | | | | | | | | | | | | | | | | | | | | | | | | |
| 54. ¿Tratamiento etiológico? | | | | | | | | | | □ Sí | | | | | | | | | | | | | | | | | | | | | | | | | | | | □ No | | | | | | | | | | | | | | |
| 54.1 En caso afirmativo, ¿cuál es el estatus del tratamiento? | | | | | | | | | | | | | | | | | | | | | | | | | | | | | | | | | | | □ Finalizado | | | | | | | | | □ En curso | | | | | | | | □ Interrumpido |
| Recoger los siguientes detalles una vez finalizado/interrumpido el tratamiento: | | | | | | | | | | | | | | | | | | | | | | | | | | | | | | | | | | | | | | | | | | | | | | | | | | | | |
| 54.2 Fármaco | | | | | | | | | | | | | | □ BNZ | | | | | | | | | | | | □ NFT | | | | | | | | | | □ Otro, ¿cuál?__________________________ | | | | | | | | | | | | | | | | |
| 54.3 Dosis total administrada | | | | | | | | | | | | | | | | | | | | | | | | | | \|__\|__\|__\|__\|__\| (mg) | | | | | | | | | | | | | | | | | | | | | | | | | | |
| 54.4 Días totales de tratamiento | | | | | | | | | | | | | | | | | | | | | | | | | | \|__\|__\|__\| | | | | | | | | | | | | | | | | | | | | | | | | | | |
| 54.5 Fecha de inicio | | | | | | | | | | | | | | | | | | | | | | | | | | \|__\|__\| - \|__\|__\|__\| - \|__\|__\|__\|__\| | | | | | | | | | | | | | | | | | | | | | | | | | | |
| 54.6 Fecha de fin | | | | | | | | | | | | | | | | | | | | | | | | | | \|__\|__\| - \|__\|__\|__\| - \|__\|__\|__\|__\| | | | | | | | | | | | | | | | | | | | | | | | | | | |
| 54.7 En caso de tratamiento interrumpido, causa: _________________________________________  ___________________________________________________________________________________________________ | | | | | | | | | | | | | | | | | | | | | | | | | | | | | | | | | | | | | | | | | | | | | | | | | | | | |
| 54.8 Efectos secundarios asociados a tratamiento etiológico | | | | | | | | | | | | | | | | | | | | | | | | | | | | | □ Sí | | | | | | | | | | | | | | | □ No | | | | | | | | |
| 54.8.1 ¿Cuál o cuáles? _________________________________________________________________________________ | | | | | | | | | | | | | | | | | | | | | | | | | | | | | | | | | | | | | | | | | | | | | | | | | | | | |
| 55. ¿Tratamiento cardiovascular adiciona? | | | | | | | | | | | | | | | | | | | | | | | □ Sí | | | | | | | | | | | | | | | □ No | | | | | | | | | | | | | | |
| 55.1 En caso afirmativo, especificar  □ Beta-bloqueante □ Inhibidor de la enzima convertidora de angiotensina (IECA) □ Bloq. Receptor de angiotensina  □ Espironolactona □ Sacubitril/valsartan □ Inhibidor de SGLT2 □ Ivabradine □ Furosemida  □ Digoxina □ Anticoagulantes orales □ Amiodarona | | | | | | | | | | | | | | | | | | | | | | | | | | | | | | | | | | | | | | | | | | | | | | | | | | | | |
| 1. **Muestras biológicas** | | | | | | | | | | | | | | | | | | | | | | | | | | | | | | | | | | | | | | | | | | | | | | | | | | | | |
| 56. ¿Se han recogido muestras biológicas? | | | | | | | | | | | | | | | | | | | | | | | □ Sí | | | | | | | | | | | | | | | □ No | | | | | | | | | | | | | | |
| **Tipo de muestra** Suero/Plasma/Sangre total/Orina/Saliva/ ADN de muestra de tejido/ Células de sangre periférica/ Otras, ¿cuál? | | **Número de alícuotas** | | | | | | | | | | | **Volumen de muestra** | | | | | | | | | | | | | | | **Cód. id. muestra** | | | | | | | | | | **Fecha de recogida de la muestra** | | | | | | | | | | | | | | |
|  | |  | | | | | | | | | | |  | | | | | | | | | | | | | | |  | | | | | | | | | | \|__\|__\| - \|__\|__\|__\| - \|__\|__\|__\|__\| | | | | | | | | | | | | | | |
|  | |  | | | | | | | | | | |  | | | | | | | | | | | | | | |  | | | | | | | | | | \|__\|__\| - \|__\|__\|__\| - \|__\|__\|__\|__\| | | | | | | | | | | | | | | |
|  | |  | | | | | | | | | | |  | | | | | | | | | | | | | | |  | | | | | | | | | | \|__\|__\| - \|__\|__\|__\| - \|__\|__\|__\|__\| | | | | | | | | | | | | | | |
|  | |  | | | | | | | | | | |  | | | | | | | | | | | | | | |  | | | | | | | | | | \|__\|__\| - \|__\|__\|__\| - \|__\|__\|__\|__\| | | | | | | | | | | | | | | |
|  | |  | | | | | | | | | | |  | | | | | | | | | | | | | | |  | | | | | | | | | | \|__\|__\| - \|__\|__\|__\| - \|__\|__\|__\|__\| | | | | | | | | | | | | | | |

BNP, péptido natriurético cerebral; BNZ, benznidazol, EPOC, Enfermedad pulmonar obstructiva crónica; CRT-D, Terapia de resincronización cardíaca con desfibrilador; CRT-P, Terapia de resincronización cardíaca con marcapasos; E, Velocidad de llenado diastólica temprana pico; e`, Velocidad diastólica temprana en el anillo mitral; ICD, Dispositivo desfibrilador- cardioversor implantable; RMN: Resonancia magnética nuclear; NFT: Nifurtimox; VD: Ventrículo derecho; VI: Ventrículo izquierdo.
