# Supplementary material for: A standardized clinical database for research in Chagas disease: The NHEPACHA network
Source: PLoS Negl Trop Dis. 2024 Aug 15;18(8):e0012364. doi: 10.1371/journal.pntd.0012364 (PMC11326575; doi:10.1371/journal.pntd.0012364)
Supplement: S5 File — (DOCX) [file pntd.0012364.s005.docx]

**Instrucciones generales**

En el cuestionario clínico de pacientes con enfermedad de Chagas se solicitan datos puntuales que resumen la información detallada de una historia clínica completa. La presente guía representa un complemento para ayudar con la captura de dichos datos, tanto a través de la plataforma REDCap, como en el cuestionario físico.

En REDCap, algunas preguntas permiten marcar múltiples opciones. Estas están marcadas con una casilla de selección cuadrada. El resto de las preguntas admiten una única respuesta y están marcadas con una casilla circular. Si no se dispone de suficiente información para responder alguna pregunta (por ejemplo, si algún examen paraclínico no ha sido realizado), debe marcarse la opción: “ignorado”. En todos los casos, tenga en cuenta que debe evitarse el uso de abreviaturas al rellenar el cuestionario.

El cuestionario debe llenarse con la información del estado de salud actual del paciente al momento de recolectar los datos y las muestras. Cuando se solicite información de laboratorio y estudios de imagen, idealmente deben reportarse aquellos obtenidos dentro de los últimos 6 meses.

Dentro del cuestionario digital en REDCap, algunos campos tendrán que ser llenados en cada visita/revisión/seguimiento que tenga programada el paciente y se identifican con la leyenda “**Campo requerido**”. Otros campos solo serán requeridos la primera vez que se llene el cuestionario, y van acompañados de la leyenda “**Se recoge solo una vez”**. Si alguno de estos apartados debe ser editado, esto será posible al seleccionar la opción “vista previa”, seguido de “caso actual”. Esto permitirá ver y editar los datos ya recolectados.

A continuación, se presentan instrucciones para la recolección de algunas preguntas de particular importancia. Estas se organizan por número de apartado y pregunta. Si llegase a tener problemas o dudas con alguna de las preguntas no listadas a continuación, puede hacerlas haciendo clic en el símbolo , ubicado sobre la pregunta en el cuestionario REDCap. En caso de usar el cuestionario físico, por favor comuníquese directamente con alguno de los autores.

**Apartado I: Datos de la visita.**

Los tres datos que se recaban en este apartado deben recolectarse en cada visita.

Pregunta 1. El Id del paciente lo genera cada institución y representa el número con el cual identificará al paciente, por lo cual debe coincidir en todas las visitas del mismo paciente. El prefijo NEPACHA es el nombre con el que se identifica a la institución que forma parte de la red.

Pregunta 2. La fecha de visita se refiere al momento en el cual se cita al paciente para que sea revisado. En el cuestionario físico debe apuntarse el día, las tres primeras letras del mes y el año completo. Por ejemplo: 19-sep-2022.

**Apartado III. Datos del paciente.**

Si bien no se especifica en el cuestionario, ni es posible en la plataforma REDCap, se recomienda que en este punto el entrevistador solicite el número de teléfono del paciente o algún contacto cercano, así como un correo electrónico, a fin de garantizar comunicarse con el paciente en caso de ser necesario.

**Apartado IV: Información epidemiológica**.

Pregunta 11: Cuando se solicita el país de origen del paciente, se pretende determinar si procede de alguna zona endémica de enfermedad de Chagas.

Pregunta 12: Si el paciente no procede de ningún país endémico, se solicita el país de origen de la madre, para evaluar la posibilidad de transmisión vertical de la infección. En REDCap, esto ocurre automáticamente, sin embargo, si está usando el formato físico, el entrevistador debe tener en cuenta que la pregunta 12 solo se contesta si el paciente procede de una zona no endémica.

Pregunta 13: Hace referencia al posible mecanismo de transmisión. Es difícil establecer el mecanismo de transmisión con certeza, pero se sugiere indagar lo máximo posible. A continuación, se presentan una serie de preguntas que pueden guiar este proceso:

*Para indagar sobre mecanismo vectorial se sugiere realizar las siguientes preguntas:*

- ¿Conoce el vector (chinche/vinchuca/barbeiro/pito/chipo/pik)? Se recomienda mostrar una imagen con varios insectos donde incluya alguna de las especies que se encuentren en la zona de residencia o país de origen.
- ¿Recuerda la presencia del vector (decir el nombre con el que el participante conoce al vector) dentro de la casa o vivienda?
- ¿Recuerda haber visto el vector en el sitio de trabajo?
- ¿Recuerda haber tenido una roncha asociada a la picadura del vector?
- ¿Ha visto al vector mientras acampaba o se encontraba en el campo?

*Para indagar sobre mecanismo oral se sugiere realizar las siguientes preguntas:*

- ¿Tiene hábito de consumir pulpa de açaí, caña de azúcar o jugo de goiaba, guayaba u otra fruta tropical?

*Para indagar sobre mecanismo vertical se sugiere realizar las siguientes preguntas:*

- ¿Sabe si su abuela materna, madre o hermanos han sido diagnosticados con enfermedad de Chagas?

*Para indagar sobre mecanismo de accidente laboral se sugiere realizar la siguiente pregunta:*

- ¿A qué se dedica? Además de los trabajadores de salud/investigadores que manipulan muestras, considerar como riesgo los cazadores que pernoctan en el monte, los que manipulan carnes y sangre de animales para consumo humano no regulado.

*Para los mecanismos transfusional y trasplante se sugiere realizar las siguientes preguntas:*

- ¿Ha recibido una transfusión sanguínea? ¿Por qué motivo? ¿Fecha en la que se la pusieron?
- ¿Ha recibido un trasplante? ¿Por qué motivo? ¿Fecha en la que lo realizaron?

Pregunta 24: Los acrónimos de los dispositivos cardíacos se describen al final del cuestionario. La persona que llene el cuestionario deberá revisar la información proporcionada en la tarjeta del dispositivo, en posesión del paciente; así como en su historia clínica, con la finalidad de obtener cualquier tipo de información complementaria.

**Apartado V: Diagnóstico etiológico.**

En este apartado debe indicar todas las pruebas realizadas al paciente para alcanzar el diagnóstico etiológico. Se incluyen pruebas parasitológicas, serológicas y moleculares. En el caso de las pruebas serológicas, al menos dos pruebas positivas son necesarias para establecer el diagnóstico. El cuestionario permite ingresar información de hasta tres pruebas serológicas y dos moleculares. En las pruebas serológicas, el valor de cut off o punto de corte se refiere al valor sobre el cual se considera positivo el resultado de una prueba.

**Apartado VI: Cuadro clínico.**

Pregunta 32: Esta pregunta incluye la evaluación de la presencia de signos clínicos de insuficiencia cardíaca según la escala de la New York Heart Association (NYHA), presentada a continuación.

| Clasificación funcional de la severidad de la disnea según la New York Heart Association (NYHA) | |
| --- | --- |
| Grado I | El paciente presenta disnea al realizar grandes esfuerzos como correr, subir varios pisos de escalera o deporte intenso, que previamente podía realizar sin molestias. |
| Grado II | El paciente presenta disnea al realizar esfuerzos moderados como caminar, correr una distancia corta o subir un piso de escalera. |
| Grado III | El paciente presenta disnea al realizar esfuerzos leves como peinarse, vestirse, hablar o comer. |
| Grado IV | El paciente presenta disnea durante el reposo físico. |

Pregunta 37. El cuestionario de la plataforma REDCap solo desglosará la captura de signos vitales si se ha marcado la opción “Tomados” en esta pregunta.

**Apartado VII: Resultados de pruebas diagnósticas.**

Preguntas 39 y 40: Estas preguntas hacen referencia a la presencia de signos electrocardiográficos específicos de cardiomiopatía chagásica. Si el paciente presenta alguna alteración electrocardiográfica no listada en la pregunta 40 del cuestionario, seleccione la opción “Alteraciones inespecíficas” en la pregunta 39. Si el paciente presenta alguna alteración electrocardiográfica listada en la pregunta 40, por favor márquela en la casilla correspondiente.

Preguntas 41 a 46: Estas preguntas hacen referencia a los hallazgos observados en el ecocardiograma, radiografía de tórax, Holter, RMN cardíaca y valores séricos de BNP/NT-proBNP. Toda la información del participante debe recogerse, en el momento en que esté disponible.

Preguntas 41 y 42: Si el paciente presenta alguna alteración que no aparece entre las opciones de la pregunta 42, indicar la opción “presencia de alteraciones no Chagásicas” en la pregunta 41. Si el paciente tiene alguna alteración ecocardiográfica de las listadas en la pregunta 42, por favor seleccione la casilla correspondiente.

Para la pregunta 46, la siguiente tabla contiene los valores de BNP y NT-proBNP:

| **Escenario clínico** | **BNP** | **NT-proBNP** |
| --- | --- | --- |
| Pacientes no-agudos | < 35 pg/mL | < 125 pg/mL |
| Pacientes agudos | < 100 pg/mL | < 300 pg/mL |
| Vida media | 20 minutes | 120 minutes |

**Apartado VIII: Clasificaciones.**

A continuación, se presenta una tabla para ayudar a clasificar clínicamente a un paciente según las distintas clasificaciones incluidas en el apartado VIII.

Preguntas 47-51:

| Clasificaciones de enfermedad de Chagas de acuerdo a alteraciones en estudios de gabinete y síntomas | | | | | |
| --- | --- | --- | --- | --- | --- |
| Alteraciones en estudios de gabinete y síntomas | Clasificaciones | | | | |
|  | Kuschnir | Consenso Brasileño | Los Andes | Latinoamericana | AHA |
| ECG normal y aparentemente sin EEC | 0 | NA | IA | A | A^a^ |
| ECG normal con anomalías contráctiles | NA | NA | IB | NA | B1 |
| ECG anormal, aparentemente sin EEC | I | A | NA | B1 | B1 |
| ECG anormal, anomalías contráctiles con FEVI normal | NA | B1 | II | B1 | B1 |
| ECG anormal, probable EEC o FEVI anormal | II | B1 (FEVI ≥ 45%), B2 (FEVI ≤ 45%) | II | B2 | B2 |
| Insuficiencia cardiaca descompensada | III | C (compensado) | III | C (compensado) | C (compensado) |
| Insuficiencia cardiaca refractaria | NA | D (refractaria) | NA | D (refractaria) | D (refractaria) |
| ECC: electrocardiograma; EEC: enfermedad estructural cardiaca; FEVI: fracción de eyección del ventrículo izquierdo; NA: no aplica; AHA: Asociacion Americana del Corazón; a: sin alteración digestiva | | | | | |

Pregunta 52.2.1:

Clasificación Rezende para la acalasia de esófago

| Clasificación Rezende | |
| --- | --- |
| Grado 1 | Forma inicial, cuerpo esofágico con diámetro < 4 cm. |
| Grado 2 | Esófago dilatado con diámetro > 4 cm pero < 7 cm. |
| Grado 3 | Diámetro esofágico entre 7 y 10 cm. |
| Grado 4 | Diámetro > 10 cm, en el que puede observarse un eje sinuoso propio de un dolicoesófago. |

Pregunta 53: Los pacientes diagnosticados con infección por *Trypanosoma cruzi* deberán ser clasificados como agudos o crónicos, en función de su sintomatología y el tiempo desde la probable infección. En caso de que el paciente sea clasificado como crónico, deberá especificarse el tipo de daño orgánico detectado. En la plataforma REDCap, estas opciones solo se desplegarán si se selecciona la casilla “Crónico”.

En el caso de que el participante forme parte del grupo control de un estudio de investigación clínica, deberá marcarse la casilla correspondiente.

**Apartado IX: Tratamiento.**

Pregunta 54: Si el paciente ha recibido tratamiento etiológico con benznidazol o nifurtimox, o algún medicamento anti-parasitario en fase experimental en el contexto de un ensayo clñinico, deberá marcarse la casilla “Sí”. Las preguntas 53.1-53.8 solo deberán contestarse en este caso.

La pregunta 54.1 hace referencia al estado actual del tratamiento, y a si el paciente finalizó el esquema completo de tratamiento, lo interrumpió antes de finalizar, o se encuentra recibiéndolo aún (En curso). En la plataforma REDCap, las preguntas 54.1-54.8 sólo se desplegarán si se ha marcado “Sí” en la pregunta 54.

Pregunta 55.1.Incluir cualquier medicamento cardiovascular que el paciente este tomando al momento de la visita..

**Apartado X: Muestras biológicas**

Pregunta 56: En esta pregunta se debe indicar si se han obtenido muestras biológicas del paciente. En caso afirmativo, debe completarse la tabla correspondiente. En la plataforma REDCap se desplegará dicha tabla solo si se ha marcado “Si” en la pregunta 55. En este punto se solicitará información sobre el tipo de muestra, número de alícuotas, volumen, identificador y fecha de recogida de la muestra. Para el llenado de esta sección, se recomienda solicitar apoyo del encargado de recolectar las muestras.

**Glosario**

BNP, péptido natriurético cerebral

BNZ, benznidazol.

CRT-D, Terapia de resincronización cardíaca con desfibrilador.

CRT-P, Terapia de resincronización cardíaca con marcapaso.

E, Velocidad de llenado diastólica temprana pico.

E’, Velocidad diastólica temprana en el anillo mitral.

EPOC, Enfermedad pulmonar obstructiva crónica.

ICD, Dispositivo desfibrilador- cardioversor implantable.

NFT, nifurtimox.

RMN: Resonancia magnética nuclear

VD, Ventrículo derecho.

VI, Ventrículo izquierdo.
