# Supplementary material for: A standardized clinical database for research in Chagas disease: The NHEPACHA network
Source: PLoS Negl Trop Dis. 2024 Aug 15;18(8):e0012364. doi: 10.1371/journal.pntd.0012364 (PMC11326575; doi:10.1371/journal.pntd.0012364)
Supplement: S6 File — (DOCX) [file pntd.0012364.s006.docx]

**Título: Um banco de dados clínico padronizado para pesquisa na doença de Chagas: Rede NHEPACHA**

**Autores**: Adriana González Martínez^1,2^, Irene Losada Galván^3,4^, Juan Carlos Gabaldón-Figueira^3^, Nieves Martínez-Peinado^3,5^, Roberto Magalhães Saraiva^6^, Marisa Liliana Fernández^7^, Janine M Ramsey^8^, Oscar Noya-González^9,10, 11^, Belkisyole Alarcón de Noya^10^, Alejandro Gabriel Schijman^12^, Soledad Berón^13^, Marcelo Abril^13^, Joaquim Gascón^3,14^, Sergio Sosa-Estani^15,16^, María Jesús Pinazo^15^, Julio Alonso-Padilla^3,14^*, Alejandro Marcel Hasslocher-Moreno^6^* representando o grupo de estudo da rede NHEPACHA**

Autor correspondente*

Alejandro Marcel Hasslocher-Moreno

Email: [alejandro.hasslocher@gmail.com](mailto:alejandro.hasslocher@gmail.com)

Julio Alonso-Padilla

Email: [julio.a.padilla@isglobal.org](mailto:julio.a.padilla@isglobal.org)

**A lista completa dos membros da rede NHEPACHA é apresentada na seção de agradecimentos

**Afiliações:**

^1^Departamento de Investigación, Salvando Latidos A.C., Guadalajara, Mexico.

^2^Departamento de Investigación, Instituto Cardiovascular de Mínima Invasión (ICMI), Guadalajara, Mexico.

^3^Barcelona Institute for Global Health (ISGlobal), Hospital Clínic - Universitat de Barcelona, Barcelona, Spain.

^4^Hospital Universitario 12 de Octubre, Madrid, Spain.

^5^Secció de Parasitologia, Departament de Biologia, Sanitat i Medi Ambient, Facultat de Farmàcia
i Ciències de l'Alimentació, Universitat de Barcelona, 08007 Barcelona, Spain.

^6^Evandro Chagas National Institute of Infectious Diseases, Fundação Oswaldo Cruz, Rio de Janeiro, Brasil.

^7^Instituto Nacional de Parasitología Dr M. Fatala Chabén, Administración Nacional de Laboratorios e Institutos de Salud Dr C. Malbrán, Ministerio de Salud, Buenos Aires, Argentina.

^8^Centro Regional de Investigación en Salud Pública (CRISP), Instituto Nacional de Salud Pública (INSP), Tapachula, Chiapas, México.

^9^Cátedra de Parasitología, Escuela ¨Luís Razetti” Facultad de Medicina, Universidad Central de Venezuela, Caracas, Venezuela.

^10^Instituto de Medicina Tropical, Facultad de Medicina, Universidad Central de Venezuela, Caracas, Venezuela.

^11^Centro para Estudios Sobre Malaria, Instituto de Altos Estudios “Dr. Arnoldo Gabaldón”, Ministerio del Poder Popular para la Salud (MPPS), Caracas, Venezuela

^12^Laboratorio de Biología Molecular de la Enfermedad de Chagas, Instituto de Investigaciones en Ingeniería Genética y Biología Molecular “Dr. Héctor N. Torres” - INGEBI-CONICET, Buenos Aires, Argentina.

^13^Fundación Mundo Sano, Buenos Aires, Argentina.

^14^CIBER de Enfermedades Infecciosas, Instituto de Salud Carlos III (CIBERINFEC, ISCIII), Madrid, Spain.

^15^Drugs for Neglected Diseases Initiative (DNDi), Geneva, Switzerland.

^16^Centro de Investigaciones Epidemiológicas y Salud Pública, Consejo Nacional de Investigaciones Científicas y Técnicas, Buenos Aires, Argentina.

**Resumo**

A Rede Ibero-Americana NHEPACHA, fundada por iniciativa de um grupo de pesquisadores de países latino-americanos e da Espanha, tem como objetivo estabelecer um marco de pesquisa para a doença de Chagas que abranja diagnóstico e tratamento. Para isso, a rede elaborou um questionário para reunir dados relevantes sobre aspectos epidemiológicos, clínicos, diagnósticos e terapêuticos da doença. Esse questionário foi desenvolvido com base em um consenso dos membros especialistas da rede, com a intenção de coletar dados padronizados de alta qualidade, os quais podem ser usados de forma intercambiável pelos diferentes centros de pesquisa que compõem a rede NHEPACHA. Além disso, a rede pretende oferecer um protocolo clínico que possa ser adotado por outros pesquisadores, facilitando a comparabilidade entre estudos publicados, bem como o desenvolvimento de marcadores de resposta terapêutica e progressão.

**Palavras-chave:** doença de Chagas; banco de dados clínicos; rede

**Introdução**

A Organização Mundial da Saúde inclui a doença de Chagas (CD), causada pelo protozoário *Trypanosoma cruz*i (*T. cruzi*), como uma das vinte doenças tropicais negligenciadas [1]. Surgindo originalmente de regiões endêmicas na América, principalmente América Latina, o impacto da CD se expandiu para o hemisfério norte e atualmente representa um problema de saúde pública nos Estados Unidos, Europa e Japão [2]. A presença da doença em países desenvolvidos não endêmicos motivou o envolvimento de diversas instituições governamentais de saúde e centros de pesquisa nos esforços de controle, diagnóstico e tratamento [3,4].

A doença de Chagas (CD) tem duas fases clínicas bem definidas: a fase aguda, que ocorre logo após a infecção, e a fase crônica, que se estende ao longo da vida dos indivíduos infectados. Na fase crônica, aproximadamente dois terços dos indivíduos não apresentam nenhuma doença clínica aparente, ou sinais detectáveis de danos nos órgãos. Os indivíduos restantes podem manifestar danos nos tecidos cardíaco e/ou digestivo, levando à disfunção. Assim, a fase crônica é categorizada em formas clínicas chamadas de indeterminada, cardíaca, digestiva e mista [5]. A fase crônica é dinâmica, o que significa que os pacientes podem progredir da forma indeterminada para as formas com envolvimento de órgãos, a uma taxa média anual estimada de 1,9% [6]. Da mesma forma, os indivíduos também podem progredir entre diferentes estágios clínicos de doença cardíaca e digestiva [7,8]. Uma das principais limitações para o manejo e propedêutica de pessoas na fase indeterminada ou na fase sintomática da DC é a ausência de marcadores de progressão para avaliar a evolução clínica [9].

Quanto ao tratamento etiológico da DC, as diretrizes recomendam inequivocamente o tratamento obrigatório durante a fase aguda, incluindo a reativação de infecções crônicas, casos congênitos e em crianças e adolescentes. Da mesma forma, elas recomendam fortemente o tratamento de mulheres em idade fértil e de adultos infectados com *T. cruzi* até os 50 anos de idade, com função miocárdica normal [10]. Atualmente, vários estudos têm mostrado que o uso de medicamentos tripanocidas pode alterar a história natural da DC, reduzindo o risco de progressão da doença [11-13], bem como reduzir a carga parasitária pelo menos até três anos após o tratamento, diminuindo assim a fonte potencial de infecção e o risco de transmissão [14].

A avaliação médica é essencial para caracterizar clinicamente a infecção por *T. cruzi* e o estágio da doença de Chagas, determinando o grau de envolvimento cardíaco e/ou digestivo, estratificando riscos e orientando o tratamento do paciente. A evidência clínica é crucial para orientar os médicos nesse processo, ajudando-os a escolher as abordagens diagnósticas e terapêuticas mais eficazes. É importante observar que a evidência médica é dinâmica e está em constante evolução à medida que novas pesquisas são conduzidas [15].

A pesquisa clínica na DC teve início na década de 1920 com o trabalho pioneiro de Carlos Chagas e Eurico Vilela. Na década de 1930, Evandro Chagas sistematizou aspectos clínicos e diagnósticos da cardiopatia chagásica, que eventualmente foi apresentada como uma característica definidora da doença para a comunidade científica internacional por Laranja na década de 1950 [16-18]. Desde então, inúmeros estudos sobre aspectos clínicos, diagnóstico e tratamento da DC foram publicados [19]. Levou mais de 50 anos para a maioria das diretrizes e manuais patrocinados pelo governo serem produzidos, já no século XXI, e apoiarem políticas públicas para enfrentar e controlar a doença [20]. Concomitantemente, durante esse período, os primeiros ensaios clínicos prospectivos randomizados surgiram para responder questões relacionadas ao prognóstico e tratamento da DC [21]. Esses ensaios destacaram a necessidade urgente de identificação de biomarcadores associados à eficácia terapêutica, com base em critérios parasitológicos, sorológicos ou clínicos [22]. Avanços nesse campo foram alcançados recentemente [23].

A padronização de dados epidemiológicos, diagnósticos, clínicos e terapêuticos para a doença de Chagas é essencial para instituições de referência que tratam pacientes com infecção por *T. cruzi* e para grupos de pesquisa que acompanham coortes em estudos transversais e longitudinais. Grandes bases de dados multicêntricas são fundamentais para a pesquisa médica, pois possibilitam que cientistas analisem volumes significativos de informações clínicas específicas e uniformes para testar hipóteses, avaliar a eficácia do tratamento, potencialmente prever a progressão e identificar tendências epidemiológicas. Um banco de dados clínicos para pesquisa é uma coleção organizada e estruturada de informações e dados relacionados a pacientes e suas condições médicas, coletados com o objetivo de conduzir pesquisas clínicas ou estudos epidemiológicos. Existem diferentes tipos de bancos de dados clínicos, incluindo bancos de dados de pesquisa clínica que coletam dados especificamente para fins de pesquisa, bancos de dados de registro de pacientes que registram informações do paciente em um ambiente clínico de rotina e bancos de dados de vigilância epidemiológica que monitoram a propagação de doenças [24].

A Rede Ibero-Americana “Nuevas Herramientas para el Diagnóstico y la Evaluación del Paciente con Enfermedad de Chagas” (NHEPACHA) foi criada pela iniciativa de um grupo de pesquisadores de DC de países latino-americanos e da Espanha em março de 2012. Atualmente, ela é composta por 12 grupos de pesquisa de 9 países. Seus objetivos são: (i) estabelecer um quadro de pesquisa, desenvolvimento e inovação para identificar potenciais novos medicamentos e biomarcadores para auxiliar no diagnóstico e no manejo da DC, bem como na avaliação de novos medicamentos em futuros ensaios clínicos; (ii) promover a troca de conhecimentos; e (iii) planejar estudos operacionais e ensaios clínicos multicêntricos para testar novas ferramentas e melhorar as existentes [25].

Alinhado a esse objetivo, a NHEPACHA agora disponibiliza um questionário clínico (material suplementaria 7) e um manual de preenchimento de questionário (material suplementaria 8) para a comunidade de pesquisa clínica da DC para facilitar a coleta de dados padronizados e comparáveis, com o objetivo de abordar questões específicas de pesquisa relacionadas à avaliação da eficácia do tratamento, identificação de fatores de risco ou avaliação de resultados clínicos.

**Materiais e métodos**

**Aspectos éticos**

A elaboração deste questionário não exigiu a obtenção de aprovação ética, pois não foram utilizados dados de pacientes em nenhuma etapa do processo. O uso deste questionário em pesquisa clínica deve ser realizado de acordo com os princípios da Declaração de Helsinque, em conformidade com a regulamentação local e após a aprovação por um comitê independente de ética em pesquisa.

**Metodologia**

A formulação do questionário foi alcançada por consenso entre especialistas que fazem parte do grupo NHEPACHA. Várias reuniões foram realizadas com o objetivo de criar um formulário de coleta de dados clínico-epidemiológicos que abordasse questões importantes de pesquisa na DC. As informações incluídas no formulário foram organizadas nas seguintes categorias: informações da visita, informações institucionais; informações do paciente; informações epidemiológicas; diagnóstico etiológico; apresentação clínica; informações paraclínicas; tratamento; e amostras biológicas. Uma vez que os itens mínimos e essenciais para o questionário foram definidos, esforços foram feitos para aderir a) às diretrizes internacionais e nacionais vigentes, incluindo um conjunto mínimo de informações-chave; e b) aos padrões estabelecidos que abrangiam aspectos importantes, como a privacidade e segurança dos pacientes sob avaliação de Comitês de Ética em Pesquisa (CEPs), coleta consistente de dados e padronização de dados para garantir qualidade.

O questionário clínico finalmente acordado foi transferido para formato eletrônico, utilizando o software de captura de dados eletrônicos REDCap (Research Electronic Data Capture) [26],[27]. O REDCap é uma plataforma de software baseada na web, projetada para apoiar a captura de dados em estudos de pesquisa, fornecendo: 1) uma interface intuitiva para a captura de dados validados; 2) trilhas de auditoria para rastrear manipulações de dados e procedimentos de exportação; 3) procedimentos automatizados de exportação para downloads contínuos de dados para pacotes estatísticos comuns; e 4) procedimentos para integração de dados e interoperabilidade com fontes externas. O REDCap é gratuito para organizações sem fins lucrativos que se associam ao Consórcio REDCap e é amplamente utilizado na comunidade acadêmica de pesquisa. O uso de um banco de dados centralizado baseado no REDCap hospedado no ISGlobal permitirá que os centros de pesquisa da NHEPACHA o utilizem de forma independente e colaborativa.

**Results**

*I. Informações da visita:* o paciente é identificado por código, juntamente com a data da visita.

*II. Informações institucionais:* incluem o nome do médico entrevistador, o nome da instituição participante, sua localização, e a data e número de aprovação do comitê de ética para o trabalho em questão.

*III. Informações do paciente:* data de nascimento e gênero do paciente.

*IV. Informações epidemiológicas:* o país de origem do paciente e o da mãe são identificados, se residem em área rural ou urbana, por quanto tempo estiveram longe da área endêmica original e se viveram em outros países. Na seção de histórico familiar, é indicado se há parentes com DC e o grau de parentesco com o paciente. Para pacientes do sexo feminino, é fornecido o histórico de gravidez, o número de filhos e informações sobre se as crianças foram examinadas para DC no primeiro ano de vida. O mecanismo de transmissão mais provável é indicado. Além disso, são observadas quaisquer comorbidades, coinfecções ou se o paciente está usando algum dispositivo.

*V. Diagnóstico:* é determinado se foram realizados testes parasitológicos, quais testes sorológicos foram realizados e se houve quantificação de títulos de anticorpos. Da mesma forma, se um teste molecular foi realizado, são fornecidas informações sobre a técnica específica utilizada e os resultados obtidos, se qualitativos ou quantitativos.

*VI. Apresentação clínica:* os sinais e sintomas do paciente são apresentados de acordo com a fase da doença. Para pacientes na forma crônica, são obtidas informações específicas sobre a forma clínica observada. São avaliados sinais de insuficiência cardíaca e envolvimento digestivo, e a classificação NYHA é usada para avaliar o estado funcional do paciente. Sinais associados à infecção congênita também são avaliados independentemente. Os dados do exame físico são anotados.

*VII. Resultado de exames:* é fornecida uma lista de verificação para avaliar a presença de alterações no eletrocardiograma, ecocardiograma transtorácico, raio-x de tórax, Holter de 24 horas, ressonância magnética do coração e BNP ou NT-proBNP para uma avaliação objetiva do envolvimento cardíaco do paciente.

*VII. Diagnostic test results:* a checklist to evaluate the presence of remarkable findings from the electrocardiogram, transthoracic echocardiogram, and chest X-ray are recorded for an objective assessment of cardiac involvement.

*VIII. Escalas clínicas:* a cardiopatia é estratificada usando classificações clínicas pré-estabelecidas da doença (“Modified Los Andes”, “Kuschnir”, “Brazilian consensus”, “I Latin American guidelines” e “American Heart Association Statement”) [5,28-31]. A forma digestiva é caracterizada, e a classificação de Rezende é usada para avaliar o grau de envolvimento esofágico [32]. É identificado se a fase aguda é uma infecção primária ou reativação da doença crônica. A forma crônica é caracterizada e dividida em indeterminada, cardíaca, digestiva ou mista.

*IX. Tratamento:* são fornecidos dados sobre o tratamento tripanocida recebido pelo paciente. A droga, data, dosagem e duração do tratamento são anotadas, assim como se o paciente apresentou algum evento adverso e se o tratamento foi interrompido. Perguntas sobre o uso de medicações cardiovasculares estão inclusas no questionário porque podem modificar o prognóstico de pacientes com forma crônica cardíaca.

*X. Amostras biológicas:* em todos os casos, foram coletadas amostras biológicas do paciente, e dados sobre seu tipo, volume e número de alíquotas são fornecidos. Um guia ad hoc e procedimentos operacionais padrão (POPs) sobre o gerenciamento de amostras no contexto da rede NHEPACHA estão sendo publicados em paralelo a este trabalho.

**Discussão**

Um processo padronizado de coleta de dados é essencial para obter dados de alta qualidade e comparáveis em estudos clínicos de DC, relacionados a amostras provenientes de pessoas infectadas por T. cruzi, pertencentes a diferentes contextos epidemiológicos. Um banco de dados de pesquisa clínica uniforme é de importância crítica para acelerar avanços no diagnóstico, evolução clínica e avaliação de tratamento desta doença negligenciada. Ele pode facilitar a colaboração entre diferentes equipes de pesquisa, simplificar o acesso aos dados, acelerar o ritmo geral da pesquisa e fornecer informações sobre diferenças regionais que podem resultar de características genéticas, imunológicas e proteômicas humanas e do parasita.

A ferramenta apresentada neste trabalho (em três idiomas: inglês, espanhol tem o potencial de proporcionar uma abordagem padronizada para a coleta de dados de pacientes. Esses dados comparáveis de diversos centros ao redor do mundo possibilitariam aos pesquisadores avaliar resultados clínicos e de biomarcadores de maneira diversificada e abrangente, acelerando o preenchimento das lacunas no conhecimento sobre a doença de Chagas para uma melhor atualização das diretrizes baseadas em evidências existentes e o desenvolvimento de novas.

É importante ressaltar que os dados derivados do protocolo clínico da rede NHEPACHA também permitirão que pesquisadores interessados tenham acesso a dados de pacientes cadastrados, ideais para conduzir estudos retrospectivos, gerar hipóteses ou orientar o desenvolvimento de ferramentas diagnósticas, prognósticas e/ou terapêuticas inovadoras, ao mesmo tempo facilitando o acompanhamento da progressão clínica de um paciente ao longo do tempo. Essa visão longitudinal, multicêntrica e internacional é essencial, dada a natureza crônica da doença de Chagas, e as lacunas significativas no conhecimento sobre a melhor maneira de avaliar sua progressão de longo prazo e resposta ao tratamento.

A natureza centralizada desse banco de dados também elimina barreiras para aquisição e processamento de dados, permitindo que pesquisadores de diversas instituições acessem rapidamente dados clínicos selecionados, sem passar pelo processo demorado de coleta e organização. Essa abordagem colaborativa estimula a pesquisa multidisciplinar, a reunião de expertise e a capacidade de lidar com questões de pesquisa complexas que exigem conjuntos de dados amplos e diversos.

Os pesquisadores podem evitar duplicar esforços na coleta de dados, já que os dados armazenados no banco de dados podem ser reutilizados em vários projetos de pesquisa. Essa eficiência pode contribuir para economizar tempo e recursos. Devido à sua natureza padronizada e ao fato de ser fruto de discussões de consenso de especialistas da rede NHEPACHA, outros pesquisadores poderão validar seus resultados ao compará-los com os de seus pares dentro da rede.

Em resumo, a padronização de bancos de dados clínicos para pesquisa é uma ferramenta essencial para a pesquisa médica moderna e desempenha um papel fundamental no avanço do desenvolvimento de novas ferramentas diagnósticas e terapêuticas. Nesse contexto, a ficha de registro de caso (“CRF”) da rede NHEPACHA e sua versão digitalizada hospedada no REDCap (eCRF) representam um recurso notável para a coleta e compartilhamento de dados clínicos padronizados e de alta qualidade. Esperamos que sua implementação permita aos pesquisadores abordar uma ampla gama de questões, desde a validação de métodos diagnósticos e avaliação da eficácia de medicamentos até a compreensão da dinâmica complexa da doença ao longo do tempo.

**Agradecimentos**

Gostaríamos de agradecer à DNDi e ao Mundo Sano pelo seu contínuo envolvimento no apoio à rede NHEPACHA.

**Grupo de estudo da Rede NHEPACHA:** Janine Ramsey W, Angelica Pech May, Alba Valdez Tah, Gilberto Sanchez Gonzalez, Adriana Gonzalez Martinez, Eduardo Ortiz Panozo, Mario J. Grijalva, Jaime A. Costales, Cesar A. Yumiseva, Carolina Herrera, Eileen Velez, Maria de Lourdes Torres, Maria Jesus Pinazo, Sergio Sosa Estani, Colin Forsyth, Eric Chatelain, Ivan Scandale, Fabiana Barreira, Tayná Marques, Marina Certo, Alejandro Hasslocher, Roberto Saraiva, Mauro Mediano, Andrea Silvestre, Sergio Xavier, Luiz Sangenis, Fernanda Mendes, Gilberto Sperandio da Silva, Andrea Costa, Henrique Veloso, Marcelo Holanda, Flavia Mazzoli, Paula Simplício da Silva, Tania Araujo, Mariana Wagabi, Luciana Garzoni, Constança Brito, Roberto Ferreira, Rita Machado, Raquel Aguiar, Marcelo Abril, Soledad Beron, Alejandro Schijman, Silvia Longhi, Arturo Muñoz-Calderón, Belkisyole Alarcon de Noya, Oscar Noya Gonzalez, Arturo Muñoz, Cecilia Colmenares, Ivan Mendoza, Zoraida Diaz, Raiza Ruiz, Ana Andreina Alviares, María Carmen Thomas, Manuel Carlos Lopez, Adriana Egui, Celia Benitez, Inmaculada Gómez, Francisco Macias Huete, Andres Mariano Ruiz, Rocio Rivero, Mónica Esteva, Margarita Bisio, Marisa Fernandez, Yolanda Hernandez, Julio Alonso Padilla, Joaquim Gascon, Irene Losada Galván, Nieves Martinez-Peinado, Juan Carlos Gabaldon-Figueira, María Gabriela Alvarez, Lococo Bruno, Laucella Susana, Flavio Andrés Tóman Conte, Dr. Enrique Morral, Maria Cecilia Albareda, Fernán Agüero, Emir Salas Sarduy, Alejandro Ricci, Leonel Bracco, Mercedes Didier Garnham, Alejandro Luquetti, Igor Almeida, Ester Sabino, Felipe Guhl, Faustino Torrico

**Referências**

1. WHO. Chagas disease (American trypanosomiasis). 2020 [cited 12 Jul 2020]. Available: https://www.who.int/chagas/disease/en/

- 2. Gascon J, Bern C, Pinazo M-J. Chagas disease in Spain, the United States and other non-endemic countries. Acta Trop. 2010. Jul; 115 (1-2): 22–27. PMID: 19646412
- 3. Requena-Méndez A, Albajar-Viñas P, Angheben A, Chiodini P, Gascón J, Muñoz J, et al. Health Policies to Control Chagas Disease Transmission in European Countries.. PLoS Negl Trop Dis. 2014. Oct; 8 (10):e3245. PMID: 25357193
- 4. Gonzalez-Sanz M, Crespillo-Andújar C, Chamorro-Tojeiro S, Monge-Maillo B, Perez-Molina JA, Norman FF. Chagas Disease in Europe. Tropical Med. 2023. Dec; 8 (12): 513. PMID: 38133445

5. Dias JCP, Ramos Jr. AN, Gontijo ED, Luquetti A, Shikanai-Yasuda MA, Coura JR, et al. 2^nd^ Brazilian Consensus on Chagas Disease, 2015. Rev Soc Bras Med Trop. 2016. Dec; 49 (Suppl 1): 3–60.

- 6. Chadalawada S, Sillau S, Archuleta S, Mundo W, Bandali M, Parra-Henao G, et al. Risk of Chronic Cardiomyopathy Among Patients With the Acute Phase or Indeterminate Form of Chagas Disease: A Systematic Review and Meta-analysis. JAMA Network Open. 2020. Aug; 3 (8): e2015072. PMID: **32865573**
- 7. Saraiva RM, Mediano MFF, Quintana MSB, Sperandio da Silva GM, Costa AR, Sousa AS, et al. Two-dimensional strain derived parameters provide independent predictors of progression to Chagas cardiomyopathy and mortality in patients with Chagas disease. IJC Heart & Vasculature. 2022. Jan;38:100955. PMID: **35169612**

8. Castro C, Prata A, Macedo V. A folow-up period of 13 years prospective study in 190 chagasic patients of Mambaí, Goiás, State, Brazil. Rev Soc Bras Med Trop. 2001. Jul; 34 (4): 309–318.

- 9. Cortes-Serra N, Losada-Galvan I, Pinazo M-J, Fernandez-Becerra C, Gascon J, Alonso-Padilla J. State-of-the-art in host-derived biomarkers of Chagas disease prognosis and early evaluation of anti-*Trypanosoma cruzi* treatment response. Biochimica et Biophysica Acta (BBA) - Molecular Basis of Disease. 2020. Jul; 1866 (7): 165758. PMID: **32169507**
- 10. Organización Panamericana de la Salud. Síntesis de evidencia: Guía para el diagnóstico y el tratamiento de la enfermedad de Chagas. Revista Panamericana de Salud Publica. 2020. Jun;44: e28. PMID: 32523605
- 11. Viotti R, Vigliano C, Lococo B, Bertocchi G, Petti M, Alvarez MG, et al. Long-term cardiac outcomes of treating chronic Chagas disease with benznidazole versus no treatment: a nonrandomized trial. Ann Intern Med. 2006. May;144(10): 724–734. PMID: 16702588

12. Fabbro DL, Streiger ML, Arias ED, Bizai ML, del Barco ML, Amicone NA. Trypanocide treatment among adults with chronic Chagas disease living in Santa Fe City (Argentina), over a mean follow-up of 21 years: parasitological, serological and clinical evolution. Rev Soc Bras Med Trop. 2007. Feb; 40: 1–10.

- 13. Hasslocher-Moreno AM, Saraiva RM, Sangenis LHC, Xavier SS, de Sousa AS, Costa AR, et al. Benznidazole decreases the risk of chronic Chagas disease progression and cardiovascular events: A long-term follow up study. EClinicalMedicine. 2021. Dec;31: 100694. PMID: 33554085
- 14. Torrico F, Gascón J, Barreira F, Blum B, Almeida IC, Alonso-Vega C, et al. New regimens of benznidazole monotherapy and in combination with fosravuconazole for treatment of Chagas disease (BENDITA): a phase 2, double-blind, randomised trial. The Lancet Infectious Diseases. 2021. Aug;21(8): 1129–1140. PMID: 33836161

15. Alves S, Silva B, Barbosa E, Medeiros C, Barros M, Cavalcanti M, et al. Advances in Clinical Practice, Diagnosis and Treatment of Chronic Chagas’ Heart Disease. ABC Heart Fail Cardiomyop. 2023. Apr; 3(1): e20230029.

16. Chagas C, Villela E. Forma cardíaca da trypanosomiase americana. Mem Inst Oswaldo Cruz. 1922;14: 5–61.

17. Chagas E. Novos estudos sobre a forma cardiaca da Trypanosomiase americana. Mem Inst Oswaldo Cruz. 1932;26(3): 329–338.

- 18. Laranja FS, Dias E, Nobrega G, Miranda A. Chagas’ disease A clinical, epidemiologic, and pathologic study. Circulation. 1956. Dec;14(6): 1035–1060. PMID: 13383798
- 19. Ramos JM, González-Alcaide G, Gascón J, Gutierrez F. Mapping of Chagas disease research: analysis of publications in the period between 1940 and 2009. Rev Soc Bras Med Trop. 2011. Dec;44(6): 708–716. PMID: 22094704
- 20. Levin LG, Kreimer PR, Jensen P. Chagas Disease across Contexts: Scientific Knowledge in a Globalized World. Medical Anthropology. 2021. Sep;40(6): 572–589. PMID: 34237229
- 21. Tarleton RL. Avoiding Clinical Trial Failures in Neglected Tropical Diseases: The Example of Chagas Disease. Clinical Infectious Diseases. 2023. Apr; 17(76): 1516–1520. PMID: 36373213

22. Hasslocher-Moreno AM, Sperandio-da-Silva GM, Saraiva RM. Trypanocidal Treatment in Chronic Chagas Disease: Critical Evaluation of Cure Criteria. Exploratory Research and Hypothesis in Medicine. [2024. Jun;9(2):175-177](https://www.xiahepublishing.com/m/journal/erhm/current).

- 23. Morales‐Velásquez M, Barón‐Vera JP, Osorio‐Pulgarín MI, Sánchez‐Jiménez MM, Ospina‐Villa JD. Biomarkers for the diagnosis, treatment follow‐up, and prediction of cardiac complications in Chagas disease in chronic phase: Recent advances. Parasite Immunology. 2023. Dec;45(12): e13013. PMID: 37795913
- 24. Lee JY. Uses of Clinical Databases. The American Journal of the Medical Sciences. 1994. Jul;308(1): 58–62. PMID: 8010340

25. NHEPACHA: New Tools for the Diagnosis and Evaluation of Chagas Disease. 2023 [cited 7 Oct 2023]. Available: https://www.isglobal.org/en/-/nuevas-herramientas-para-el-diagnostico-y-la-evaluacion-del-paciente-con-enfermedad-de-chagas-nhepacha

- 26. Harris PA, Taylor R, Thielke R, Payne J, Gonzalez N, Conde JG. Research electronic data capture (REDCap)—A metadata-driven methodology and workflow process for providing translational research informatics support. Journal of Biomedical Informatics. 2009. Apr;42(2): 377–381. PMID: 18929686
- 27. Harris PA, Taylor R, Minor BL, Elliott V, Fernandez M, O’Neal L, et al. The REDCap consortium: Building an international community of software platform partners. Journal of Biomedical Informatics. 2019. Jul;95: 103208. PMID: **31078660**
- 28. Carrasco HAG, Barboza JS, Inglessis G, Fuenmayor A, Molina C. Left ventricular cineangiography in Chagas’ disease: Detection of early myocardial damage. Am Heart J. 1982. Sep;104(3): 595–602. PMID: 7113900
- 29. Kuschnir E, Sgammini H, Castro R, Evequoz C, Ledesma R, Brunetto J. Evaluation of cardiac function by radioisotopic angiography, in patients with chronic Chagas cardiopathy. Arq Bras Cardiol. 1985. Oct;45(4): 249–256. PMID: 3835868
- 30. Andrade JP de, Marin Neto JA, Paola AAV de, Vilas-Boas F, Oliveira GMM, Bacal F, et al. I Latin American Guidelines for the Diagnosis and Treatment of Chagas’ Heart Disease. Executive Summary. Arq Bras Cardiol. 2011. Jun;96(6): 434–442. PMID: 21789345
- 31. Nunes MCP, Beaton A, Acquatella H, Bern C, Bolger AF, Echeverría LE, et al. Chagas Cardiomyopathy: An Update of Current Clinical Knowledge and Management: A Scientific Statement From the American Heart Association. Circulation. 2018. Sep;138(12).169-209. PMID: 30354432

32. Rezende JM. Classificação Radiológica do Megaesôfago. Rev Goiana Med. 1982. Jul;28(3-4): 187–191.

**Informações suplementares**

**Arquivo suplementar 1**: Questionário clínico de pacientes com Chagas (inglês).

**Arquivo suplementar 2**: Manual de preenchimento do questionário (inglês).

**Arquivo suplementar 3**: Um banco de dados clínicos padronizado para pesquisa em doença de Chagas: Rede NHEPACHA (espanhol).

**Arquivo suplementar 4**: Questionário clínico de pacientes com Chagas (espanhol).

**Arquivo suplementar 5**: Manual de preenchimento do questionário (espanhol).

**Arquivo suplementar 6**: Um banco de dados clínicos padronizado para pesquisa em doença de Chagas: Rede NHEPACHA (português).

**Arquivo suplementar 7**: Questionário clínico de paciente com Chagas (português).

**Arquivo suplementar 8**: Manual de preenchimento do questionário (português).
