# Supplementary material for: A standardized clinical database for research in Chagas disease: The NHEPACHA network
Source: PLoS Negl Trop Dis. 2024 Aug 15;18(8):e0012364. doi: 10.1371/journal.pntd.0012364 (PMC11326575; doi:10.1371/journal.pntd.0012364)
Supplement: S7 File — (DOCX) [file pntd.0012364.s007.docx]

| 1. **Informação da visita atual** | | | | | | | | | | | | | | | | | | | | | | | | | | | | | | | | | | | | | | | | | | | | | | | | | | | | | | | | | | | | | |
| --- | --- | --- | --- | --- | --- | --- | --- | --- | --- | --- | --- | --- | --- | --- | --- | --- | --- | --- | --- | --- | --- | --- | --- | --- | --- | --- | --- | --- | --- | --- | --- | --- | --- | --- | --- | --- | --- | --- | --- | --- | --- | --- | --- | --- | --- | --- | --- | --- | --- | --- | --- | --- | --- | --- | --- | --- | --- | --- | --- | --- | --- |
| 1. Número de identificação do paciente | | | | | | | | | | \|__\|__\|__\| -  Prefixo do centro NHEPACHA | | | | | | | | | | | | | | | | | | | | | | | | | | | | | | | | | ___________________________  Número de identificação do paciente na instituição de tratamento | | | | | | | | | | | | | | | | | | |
| 2. Data da visita | | | | | | | | | | \|__\|__\| - \|__\|__\|__\| - \|__\|__\|__\|__\| | | | | | | | | | | | | | | | | | | | | | | | | | | | | | | | | | | | | | | | | | | | | | | | | | | | |
| 1. **Informações institucionais** | | | | | | | | | | | | | | | | | | | | | | | | | | | | | | | | | | | | | | | | | | | | | | | | | | | | | | | | | | | | | |
| 3. Médico entrevistador | | | | | | | | __________________________________________________________________________ | | | | | | | | | | | | | | | | | | | | | | | | | | | | | | | | | | | | | | | | | | | | | | | | | | | | | |
| 4. Nome da instituição: | | | | | | | | | _________________________________________________________________________ | | | | | | | | | | | | | | | | | | | | | | | | | | | | | | | | | | | | | | | | | | | | | | | | | | | | |
| 5. Cidade/localização: | | | | | | | | _____________________ _____________________________________________________ | | | | | | | | | | | | | | | | | | | | | | | | | | | | | | | | | | | | | | | | | | | | | | | | | | | | | |
| 6. O consentimento informado foi obtido? | | | | | | | | | | | | | | | | | | | | | | | | | | | | | □ Sim | | | | | | | | | | | | | | | | □ Não | | | | | | | | | | | | | | | | |
| 7. Data de aprovação ética para o estudo | | | | | | | | | | | | | | | | | | | | | | | | | | | | | \|__\|__\| - \|__\|__\|__\| - \|__\|__\|__\|__\| | | | | | | | | | | | | | | | | | | | | | | | | | | | | | | | | |
| 8. Número de registro do comitê de aprovação ética: | | | | | | | | | | | | | | | | | | | | | | | | | | | | | __________________________________________ | | | | | | | | | | | | | | | | | | | | | | | | | | | | | | | | |
| 1. **Informações do paciente** | | | | | | | | | | | | | | | | | | | | | | | | | | | | | | | | | | | | | | | | | | | | | | | | | | | | | | | | | | | | | |
| 9. Data de nascimento | | | | | | | | | | | | | | | | | | | | | | | | | | | | | \|__\|__\| - \|__\|__\|__\| - \|__\|__\|__\|__\| | | | | | | | | | | | | | | | | | | | | | | | | | | | | | | | | |
| 10. Sexo biológico | | | | | | | | | | | | | | | | | | | | | | | | | | | | | □ Masculino | | | | | | | | | | | | | | | | □ Feminino | | | | | | | | | | | | | | | | |
| 1. **Informações epidemiológicas** | | | | | | | | | | | | | | | | | | | | | | | | | | | | | | | | | | | | | | | | | | | | | | | | | | | | | | | | | | | | | |
| 11. País de origem | | | | | □ Argentina  □ Belize  □ Bolívia  □ Brasil | | | | | | | | | | | | □ Chile  □ Colômbia  □ Costa Rica  □ Equador | | | | | | | | | | | | | | | | | | | □ El Salvador  □ Guatemala  □ Guiana  □ Honduras | | | | | | | | | | | | □ México  □ Nicarágua  □ Panamá  □ Paraguai | | | | | | | | | | | □ Peru  □ Suriname  □ Uruguai  □ Venezuela | | |
|  |  |  |  |  | □ Outro, especifique | | | | | | | | | | | | ___________________________________________________________ | | | | | | | | | | | | | | | | | | | | | | | | | | | | | | | | | | | | | | | | | | | | |
| 12. Se diferente do acima, indique o país de origem da mãe: | | | | | | | | | | | | | | | | | | | | | | | | | | | | | | | | | | | | ___________________________________________ | | | | | | | | | | | | | | | | | | | | | | | | | |
| 13. Possível mecanismo de transmissão | | | | | | | | | | | □ Desconhecido  □ Pós-transfusão | | | | | | | | | | | | | | | | | | | | | | | | | □ Oral  □ Transplante | | | | | | | | | | | | | | | | | | □ Vetorial  □ Vertical (Congênito) | | | | | | | |
|  |  |  |  |  |  |  |  |  |  |  | □ Acidente de laboratório | | | | | | | | | | | | | | | | | | | | | | | | | | | | | | | | | | | | | | | | | | | | | | | | | | |
| 14. Em caso de transfusão, transplante ou acidente, indique | | | | Localização:  _____________________ | | | | | | | | | | | | | | | | | | Razão:  _____________________ | | | | | | | | | | | | | | | | | | | | | | | | Data:  \|__\|__\| - \|__\|__\|__\| - \|__\|__\|__\|__\| | | | | | | | | | | | | | | | |
| 15. O paciente vive em: | | | | | | | | | | | | | | | | | | | | | | | | | | | | | □ Área rural | | | | | | | | | | | | | | | | | □ Área rural | | | | | | | | | | | | | | | |
| 16. Tempo vivendo fora de uma área endêmica: | | | | | | | | | | | | | | | | □  Ainda vive em área endêmica | | | | | | | | | | | | | | | | | | | | | | | | | | □ < 10 anos | | | | | | | | | | | | | | | □ > 10 anos | | | | |
| 17. Outros lugares onde o paciente viveu | | | | | | ____________________________________________________________________________ | | | | | | | | | | | | | | | | | | | | | | | | | | | | | | | | | | | | | | | | | | | | | | | | | | | | | | | |
| 18. História familiar de doença de Chagas? | | | | | | | | | | □ Mãe  □ Pai  □Outro, especifique: ____________________________ | | | | | | | | | | | | | | | | | | | | | | | | | | | | | | | | | □ Irmãos  □ Filhos | | | | | | | | | | | | | | | | | | |
| Se o paciente for de sexo feminino, responda às preguntas 19-21, caso contrário, pule para 22. | | | | | | | | | | | | | | | | | | | | | | | | | | | | | | | | | | | | | | | | | | | | | | | | | | | | | | | | | | | | | |
| 19. A paciente está grávida?  20. A paciente tem filhos?  21. As crianças foram testadas para doença de Chagas aos 8, 10 ou 12 meses após o nascimento?? | | | | | | | | | | □ Sim  □ Sim    □ Sim | | | | | | | | | | | | | | | | | | | | | | | | □ Não  □ Não    □ Não | | | | | | | | | □Desconhecido  □Desconhecido | | | | | | | | | | □ Ignorado  □ Ignorado | | | | | | | | |
| 22. O paciente apresenta alguma das seguintes comorbidades? | | | | | | | | | | □ Sem informação  □ Nenhum  □ Diabetes Mellitus  □ Hipotireoidismo  □ Hipertensão sistêmica  □ Outro, especifique | | | | | | | | | | | | | | | | | | | | | | | | □ Cardiomiopatia não chagásica  □ Doença hepática  □ Dislipidemia  □ Neoplasia  □ Transtornos neuropsiquiátricos  _______________________ | | | | | | | | | | | | | | | | | | | □ Doença autoimune  □ DPOC  □ Asma  □ História de doença tromboembólica | | | | | | | | |
| 23. O paciente apresenta alguma coinfecção? | | | | | | | | | | □ Sem informação  □ Outro, especifique | | | | | | | | | | | | | | | | | | | | | | | | □ VIH  _______________________ | | | | | | | | | | | | | | | | | | | □ Covid19 | | | | | | | | |
| 24. O paciente tem um dispositivo intracardíaco? | | | | | | | | | | □ Não | | | | | | | | | | | | | | | | | | | | | | | | □ Marcapasspo unicameral | | | | | | | | | | | | | | | | | | | □Marcapasspo bicameral | | | | | | | | |
|  | | | | | | | | | | □ ICD | | | | | | | | | | | | | | | | | | | | | | | | □ CRT-P | | | | | | | | | | | | | | | | | | | □ CRT-D | | | | | | | | |
| 1. **Diagnóstico etiológico** | | | | | | | | | | | | | | | | | | | | | | | | | | | | | | | | | | | | | | | | | | | | | | | | | | | | | | | | | | | | | |
| 25. Foi realizado um teste parasitológico? | | | | | | | | | | □ Sim  □ Não | | | | | | | | | | | | | | 25.1 Se sim, qual??   \| □ Strout  □ Micrométodo \| \| --- \| \| □ Xenodiagnóstico  □ Outro, qual? _____________________ \| | | | | | | | | | | | | | | | | | | | | | | | | | | | | | | | | | | | | | |
| 25.2 Data do teste parasitológico: | | | | | | | | | | | | | | | | | | | | | | | | | | \|__\|__\| - \|__\|__\|__\| - \|__\|__\|__\|__\| | | | | | | | | | | | | | | | | | | | | | | | | | | | | | | | | | | | |
| 25.3 Resultado | | | | | | | | | | □ Não detectável | | | | | | | | | | | | | | | | | | | | | | | | | | | | | | | | |  | | | | | | | | | | | | | | | | | | |
|  | | | | | | | | | | □ Detectável | | | | | | | | | | | | | | | _ Valor quantitativo, se disponível ____________________ | | | | | | | | | | | | | | | | | | | | | | | | | | | | | | | | | | | | |
| 26. Foi realizado o teste serológico número 1? | | | | | | | | | | □ Sim  □ Não | | | | | | | | | | | | | | 26.1 Se sim, qual ensaio?  □ ELISA Ig M  □ ELISA Ig G  □ RDT  □ IHA  □ IIF  □ CMIA  □ Outro, especifique_______________________ | | | | | | | | | | | | | | | | | | | | | | | | | | | | | | | | | | | | | |
| 26.2 Data do teste serológico número 1: | | | | | | | | | | | | | | | | | | | | | | | | | | | | \|__\|__\| - \|__\|__\|__\| - \|__\|__\|__\|__\| | | | | | | | | | | | | | | | | | | | | | | | | | | | | | | | | | |
| 26.3 Resultado | | | | | | □ Não detectável | | | | | | | | | | | | | □ Detectável | | | | | | | | | | | | | | | | | | | | | | | | Nome comercial___________________  Resultado quantitativo, se disponível_____________________  Valor de corte_______________________ | | | | | | | | | | | | | | | | | | |
|  | | | | | | | | | |  | | | | | | | | | | | | | | | | | | | | | | | | | | | | | | | | |  | | | | | | | | | | | | | | | | | | |
|  | | | | | | | | | |  | | | | | | | | | | | | | | | | | | | | | | | | | | | | | | | | |  | | | | | | | | | | | | | | | | | | |
| 27. Foi realizado o teste serológico número 2? | | | | | | | | | | □ Sim  □ Não | | | | | | | | | | | | | | 27.1 Se sim, qual ensaio?  □ ELISA Ig M  □ ELISA Ig G  □ RDT  □ IHA  □ IIF  □ CMIA  □ Outro, especifique_______________________ | | | | | | | | | | | | | | | | | | | | | | | | | | | | | | | | | | | | | |
| 27.2 Data do teste serológico número 2: | | | | | | | | | | | | | | | | | | | | | | | | | | | | \|__\|__\| - \|__\|__\|__\| - \|__\|__\|__\|__\| | | | | | | | | | | | | | | | | | | | | | | | | | | | | | | | | | |
| 27.3 Resultado | | | | | | □ Não detectável | | | | | | | | | | | | | □ Detectável | | | | | | | | | | | | | | | | | | | | | | | | Nome comercial___________________  Resultado quantitativo, se disponível_____________________  Valor de corte_______________________ | | | | | | | | | | | | | | | | | | |
| 28. Foi realizado o teste serológico número 3? | | | | | | | | | | □ Sim  □ Não | | | | | | | | | | | | | | 28.1 Se sim, qual ensaio?  □ ELISA Ig M  □ ELISA Ig G  □ RDT  □ IHA  □ IIF  □ CMIA  □ Outro, especifique_______________________ | | | | | | | | | | | | | | | | | | | | | | | | | | | | | | | | | | | | | |
| 28.2 Data do teste serológico número 3: | | | | | | | | | | | | | | | | | | | | | | | | | | | | \|__\|__\| - \|__\|__\|__\| - \|__\|__\|__\|__\| | | | | | | | | | | | | | | | | | | | | | | | | | | | | | | | | | |
| 28.3 Resultado | | | | | | □ Não detectável | | | | | | | | | | | | | □ Detectável | | | | | | | | | | | | | | | | | | | | | | | | Nome comercial___________________  Resultado quantitativo, se disponível_____________________  Valor de corte_______________________ | | | | | | | | | | | | | | | | | | |
| 29. Foi realizado o teste molecular 1? | | | | | | | | | | □ Sim  □ Não | | | | | | | | | | | | | | | | | | | | 29.1 Se sim, qual ensaio?  □ PCR convencional  □ qPCR  □ LAMP  □ Outro, especifique? _____________________________________ | | | | | | | | | | | | | | | | | | | | | | | | | | | | | | | |
| 29.2 Data do teste molecular 1 | | | | | | | | | | | | | | | | | | | | | | | | | | | | \|__\|__\| - \|__\|__\|__\| - \|__\|__\|__\|__\| | | | | | | | | | | | | | | | | | | | | | | | | | | | | | | | | | |
| 29.3 Resultado | | | | | | | | | | □ Não detectável | | | | | | | | | | | | | | | | | | | | | | | | | | | | | | | | | □ Detectável  Valor quantitativo, se disponível _____________________ | | | | | | | | | | | | | | | | | | |
| 30. Foi realizado o teste molecular 2? | | | | | | | | | | □ Sim  □ Não | | | | | | | | | | | | | | | | | | | | | | | | | | | | | | | | | 30.1 Se sim, qual ensaio?  □ PCR convencional  □ qPCR  □ LAMP  □ Outro, especifique? _____________________________________ | | | | | | | | | | | | | | | | | | |
| 30.2 Data do teste molecular 2 | | | | | | | | | | | | | | \|__\|__\| - \|__\|__\|__\| - \|__\|__\|__\|__\| | | | | | | | | | | | | | | | | | | | | | | | | | | | | | | | | | | | | | |  | | | | | | | | | |
| 30.3 Resultado | | | | | | | | | | □ Não detectável | | | | | | | | | | | | | | | | | | | | | | | | | | | | | | | | | □ Detectável  Valor quantitativo, se disponível _____________________ | | | | | | | | | | | | | | | | | | |
| 1. **Apresentação clínica Sintomas** | | | | | | | | | | | | | | | | | | | | | | | | | | | | | | | | | | | | | | | | | | | | | | | | | | | | | | | | | | | | | |
| 31. Agudo | □ Ignorado  □ Nenhum   \| □ Chagoma \| \| --- \| \| □ Mialgia \| \| □ Artralgia \| \| □ Esplenomegalia \| \| □ Astenia \| \| □ Dispneia  □ Dor de cabeça  □ Outro, especifique \| | | | | | | | | | | | | | | | | | | | | | | \| □ Sinal de Romana \| \| --- \| \| □ Dor abdominal \| \| □ Calafrios \| \| □ Hepatomegalia \| \| □ Adinamia \| \| \| □ Dor torácica  □ Linfadenopatia  □ Taquicardia \| \| | | | | | | | | | | | | | | | | | | | | | | | | | | \| □ Febre \| \| \| --- \| --- \| \| □ Edema facial \| \| \| □ Edema de membros inferiores \| \| \| \| □ Icterícia \| \| \| □ Prostração \| \| □ Nódulos cutâneos em membros inferiores \| \| \| | | | | | | | | | | | | |
| 32. Cardiovascular crônico | □ Ignorado  □ Nenhum   \| □ Lipotímia  □ Síncope \| \| --- \|   □ Palpitação  □ Edema periférico  □ Outro? Especifique | | | | | | | | | | | | | | | | | | | | | | Classificação NYHA:   \| □ Classe I \| \| --- \| \| □ Classe II  □ Classe III  □ Classe IV \|   □Fadiga | | | | | | | | | | | | | | | | | | | | | | | | | | □ Chiado  □ Inchaço abdominal  □ Ganho de peso rápido  □ Dispnea  □ Dor torácica  Evento tromboembólico   \| □ Sim \| \| --- \| \| □ Não  □ Desconhecido \| | | | | | | | | | | | | |
| 33. Digestivo crônico | □ Ignorado  □ Nenhum  □ Disfagia  □ Diarreia  □ Outro, especifique | | | | | | | | | | | | | | | | | | | | | | □ Odinofagia  □ Regurgitação | | | | | | | | | | | | | | | | | | | | | | | | | | Constipação   \| □ Sim  □ <7 dias □>7 dias \| \| --- \| \| □ Não \| | | | | | | | | | | | | |
| 34. Transmissão vertical (congênita) | □ Ignorado  □ Nenhum   \| □ Esplenomegalia  □ Linfadenopatia  □ Taquicardia  □ Cianose  □ Outro, especifique? \| \| --- \| | | | | | | | | | | | | | | | | | | | | | | \| □ Baixo peso ao nascer \| \| --- \| \| □ Prematuridade \| \| □ Polipneia \| \| □ Irritabilidade/Apatia  □ Icterícia  □ Convulsões \| | | | | | | | | | | | | | | | | | | | | | | | | | | □ Hepatomegalia   \| □ Febre \| \| --- \| \| □ Edema generalizado  □ Bradicardia  □ Microcefalia \| \|  \| | | | | | | | | | | | | |
|  | | | | | | | | | | | | | | | | | | | | | | | | | | | | | | | | | | | | | | | | | | | | | | | | | | | | | | | | | | | | | |
| **Sinais/Exame físico** | | | | | | | | | | | | | | | | | | | | | | | | | | | | | | | | | | | | | | | | | | | | | | | | | | | | | | | | | | | | | |
| 35. Peso (Kg) | | \|__\|__\|__\|.\|__ (Kg) | | | | | | | | | | | | | | | | | | | | | | | | | | 36. Altura (metros) | | | | | | | | | | | | | | | | | | | | | | | | \|__\|.\|__\|__\| (m) | | | | | | | | | |
| 37. Sinais vitais | | □ Ignorado  37.1 Pressão arterial | | | | | | | | | | | | | | | | □ Medido  \|__\|__\|__/__\|__\|__\| | | | | | | | | | | | | | | | | | | | | | 37.2 Temperatura (ºC) | | | | | | | | | | | | | | | | | \|__\|__\|__\| | | | | | |
|  |  | 37.3 Frequência respiratória | | | | | | | | | | | | | | | | | | | | | | | | \|__\|__\|__\| | | | | | | | | | | | | | 37.4 Frequência cardíaca | | | | | | | | | | | | | | | | | | | | \|__\|__\|__\| | | |
|  |  | 37.5 Saturação de oxigênio | | | | | | | | | | | | | | | | | | | | | | | | | | \|__\|__\|_%_\| | | | | | | | | | | | | | | | | | | | | | | | |  | | | | | | | | | |
| 38. Sinais de insuficiência cardíaca | | □ Ignorado  □ Nenhum   \| □ Edema de membros inferiores \| \| --- \| \| □ Taquicardia  □ Estertores  □ Outro, qual? \| | | | | | | | | | | | | | | | | | | | | | | | | | | □ Turgência jugular patológica  □ Hepatomegalia  □ Terceira bulha | | | | | | | | | | | | | | | | | | | | | | | | □ Ictus cordis deslocado e abaulado  □ Pulso irregular | | | | | | | | | |
| 1. **Resultado de exames** | | | | | | | | | | | | | | | | | | | | | | | | | | | | | | | | | | | | | | | | | | | | | | | | | | | | | | | | | | | | | |
| 39. Eletrocardiograma | | | | | | | | | | □ Não realizado  □ Alterações sugestivas de cardiomiopatia chagásica | | | | | | | | | | | | | | | | | | | | | | | | | | | | | | | | | □ Sem alterações  □ Alterações inespecíficas | | | | | | | | | | | | | | | | | | |
|  | | | | | | | | | |  | | | | | | | | | | | | | | | | | | | | | | | | | | | | | | | | | | | | | | | | | | | | | | | | | | | |
| 39.1 Data do eletrocardiograma | | | | | | | | | | | | | | | | | \|__\|__\| - \|__\|__\|__\| - \|__\|__\|__\|__\| | | | | | | | | | | | | | | | | | | | | | | | | | | | | | | | | | | | | | | | | | | | | |
| 39.2. ECG gravado está em ritmo de marca-passo | | | | | | | | | | | | | | | | | | | | □ Sim | | | | | | | | | | | | | | | | | | | | | | | | □ Não | | | | | | | | | | | | | | | | | |
| 39.2.1 Em caso afirmativo, especificar: | | | | | | | | | | | | | | | □ Marcapasspo unicameral | | | | | | | | | | | | | | | | | | | | | | | | | | | | | □ Marcapasspo bicameral | | | | | | | | | | | | | | | | | |
|  | | | | | | | □ ICD | | | | | | | | | | | | | | | | | | | | | | | | | | | | □ CRT-P | | | | | | | | | | | | | | | | □ CRT-D | | | | | | | | | | |
| 40. Se houver alterações sugestivas de cardiomiopatia chagásica: | | 40.1 Bloqueio de ramo direito | | | | | | | | | | | | | | | | | | | | | | | | | | | | | | | | | | | | | | | | | | | | | | | | | | | | | | | | | | □ Sim □ Não | |
|  |  | 40.2 Bloqueio de ramo esquerdo | | | | | | | | | | | | | | | | | | | | | | | | | | | | | | | | | | | | | | | | | | | | | | | | | | | | | | | | | | □ Sim □ Não | |
|  |  | 40.3 Bloqueio fascicular anterior esquerdo | | | | | | | | | | | | | | | | | | | | | | | | | | | | | | | | | | | | | | | | | | | | | | | | | | | | | | | | | | □ Sim □ Não | |
|  |  | 40.4 Extrassístoles ventriculares frequentes (mais de 1) | | | | | | | | | | | | | | | | | | | | | | | | | | | | | | | | | | | | | | | | | | | | | | | | | | | | | | | | | | □ Sim □ Não | |
|  |  | 40.5 Zonas eletricamente inativas (ondas Q ≥ 40 mseg e/ou com amplitude ≥ 25% da onda R em pelo 2 derivações contíguas) | | | | | | | | | | | | | | | | | | | | | | | | | | | | | | | | | | | | | | | | | | | | | | | | | | | | | | | | | | □ Sim □ Não | |
|  |  | 40.6 Bloqueio AV de segundo grau | | | | | | | | | | | | | | | | | | | | | | | | | | | | | | | | | | | | | | | | | | | | | | | | | | | | | | | | | | □ Sim □ Não | |
|  |  | 40.7 Bloqueio AV completo | | | | | | | | | | | | | | | | | | | | | | | | | | | | | | | | | | | | | | | | | | | | | | | | | | | | | | | | | | □ Sim □ Não | |
|  |  | 40.8 Taquicardia ventricular (sustentada ou não) | | | | | | | | | | | | | | | | | | | | | | | | | | | | | | | | | | | | | | | | | | | | | | | | | | | | | | | | | | □ Sim □ Não | |
|  |  | 40.9 Taquicardia e/ou fibrilação ou flutter atrial | | | | | | | | | | | | | | | | | | | | | | | | | | | | | | | | | | | | | | | | | | | | | | | | | | | | | | | | | | □ Sim □ Não | |
|  |  | 40.10 Bradicardia sinusal (<50 lpm) | | | | | | | | | | | | | | | | | | | | | | | | | | | | | | | | | | | | | | | | | | | | | | | | | | | | | | | | | | □ Sim □ Não | |
|  |  | 40.11 Alteração primária da repolarização ventricular | | | | | | | | | | | | | | | | | | | | | | | | | | | | | | | | | | | | | | | | | | | | | | | | | | | | | | | | | | □ Sim □ Não | |
|  |  | 40.12 Ritmo de marca-passo | | | | | | | | | | | | | | | | | | | | | | | | | | | | | | | | | | | | | | | | | | | | | | | | | | | | | | | | | | □ Sim □ Não | |
|  |  | 40.13 Outro, descreva________________________________________________ | | | | | | | | | | | | | | | | | | | | | | | | | | | | | | | | | | | | | | | | | | | | | | | | | | | | | | | | | | □ Sim □ Não | |
| 41. Ecocardiograma | | □ Não realizado | | | | | | | | | | | | | | | | | | | | | | | | | | □ Sem alterações | | | | | | | | | | | | | | | | | | | | | | | | □ Anormal | | | | | | | | | |
| 41.1 Data do ultrassom | | | | | | | | | | | | | | | | | \|__\|__\| - \|__\|__\|__\| - \|__\|__\|__\|__\| | | | | | | | | | | | | | | | | | | | | | | | | | | | | | | | | | | | | | | | | | | | | |
| 42. Dados do ecocardiograma | | | | | | | | | | 42.1 FE VE: \|__\|__\| % | | | | | | | | | | | | | | | | | | | | | | | | | | | Método: | | | | | | | | | | □ Simpson | | | | | | | | | | | □ Teicholtz | | | |
|  | | | | | | | | | | 42.2 Alterações contráteis segmentares | | | | | | | | | | | | | | | | | | | | | | | | | | | □ Sim | | | | | | | | | | □ Não | | | | | | | | | | | □ Ignorado | | | |
|  |  |  |  |  |  |  |  |  |  | 42.3 Dilatação do VE | | | | | | | | | | | | | | | | | | | | | | | | | | | □ Sim | | | | | | | | | | □ Não | | | | | | | | | | | □ Ignorado | | | |
|  |  |  |  |  |  |  |  |  |  | 42.4 Função sistólica do VE | | | | | | | | | | | | | | | | | | | | | | | | | | | □ Normal | | | | | | | | | | □ Anormal | | | | | | | | | | | □ Ignorado | | | |
|  |  |  |  |  |  |  |  |  |  | 42.4.1 Se anormal, | | | | | | | | | | | | | | | | | | | | | | | | | | | □ Leve | | | | | | | | | | □ Moderada | | | | | | | | | | | □ Grave | | | |
|  |  |  |  |  |  |  |  |  |  | 42.5 Função sistólica do VD | | | | | | | | | | | | | | | | | | | | | | | | | | | □ Normal | | | | | | | | | | □ Anormal | | | | | | | | | | | □ Ignorada | | | |
|  |  |  |  |  |  |  |  |  |  | 42.6 Aneurisma no VE? | | | | | | | | | | | | | | | | | | | | | | | | | | | □ Sim | | | | | | | | | | □ Não | | | | | | | | | | | □ Ignorado | | | |
|  |  |  |  |  |  |  |  |  |  | 42.7 Trombo ventricular | | | | | | | | | | | | | | | | | | | | | | | | | | | □ Sim | | | | | | | | | | □ Não | | | | | | | | | | | □ Ignorado | | | |
|  |  |  |  |  |  |  |  |  |  | 42.8 Doença orovalvar | | | | | | | | | | | | | | | | | | | | | | | | | | | □ Sim | | | | | | | | | | □ Não | | | | | | | | | | | □ Ignorado | | | |
|  |  |  |  |  |  |  |  |  |  | 42.8.1 Se houver doença orovalvar, pode ser atribuída à doença de Chagas? | | | | | | | | | | | | | | | | | | | | | | | | | | | | | | | | | | | | | □ Sim | | | | | | | | | | | □ Não | | | |
|  |  |  |  |  |  |  |  |  |  | 42.8.2 Especifique | | | | | | | | | | | | | | | | | | | | | | | | | | | □ Insuficiência mitral □ Insuficiência tricuspídea | | | | | | | | | | | | | | | | | | | | | | | | |
|  |  |  |  |  |  |  |  |  |  | 42.8.3 Grau de alteração valvular | | | | | | | | | | | | | | | | | | | | | | | | | | | □ Leve | | | | | | | | | | □ Moderado | | | | | | | | | | | □ Grave | | | |
|  |  |  |  |  |  |  |  |  |  | 42.9 Disfunção diastólica | | | | | | | | | | | | | | | | | | | | | | | | | | | □ Sim | | | | | | | | | | □ Não | | | | | | | | | | | □ Ignorado | | | |
|  |  |  |  |  |  |  |  |  |  | 42.9.1 Se houver disfunção diastólica | | | | | | | | | | | | | | | | | | | | | | | | | | | □ Tipo I | | | | | | | | | | □ Tipo II | | | | | | | | | | | □ Tipo III | | | |
|  |  |  |  |  |  |  |  |  |  | 42.10 Razão E/E’ \|__\|__\|.\|\|__\| | | | | | | | | | | | | | | | | | | | | | | | | | | |  | | | | | | | | | |  | | | | | | | | | | |  | | | |
|  |  |  |  |  |  |  |  |  |  | 42.11 Volume atrial esquerdo | | | | | | | | | | | | | | | | | | | | | | | | | | | \|__\|__\|.\|\|__\|mL/m^2^ | | | | | | | | | |  | | | | | | | | | | |  | | | |
|  |  |  |  |  |  |  |  |  |  | 42.12 Hipertensão pulmonar | | | | | | | | | | | | | | | | | | | | | | | | | | | □ Yes | | | | | | | | | | □ No | | | | | | | | | | | \|__\|__\|__\|mmHg | | | |
|  |  |  |  |  |  |  |  |  |  | 42.13 Outros achados | | | | | | | | | | | | | | | | | | | | | | | | | | | □ Sim | | | | | | | | | | □ Não | | | | | | | | | | |  | | | |
|  |  |  |  |  |  |  |  |  |  | 42.13.1 Se sim, especifique: | | | | | | | | | | | | | | | | | | | | | | | | | | |  | | | | | | | | | | | | | | | | | | | | | | | | |
| 43. Raio-x de tórax | | | | | | | | | | □ Não realizado | | | | | | | | | | | | | | | | | | | | | | | | | | | | | | | | | □ Realizado | | | | | | | | | | | | | | | | | | |
| 43.1 Data do raio-x de tórax | | | | | | | | | | | | | | | | | \|__\|__\| - \|__\|__\|__\| - \|__\|__\|__\|__\| | | | | | | | | | | | | | | | | | | | | | | | | | | | | | | | | | | | | | | | | | | | | |
| 43.2. ¿Cardiomegalia? | | | | | | | | | | □ Não  □ Sim  Índice cardiotorácico_________________ | | | | | | | | | | | | | | | | | | | | | | | | | | | | | | | | | | | | | | | | | | | | | | | | | | | |
| 44. Holter | | | | | | | | | | | | | | | □ Não realizado | | | | | | | | | | | | | | | | | | | | | | | | | | | | | □ Realizado | | | | | | | | | | | | | | | | | |
| 44.1 Data do Holter | | | | | | | | | | | | | | | \|__\|__\| - \|__\|__\|__\| - \|__\|__\|__\|__\| | | | | | | | | | | | | | | | | | | | | | | | | | | | | |  | | | | | | | | | | | | | | | | | |
| 44.2 Taquicardia ventricular sustentada | | | | | | | | | | | | | | | □ Sim | | | | | | | | | | | | | | | | | | | | | | | □ Não | | | | | | | | | | | | | | | | | □ Ignorado | | | | | | |
| 44.3 Taquicardia ventricular não sustentada | | | | | | | | | | | | | | | □ Sim | | | | | | | | | | | | | | | | | | | | | | | □ Não | | | | | | | | | | | | | | | | | □ Ignorado | | | | | | |
| 44.4 Fibrilação atrial | | | | | | | | | | | | | | | □ Sim | | | | | | | | | | | | | | | | | | | | | | | □ Não | | | | | | | | | | | | | | | | | □ Ignorado | | | | | | |
| 44.5 Bradicardia <40 bpm | | | | | | | | | | | | | | | □ Sim | | | | | | | | | | | | | | | | | | | | | | | □ Não | | | | | | | | | | | | | | | | | □ Ignorado | | | | | | |
| 44.6 Pausa sinusal > 3 segundos | | | | | | | | | | | | | | | □ Sim | | | | | | | | | | | | | | | | | | | | | | | □ Não | | | | | | | | | | | | | | | | | □ Ignorado | | | | | | |
| 45. RM cardíaca | | | | | | | | | | | | | | | □ Não realizado | | | | | | | | | | | | | | | | | | | | | | | | | | | | | □ Realizado | | | | | | | | | | | | | | | | | |
| 45.1 Data | | | | | | | | | | | | | | | \|__\|__\| - \|__\|__\|__\| - \|__\|__\|__\|__\| | | | | | | | | | | | | | | | | | | | | | | | | | | | | |  | | | | | | | | | | | | | | | | | |
| 45.2 Fibrose cardíaca | | | | | | | | | | | | | | | □ Sim | | | | | | | | | | | | | | | | | | | | | | | □ Não | | | | | | | | | | | | | | | | | □ Ignorado | | | | | | |
| 45.3 Massa de fibrose | | | | | | | | | | | | | | | \|__\|__\|__\| (g) | | | | | | | | | | | | | | | | | | | | | | | | | | | | |  | | | | | | | | | | | | | | | | | |
| 45.4 Aneurisma de VE | | | | | | | | | | | | | | | □ Sim | | | | | | | | | | | | | | | | | | | | | | | □ Não | | | | | | | | | | | | | | | | | □ Ignorado | | | | | | |
| 45.5 Trombo em VE | | | | | | | | | | | | | | | □ Sim | | | | | | | | | | | | | | | | | | | | | | | □ Não | | | | | | | | | | | | | | | | | □ Ignorado | | | | | | |
| 46. BNP e/ou NT-proBNP | | | | | | | | | | | | | | | □ Não realizado | | | | | | | | | | | | | | | | | | | | | | | | | | | | | □ Realizado | | | | | | | | | | | | | | | | | |
| 46.1 BNP e/ou NT-proBNP data | | | | | | | | | | | | | | | \|__\|__\| - \|__\|__\|__\| - \|__\|__\|__\|__\| | | | | | | | | | | | | | | | | | | | | | | | | | | | | | | | | | | | | | | | | | | | | | | |
| 46.1.1 BNP | | | | | | | | | | | | | | | □ Normal □ Anormal | | | | | | | | | | | | | | | | | | | | | | | \|__\|__\|__\|__\| (pg/mL) | | | | | | | | | | | | | | | | | □ Ignorado | | | | | | |
| 46.1.2 NT-proBNP | | | | | | | | | | | | | | | □ Normal □ Anormal | | | | | | | | | | | | | | | | | | | | | | | \|__\|__\|__\|__\| (pg/mL) | | | | | | | | | | | | | | | | | □ Ignorado | | | | | | |
| 1. **Classificações** | | | | | | | | | | | | | | | | | | | | | | | | | | | | | | | | | | | | | | | | | | | | | | | | | | | | | | | | | | | | | |
| 47. Classificação de Kuschnir | | | | | | | | | | | |  | | | | | | | | | □ 0 | | | | | | | | | | | | □ 1 | | | | | | | | | □ 2 | | | | | | | | | | □ 3 | | | | | | | | | □ Ignorado |
| 48. Classificação de consenso brasileiro | | | | | | | | | | | | □ FI | | | | | | | | | □ A | | | | | | | | | | | | □ B1 | | | | | | | | | □ B2 | | | | | | | | | | □ C  □ D | | | | | | | | | □ Ignorado |
| 49. Classificação latino-americana | | | | | | | | | | | | □ A | | | | | | | | | □ B1 | | | | | | | | | | | | □ B2 | | | | | | | | | □ C | | | | | | | | | | □ D | | | | | | | | | □ Ignorado |
| 50. Classificação da AHA | | | | | | | | | | | | □ A | | | | | | | | | □ B1 | | | | | | | | | | | | □ B2 | | | | | | | | | □ C | | | | | | | | | | □ D | | | | | | | | | □ Ignorado |
| 51. Classificação Los Andes | | | | | | | | | | | |  | | | | | | | | | □ IA | | | | | | | | | | | | □ IB | | | | | | | | | □ II | | | | | | | | | | □ III | | | | | | | | | □ Ignorado |
| **Achados digestivos** | | | | | | | | | | | | | | | | | | | | | | | | | | | | | | | | | | | | | | | | | | | | | | | | | | | | | | | | | | | | | |
| 52. Patologia digestiva detectada?? | | □ Sim | | | | | | | | | | | | | | | | | | | | | | | | | | □ Não | | | | | | | | | | | | | | | | | | | | | | | | □ Não investigado | | | | | | | | | |
| Se detectada, | |  | | | | | | | | | | | | | | | | | | | | | | | | | |  | | | | | | | | | | | | | | | | | | | | | | | |  | | | | | | | | | |
| 52.1 Megacólon | | □ Sim | | | | | | | | | | | | | | | | | | | | | | | | | | □ Não | | | | | | | | | | | | | | | | | | | | | | | | □ Não investigado | | | | | | | | | |
| 52.2 Megaesôfago | | □ Sim | | | | | | | | | | | | | | | | | | | | | | | | | | □ Não | | | | | | | | | | | | | | | | | | | | | | | | □ Não investigado | | | | | | | | | |
| 52.2.1 Classificação de Rezende | | | | | | | | | | | | □ E0 | | | | | | | | | □ EI | | | | | | | | | | | | □ EII | | | | | | | | | □ EIII | | | | | | | | | | □ EIV | | | | | | | | | □ Não realizado |
| **Classificação do estado clínico** | | | | | | | | | | | | | | | | | | | | | | | | | | | | | | | | | | | | | | | | | | | | | | | | | | | | | | | | | | | | | |
| 53. Forma clínica | | | | | | | | | | □ Controle  □ Crônica  □ Aguda | | | | | | | | | | | | | | | | | 52.1 Se crônica, especifique  □ Crônica com patologia não demonstrável (forma indeterminada)  □ Crônica com patologia cardíaca  □ Crônica com patologia digestiva  □ Crônica com patologia mista  52.2 Se aguda, especifique:  □ Primeira infecção  □ Reativação | | | | | | | | | | | | | | | | | | | | | | | | | | | | | | | | | | |
| 1. **Tratamento** | | | | | | | | | | | | | | | | | | | | | | | | | | | | | | | | | | | | | | | | | | | | | | | | | | | | | | | | | | | | | |
| 54. O paciente recebeu tratamento etiológico? | | | | | | | | | | □ Sim | | | | | | | | | | | | | | | | | | | | | | | | | | | | | | | | | □ Não | | | | | | | | | | | | | | | | | | |
| 54.1Se sim, qual é o status do tratamento?? | | | | | | | | | | | | | | | | | | | | | | | | | | | | | | | | | | | | | | | | □ Concluído | | | | | | | | | | □ Em andamento | | | | | | | | | | | □ Interrompido |
| Complete as seguintes informações apenas em caso de tratamento concluído ou interrompido: | | | | | | | | | | | | | | | | | | | | | | | | | | | | | | | | | | | | | | | | | | | | | | | | | | | | | | | | | | | | | |
| 54.2 Medicamento | | | | | | | | | | | | | | □ BNZ | | | | | | | | | | | | | | □ NFT | | | | | | | | | | | | | □ Outro, qual? __________________________ | | | | | | | | | | | | | | | | | | | | |
| 54.3 Dose total administrada | | | | | | | | | | | | | | | | | | | | | | | | | | | | \|__\|__\|__\|__\|__\| (mg) | | | | | | | | | | | | | | | | | | | | | | | | | | | | | | | | | |
| 54.4 Total de dias de tratamento | | | | | | | | | | | | | | | | | | | | | | | | | | | | \|__\|__\|__\| | | | | | | | | | | | | | | | | | | | | | | | | | | | | | | | | | |
| 54.5 Data de início | | | | | | | | | | | | | | | | | | | | | | | | | | | | \|__\|__\| - \|__\|__\|__\| - \|__\|__\|__\|__\| | | | | | | | | | | | | | | | | | | | | | | | | | | | | | | | | | |
| 54.6 Data de conclusão | | | | | | | | | | | | | | | | | | | | | | | | | | | | \|__\|__\| - \|__\|__\|__\| - \|__\|__\|__\|__\| | | | | | | | | | | | | | | | | | | | | | | | | | | | | | | | | | |
| 54.7 Se interrompido, especifique a causa da interrupção: _________________________________________  ___________________________________________________________________________________________________ | | | | | | | | | | | | | | | | | | | | | | | | | | | | | | | | | | | | | | | | | | | | | | | | | | | | | | | | | | | | | |
| 54.8 Houve eventos adversos associados ao tratamento relatados? | | | | | | | | | | | | | | | | | | | | | | | | | | | | | | | | □ Sim | | | | | | | | | | | | | | | | | | □ Não | | | | | | | | | | | |
| 54.8.1 Se sim, especifique _________________________________________________________________________________ | | | | | | | | | | | | | | | | | | | | | | | | | | | | | | | | | | | | | | | | | | | | | | | | | | | | | | | | | | | | | |
| 55. Uso de medicação cardiovascular? | | | | | | | | | | | | | | | | | | | | | | | | | | □ Sim | | | | | | | | | | | | | | | | | □ Não | | | | | | | | | | | | | | | | | | |
| 55.1 Em caso afirmativo, indicar quais: | | | | | | | | | | | | | | | | | | | | | | | | | | | | | | | | | | | | | | | | | | | | | | | | | | | | | | | | | | | | | |
| □ Beta-bloqueador | | | | | | | □ Inibidor da enzima de conversão da angiotensina | | | | | | | | | | | | | | | | | | | | | | | | | | | | □ Bloqueador de receptor da angiotensina | | | | | | | | | | | | | | | | □ Espironolactona | | | | | | | | | | |
| □ Sacubitril/valsartana | | | | | | | Inibidor do co-transportador de sódio-glicose (SGLT2) | | | | | | | | | | | | | | | | | | | | | | | | | | | | □ Ivabradina | | | | | | | | | | | | | | | | □ Furosemida | | | | | | | | | | |
| □ Digoxina | | | | | | | □ Anticoagulante oral | | | | | | | | | | | | | | | | | | | | | | | | | | | | □ Amiodarona | | | | | | | | | | | | | | | |  | | | | | | | | | | |
| 1. **Amostras biológicas** | | | | | | | | | | | | | | | | | | | | | | | | | | | | | | | | | | | | | | | | | | | | | | | | | | | | | | | | | | | | | |
| 56. Foram coletadas amostras biológicas?? | | | | | | | | | | | | | | | | | | | | | | | | | | □ Sim | | | | | | | | | | | | | | | | | □ Não | | | | | | | | | | | | | | | | | | |
| **Tipo de amostra**  Soro/plasma/sangue total/urina/saliva/tecido simples/PBMCs/ Outro, especifique | | | **Número de alíquotas** | | | | | | | | | | **Volume da amostra** | | | | | | | | | | | | | | | | | | **ID da amostra** | | | | | | | | | | | | **Data da coleta** | | | | | | | | | | | | | | | | | | |
|  | | |  | | | | | | | | | |  | | | | | | | | | | | | | | | | | |  | | | | | | | | | | | | \|__\|__\| - \|__\|__\|__\| - \|__\|__\|__\|__\| | | | | | | | | | | | | | | | | | | |
|  | | |  | | | | | | | | | |  | | | | | | | | | | | | | | | | | |  | | | | | | | | | | | | \|__\|__\| - \|__\|__\|__\| - \|__\|__\|__\|__\| | | | | | | | | | | | | | | | | | | |
|  | | |  | | | | | | | | | |  | | | | | | | | | | | | | | | | | |  | | | | | | | | | | | | \|__\|__\| - \|__\|__\|__\| - \|__\|__\|__\|__\| | | | | | | | | | | | | | | | | | | |
|  | | |  | | | | | | | | | |  | | | | | | | | | | | | | | | | | |  | | | | | | | | | | | | \|__\|__\| - \|__\|__\|__\| - \|__\|__\|__\|__\| | | | | | | | | | | | | | | | | | | |
|  | | |  | | | | | | | | | |  | | | | | | | | | | | | | | | | | |  | | | | | | | | | | | | \|__\|__\| - \|__\|__\|__\| - \|__\|__\|__\|__\| | | | | | | | | | | | | | | | | | | |

BNP, peptídeo natriurético cerebral; BNZ, benznidazol; CDI; desfibrilador cardíaco implantável; CRT-D, Ressincronizador com função de defibrlador; CRT-P, Ressincronizador com função de marcapasso; DPOC, doença pulmonary obstrutiva crônica; E, velocidade máxima do enchimento rápido no início da diástole; E’, velocidade máxima do deslocamento miocárdico no início da diástole; NFT, nifurtimox; RM, ressonância magnética; VD, ventrículo direito; VE, ventrículo esquerdo.
