# Supplementary material for: A standardized clinical database for research in Chagas disease: The NHEPACHA network
Source: PLoS Negl Trop Dis. 2024 Aug 15;18(8):e0012364. doi: 10.1371/journal.pntd.0012364 (PMC11326575; doi:10.1371/journal.pntd.0012364)
Supplement: S8 File — (DOCX) [file pntd.0012364.s008.docx]

**Instruções gerais**

No questionário clínico de pacientes com doença de Chagas, são solicitados dados específicos que resumem as informações detalhadas de um histórico clínico completo. Este guia representa um complemento para auxiliar na captura desses dados, tanto através da plataforma REDCap quanto no questionário físico.

No REDCap, algumas perguntas permitem marcar várias opções. Essas estão identificadas com uma caixa de seleção quadrada. As demais perguntas permitem apenas uma resposta e são identificadas com uma caixa de seleção circular. Se não houver informações suficientes para responder a alguma pergunta (por exemplo, se algum exame complementar não foi realizado), deve-se marcar a opção 'Ignorado'. Em todos os casos, evite o uso de abreviações ao preencher o questionário.

O questionário deve ser preenchido com as informações do estado de saúde atual do paciente no momento da coleta dos dados e das amostras. Quando solicitadas informações de laboratório e estudos de imagem, idealmente devem-se relatar aqueles obtidos nos últimos 6 meses.

Dentro do questionário digital no REDCap, alguns campos devem ser preenchidos a cada visita/revisão/seguimento agendado para o paciente e são identificados como 'Campo requerido'. Outros campos só serão obrigatórios na primeira vez em que o questionário for preenchido e são marcados como 'Coleta única'. Se alguma dessas seções precisar ser editada, isso será possível ao selecionar a opção 'Visualização prévia', seguida de 'Caso atual'. Isso permitirá visualizar e editar os dados já coletados.

A seguir, apresentam-se instruções para a coleta de algumas perguntas de particular importância. Estas estão organizadas por número de seção e pergunta. Caso tenha problemas ou dúvidas com alguma das perguntas não listadas abaixo, clique no símbolo
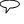
, ao lado da pergunta no questionário REDCap para questionar. Se estiver usando o questionário físico, entre em contato diretamente com um dos autores.

**Seção I: Dados da visita.**

Os três dados coletados nesta seção devem ser obtidos em cada visita.

Pergunta 1: O ID do paciente é gerado por cada instituição e representa o número usado para identificar o paciente, garantindo consistência em todas as suas visitas. O prefixo NEPACHA é o nome que identifica a instituição dentro da rede.

Pergunta 2: A data da visita refere-se ao momento em que o paciente é convocado para ser revisado. No questionário físico, deve ser registrada a data como dia, as três primeiras letras do mês e o ano completo. Por exemplo: 19-sep-2022

**Seção III. Dados do paciente.**

Embora não especificado no questionário, nem possível na plataforma REDCap, recomenda-se que neste ponto o entrevistador solicite o número de telefone do paciente ou de um contato próximo, bem como um endereço de e-mail, para garantir a comunicação com o paciente, se necessário.

**Seção IV: Informação epidemiológica.**

Pergunta 11: Ao solicitar o país de origem do paciente, busca-se determinar se ele é proveniente de uma área endêmica da doença de Chagas.

Pergunta 12: Caso o paciente não seja originário de nenhum país endêmico, é solicitado o país de origem da mãe para avaliar a possibilidade de transmissão vertical da infecção. No REDCap, isso ocorre automaticamente, porém, se estiver usando o formato físico, o entrevistador deve considerar que a pergunta 12 só é respondida se o paciente vier de uma área não endêmica.

Pergunta 13: Refere-se ao possível mecanismo de transmissão. É difícil estabelecer o mecanismo de transmissão com certeza, mas sugere-se investigar o máximo possível. A seguir, apresenta-se uma série de perguntas que podem orientar esse processo:

*Para investigar o mecanismo vetorial, sugerem-se as seguintes perguntas:*

- Você conhece o vetor (barbeiro/chinche/vinchuca/pito/chipo/pik)? Recomenda-se mostrar uma imagem com vários insetos, incluindo espécies encontradas na área de residência ou país de origem.
- Lembra-se da presença do vetor (nome pelo qual o participante conhece o vetor) dentro de casa ou residência?
- Lembra-se de ter visto o vetor no local de trabalho?
- Lembra-se de ter tido uma saliência associada à picada do vetor?
- Viu o vetor enquanto acampava ou estava no campo?

*Para investigar o mecanismo oral, sugerem-se as seguintes perguntas:*

- Você tem o hábito de consumir polpa de açaí, cana-de-açúcar ou suco de goiaba, guayaba ou outra fruta tropical?

*Para investigar o mecanismo vertical, sugerem-se as seguintes perguntas:*

- Sabe se sua avó materna, mãe ou irmãos foram diagnosticados com doença de Chagas?

*Para investigar o mecanismo de acidente laboral, sugere-se a seguinte pergunta:*

- Qual é sua ocupação? Além de trabalhadores de saúde/pesquisadores que manipulam amostras, considere como risco os caçadores que pernoitam na mata, os que manipulam carne e sangue de animais para consumo humano não regulamentado.

*Para os mecanismos transfusional e de transplante, sugerem-se as seguintes perguntas:*

- Recebeu transfusão sanguínea? Por qual motivo? Data em que foi feita?
- Recebeu transplante? Por qual motivo? Data em que foi feito?

Pergunta 24: O significado das siglas dos dispositivos intracardíacos se encontra ao final do questionário. Recomenda-se que o entrevistador solicite ao paciente o cartão do dispositivo porque lá encontra-se especificado o tipo de dispositivo ou busque a informação no prontuário do paciente.

**Seção V: Diagnóstico etiológico.**

Nesta seção, deve-se indicar todos os exames realizados no paciente para alcançar o diagnóstico etiológico. Isso inclui testes parasitológicos, sorológicos e moleculares. No caso dos testes sorológicos, pelo menos dois resultados positivos são necessários para estabelecer o diagnóstico. O questionário permite inserir informações de até três testes sorológicos e dois moleculares. Nos testes sorológicos, o valor de corte refere-se ao valor acima do qual o resultado do teste é considerado positivo.

**Seção VI: Quadro clínico.**

Pergunta 32: Esta pergunta inclui a avaliação da presença de sinais clínicos de insuficiência cardíaca conforme a escala da New York Heart Association (NYHA), apresentada a seguir.

| Classificação funcional da gravidade da dispneia de acordo com a New York Heart Association (NYHA) | |
| --- | --- |
| Grau I | O paciente apresenta dispneia ao realizar grandes esforços, como correr, subir vários lances de escadas ou praticar esportes intensos, atividades que anteriormente podia fazer sem desconforto. |
| Grau II | El paciente presenta disnea al realizar esfuerzos moderados como caminar, correr una distancia corta o subir un piso de escalera. |
| Grau III | O paciente apresenta dispneia ao realizar esforços moderados, como caminhar, correr uma curta distância ou subir um lance de escadas. |
| Grau IV | O paciente apresenta dispneia durante o repouso físico e mental. |

Pergunta 37. O questionário da plataforma REDCap só detalhará a captura dos sinais vitais se a opção "Medidos" for marcada nesta pergunta.

**Seção VII: Resultados de exames.**

Perguntas 39 e 40: Estas perguntas referem-se à presença de sinais eletrocardiográficos específicos da cardiomiopatia chagásica. Se o paciente apresentar alguma alteração eletrocardiográfica não listada na pergunta 40 do questionário, selecione a opção "Alterações inespecíficas" na pergunta 39. Se o paciente apresentar alguma alteração eletrocardiográfica listada na pergunta 40, por favor, marque-a na caixa correspondente.

Perguntas 41, 42, 43,44, 45 e 46: Estas perguntas se referem aos dados do ecocardiograma, radiografia de tórax, Holter, ressonância e nível sérico de NNP e/ou NT-proBNP. Todos os dados devem ser coletados de todos os pacientes, sempre que disponível.

Perguntas 41 e 42: Se o paciente apresentar alguma alteração não listada na pergunta 42, indique a opção "presença de alterações não chagásicas" na pergunta 41. Se o doente apresentar alguma alteração ecocardiográfica listada na pergunta 42, por favor assinale-a na casa correspondente.

Para a pergunta 46, seguem-se os valores BNP e NT-proBNP:

| **Cenário clínico** | **BNP** | **NT-proBNP** |
| --- | --- | --- |
| Pacientes não agudos | < 35 pg/mL | < 125 pg/mL |
| Pacientes agudos | < 100 pg/mL | < 300 pg/mL |
| Meia-vida | 20 minutos | 120 minutos |

**Seção VIII: Classificações.**

A seguir, é apresentada uma tabela para ajudar a classificar clinicamente um paciente de acordo com as diferentes classificações incluídas na seção VIII.

Perguntas 47-51:

| Classificações da doença de Chagas de acordo com alterações em exames complementares e sintomas. | | | | | |
| --- | --- | --- | --- | --- | --- |
| Alterações em exames complementares e sintomas | Classificações | | | | |
|  | Kuschnir | Consenso Brasileiro | Los Andes | Latino-americana | AHA |
| ECG normal e aparentemente sem EEC | 0 | NA | IA | A | A^a^ |
| ECG normal com anomalias contráteis | NA | NA | IB | NA | B1 |
| ECG anormal aparentemente sem EEC | I | A | NA | B1 | B1 |
| ECG anormal, anomalias contráteis com FEVI normal | NA | B1 | II | B1 | B1 |
| ECG anormal, provável EEC ou FEVI anormal | II | B1 (FEVI ≥ 45%), B2 (FEVI ≤ 45%) | II | B2 | B2 |
| Insuficiência cardíaca descompensada | III | C (compensado) | III | C (compensado) | C (compensado) |
| Insuficiência cardíaca refratária. | NA | D (refractária) | NA | D (refractária) | D (refractária) |
| ECC: eletrocardiograma; EEC: doença estrutural cardíaca; FEVI: fração de ejeção do ventrículo esquerdo; NA: não aplicável; AHA: Associação Americana do Coração; a: sem alteração digestiva. | | | | | |

Pergunta 52.2.1:

Classificação de Rezende para a acalásia do esôfago

| Classificação Rezende | |
| --- | --- |
| Grado 1 | Forma inicial, corpo esofágico com diâmetro < 4 cm. |
| Grado 2 | Esôfago dilatado com diâmetro > 4 cm, mas < 7 cm. |
| Grado 3 | Diâmetro esofágico entre 7 e 10 cm. |
| Grado 4 | Diâmetro > 10 cm no qual pode ser observado um eixo sinuoso característico de um dolicoesôfago. |

Pergunta 53 Pacientes diagnosticados com infecção por *Trypanosoma cruzi* devem ser classificados como agudos ou crônicos, com base na sintomatologia e no tempo desde a possível infecção. Se o paciente for classificado como crônico, o tipo de dano orgânico detectado deve ser especificado. Na plataforma REDCap, essas opções só serão exibidas se a caixa "Crônico" for selecionada.

Se o participante fizer parte do grupo controle de um estudo de pesquisa clínica, a caixa correspondente deve ser marcada.

**Seção IX: Tratamento.**

Pergunta 54: Se o paciente recebeu tratamento etiológico com benznidazol ou nifurtimox, ou algum medicamento antiparasitário experimental no contexto de um ensaio clínico, a caixa "Sim" deve ser marcada. As perguntas 54.1-54.8 devem ser respondidas apenas neste caso.
Pergunta 54.1 refere-se ao estado atual do tratamento e se o paciente concluiu o esquema completo de tratamento, interrompeu antes da conclusão ou ainda está em andamento (Em curso). Na plataforma REDCap, as perguntas 54.1-54.8 serão exibidas somente se "Sim" for selecionado na pergunta 53.

Pergunta 55.1: Marcar todas as medicações de uso cardiovascular que o paciente esteja usando na data da visita.

**Seção X: Amostras biológicas**

Pergunta 56: Nesta pergunta, deve-se indicar se foram coletadas amostras biológicas do paciente. Em caso afirmativo, é necessário preencher a tabela correspondente. Na plataforma REDCap, essa tabela será exibida somente se "Sim" for marcado na pergunta 55. Neste ponto, serão solicitadas informações sobre o tipo de amostra, número de alíquotas, volume, identificador e data de coleta da amostra. Para preencher esta seção, é recomendável buscar apoio do responsável pela coleta das amostras.

**Glossário**

BNP, peptídeo natriurético cerebral.

BNZ, benznidazol.

CDI; desfibrilador cardíaco implantável.

CRT-D, Ressincronizador com função de defibrilador.

CRT-P, Ressincronizador com função de marcapasso.

DPOC, doença pulmonary obstrutiva crônica.

E, velocidade máxima do enchimento rápido no início da diástole.

E’, velocidade máxima do deslocamento miocárdico no início da diástole.

NFT, nifurtimox.

RM, ressonância magnética.

VD, ventrículo direito.

VE, ventrículo esquerdo.
